# Comparative Safety of JAK Inhibitors vs TNF Antagonists in Immune-Mediated Inflammatory Diseases: A Systematic Review and Meta-Analysis

**DOI:** 10.1001/jamanetworkopen.2025.31204

**Published:** 2025-09-10

**Authors:** Virginia Solitano, Dhruv Ahuja, Han Hee Lee, Ritu Gaikwad, Kuan-Hung Yeh, Antonio Facciorusso, Abha G. Singh, Christopher Ma, Ashwin N. Ananthakrishnan, Yuhong Yuan, Namrata Singh, Vipul Jairath, Siddharth Singh

**Affiliations:** 1 Division of Gastroenterology and Gastrointestinal Endoscopy, IRCCS Ospedale San Raffaele, University Vita-Salute San Raffaele, Milan, Italy; 2 Department of Epidemiology and Biostatistics, Western University, London, Ontario, Canada; 3 Division of Gastroenterology, Department of Medicine, University of California San Diego, La Jolla; 4 Department of Medicine, Indira Gandhi Hospital, New Delhi, India; 5 Division of Gastroenterology, Department of Internal Medicine, Yeouido St Mary’s Hospital College of Medicine, The Catholic University of Korea, Seoul, South Korea; 6 California Northstate University School of Medicine, Elk Grove; 7 Division of Biomedical Informatics, Department of Medicine, University of California San Diego, La Jolla; 8 Gastroenterology Unit, Department of Medical and Surgical Sciences, University of Foggia, Foggia, Italy; 9 Division of Rheumatology, Autoimmunity, and Inflammation, Department of Medicine, University of California San Diego, La Jolla; 10 Division of Gastroenterology and Hepatology, Cumming School of Medicine, University of Calgary, Calgary, Alberta, Canada; 11 Department of Community Health Sciences, University of Calgary, Calgary, Alberta, Canada; 12 Division of Gastroenterology, Massachusetts General Hospital, Boston; 13 Harvard Medical School, Boston, Massachusetts; 14 Division of Gastroenterology, Western University, London, Ontario, Canada; 15 Division of Rheumatology, University of Washington, Seattle

## Abstract

**Importance:**

Janus kinase (JAK) inhibitors are highly effective medications for several immune-mediated inflammatory diseases (IMIDs). However, safety concerns have led to regulatory restrictions.

**Objective:**

To compare the risk of adverse events with JAK inhibitors vs tumor necrosis factor (TNF) antagonists in patients with IMIDs in head-to-head comparative effectiveness studies.

**Data Sources:**

For this systematic review and meta-analysis, the Ovid Medline, Ovid EMBASE, and Web of Science databases were searched from inception to June 25, 2025.

**Study Selection:**

Head-to-head comparative effectiveness studies of adults (aged ≥18 years) with IMIDs (including rheumatoid arthritis, inflammatory bowel disease, psoriasis or psoriatic arthritis, or spondyloarthropathy) treated with either JAK inhibitors or TNF antagonists were included. Randomized clinical trials, noncomparative observational studies, studies not reporting outcomes of interest or focused solely on specific safety events, and studies with a sample size of less than 500 were excluded. The Preferred Reporting Items for Systematic Reviews and Meta-Analyses (PRISMA) reporting guideline was followed.

**Data Extraction and Synthesis:**

Four investigators independently, and in pairs, abstracted data from included studies. A random-effects meta-analysis was conducted to obtain incidence rates (IRs) and hazard ratios (HRs) for JAK inhibitors vs TNF antagonists for each safety outcome (serious infections, malignant neoplasms, major cardiovascular events [MACEs], or venous thromboembolism [VTE]), adjusting for key confounding variables. Heterogeneity was quantified using the *I*^2^ statistic. Risk of bias was assessed by 2 investigators independently using the Newcastle-Ottawa Scale.

**Main Outcomes and Measures:**

The primary outcome was risk of serious infections, malignant neoplasms, MACEs, or VTE.

**Results:**

This meta-analysis of 42 studies with low to moderate risk of bias included 813 881 patients (median age, 55.7 years [IQR, 53.0-59.0 years] for JAK inhibitor users and 51.5 years [IQR, 42.7-57.4 years] for TNF antagonist users; 76.5% female). For patients using JAK inhibitors vs TNF antagonists, there was no significant difference in risk of serious infections (IR, 3.79 [95% CI, 2.85-5.05] vs 3.03 [2.32-3.95] per 100 person-years; pooled HR, 1.05 [95% CI, 0.97-1.13]), malignant neoplasms (IR, 1.00 [0.77-1.31] vs 0.94 [0.72-1.22] per 100 person-years; pooled HR, 1.02 [0.90-1.16]), or MACEs (IR, 0.72 [0.56-0.92] vs 0.66 [0.49-0.89] per 100 person-years; pooled HR, 0.91 [0.80-1.04]), with minimal to moderate heterogeneity. There was a slightly higher risk of VTE with JAK inhibitors vs TNF antagonists (IR, 0.57 [95% CI, 0.40-0.82] vs 0.52 [0.37-0.73] per 100 person-years; pooled HR, 1.26 [95% CI, 1.03-1.54]). Effect estimates were largely stable across subgroups and on meta-regression.

**Conclusions and Relevance:**

The head-to-head studies in this systematic review and meta-analysis did not identify any meaningful difference in the risk of serious infections, malignant neoplasms, or MACEs with JAK inhibitor vs TNF antagonist use across all IMIDs, with low overall incidence. JAK inhibitor use was associated with a slightly higher risk of VTE. Further research, especially long-term studies, is needed to fully elucidate the safety of JAK inhibitors and TNF antagonists across diverse populations and optimize clinical use.

## Key Points

**Question:**

What are the comparative safety profiles of Janus kinase (JAK) inhibitors vs tumor necrosis factor (TNF) antagonists in patients with immune-mediated inflammatory diseases (IMIDs)?

**Findings:**

In this systematic review and meta-analysis including 42 head-to-head comparative effectiveness studies of 813 881 patients with IMIDs treated with JAK inhibitors or TNF antagonists, no meaningful differences in risk of serious infections, malignant neoplasms, and major cardiovascular events were observed. JAK inhibitor use was associated with a slightly higher risk of venous thromboembolism compared with TNF antagonist use; the overall incidence of serious adverse events was low.

**Meaning:**

These findings call for revisiting the strict regulatory guidance imposed by the US Food and Drug Administration and the European Medicines Agency restricting use of all JAK inhibitors after failure of, or contraindications to, TNF antagonists, across all indications.

## Teaser

This systematic review and meta-analysis of head-to-head comparative effectiveness studies examines safety outcomes in patients with immune-mediated inflammatory diseases (IMIDs) treated with Janus kinase (JAK) inhibitors vs tumor necrosis factor (TNF) antagonists.

## Introduction

The selection of advanced therapeutics for immune-mediated inflammatory disorders (IMIDs) requires a careful balance between effectiveness and safety, among other factors. Janus kinase (JAK) inhibitors have emerged as an important treatment option, offering rapid and targeted immunomodulation.^1^ However, in 2019, regulatory agencies modified the label for JAK inhibitors based on the ORAL Surveillance trial.^2^ The noninferiority ORAL Surveillance trial compared the safety of tofacitinib (5 mg or 10 mg twice daily) vs tumor necrosis factor (TNF)-α antagonists in patients with rheumatoid arthritis (RA) who were aged 50 years or older and had at least 1 additional cardiovascular risk factor. Over a median follow-up of 4 years, tofacitinib was associated with a higher risk of major adverse cardiovascular events (MACEs) (3.4% vs 2.5%; hazard ratio [HR], 1.33), venous thromboembolism (VTE), cancer, and death. Following this, the US Food and Drug Administration changed the labeling of JAK inhibitors across all indications, restricting their use in patients who experienced prior treatment failure with or intolerance to TNF antagonists.^3^ The European Medicines Agency recommended that for patients aged 65 years or older with a history of smoking or risk factors for cardiovascular disease or malignant neoplasms, tofacitinib should be used only if no suitable alternatives exist.^4^

The ORAL Surveillance trial focused on older high-risk patients with RA, limiting the applicability of the trial findings to broader IMID populations and all JAK inhibitors. Given the growing use of JAK inhibitors to treat inflammatory bowel disease (IBD), psoriasis (PsO) or psoriatic arthritis (PsA), and spondyloarthropathy (SpA), a critical safety evaluation is needed.^5^ Clinical decision-making requires assessing absolute risks alongside effectiveness. Thus, we conducted a systematic review and meta-analysis of head-to-head comparative effectiveness studies of JAK inhibitors vs TNF antagonists for serious infections, malignant neoplasms, MACEs, and VTE to provide a comprehensive safety assessment across IMIDs.

## Methods

This systematic review and meta-analysis of previously published studies did not involve direct interaction with human participants or access to identifiable personal data; therefore, the University of California San Diego Institutional Review Board deemed the study exempt from review and waived the requirement of informed consent. We used a protocol established a priori, provided in the eMethods in Supplement 1. The study followed the Preferred Reporting Items for Systematic Reviews and Meta-Analyses (PRISMA) reporting guideline.

### Data Sources and Searches

We systematically searched the Ovid Medline, Ovid EMBASE, and Web of Science databases from inception to June 25, 2025, for studies on risk of serious infections, cancer, MACEs, and VTE in adults (aged ≥18 years) with IMIDs treated with JAK inhibitors or TNF antagonists. The search strategy is provided in the eAppendix in Supplement 1.

### Study Selection

Two authors (V.S. and S.S.) independently reviewed the titles and abstracts to exclude irrelevant studies based on predefined inclusion and exclusion criteria. These two authors also independently reviewed full texts of the remaining studies for eligibility.

We included head-to-head comparative effectiveness studies of adults with IMIDs (including RA, IBD, PsO or PsA, or SpA) treated with JAK inhibitors or TNF antagonists that reported risk of serious infections, malignant neoplasms, MACEs, or VTE. We excluded randomized clinical trials, noncomparative observational studies (eg, studies evaluating safety risk with JAK inhibitors vs a nonexposed group), studies that did not report outcomes of interest or focused solely on specific safety events (eg, zoster or specific opportunistic infections), and studies with a sample size of less than 500. Additional details on study selection are reported in the eMethods in Supplement 1.

Four investigators (V.S., D.A., H.H.L., and R.G.) independently, and in pairs, abstracted data from included studies using a standardized data abstraction form, after initially piloting the form. Risk of bias was assessed by investigators (V.S. and H.H.L.) independently using the Newcastle-Ottawa Scale.^6^

### Primary Outcome

The primary outcome was risk of serious infections (requiring hospitalization, intravenous antibiotics, or therapy cessation or causing death), malignant neoplasms (excluding nonmelanoma skin cancer), MACEs (myocardial infarction, stroke, or cardiovascular mortality), and VTE (pulmonary embolism or deep venous thrombosis). Outcomes were mostly identified via administrative claims codes.

### Statistical Analysis

Complete details of the analytic approach are reported in the eMethods in Supplement 1 and summarized here. To assess the stability of JAK inhibitors vs TNF antagonists on safety outcomes and explore heterogeneity, we performed prespecified subgroup analyses by study location, IMID type, JAK inhibitor type, age group, and outcome subtype. Meta-regression was used to examine study-level factors, including age, sex distribution, study recruitment midpoint (to assess regulatory impact), concomitant immunosuppressive use, and major comorbidities. Sensitivity analyses excluded studies lacking key confounder adjustments.

We performed a random-effects meta-analysis, as described by Hartung and Knapp,^7^ to estimate summary risk of adverse safety outcomes by calculating the pooled estimates of HRs and their 95% CIs for safety outcomes in patients treated with JAK inhibitors vs TNF antagonists. To account for confounding, we used HRs adjusted for covariates. In cases where HRs were not available, we estimated unadjusted incidence rate ratios (IRRs) based on the reported incidence rates (IRs) of events in each treatment group. Effect estimates and their SEs were incorporated into the meta-analysis using the inverse-variance method, which assigns weights to individual studies according to the precision of their estimates.

Heterogeneity across studies was assessed using the *I*^2^ statistic, which quantifies the percentage of total variation across studies attributed by heterogeneity between studies rather than sampling error. *I*^2^ values were calculated using the χ^2^ test and degrees of freedom (<30%, 30%-60%, 60%-75%, and >75% indicated low, moderate, substantial, and considerable heterogeneity, respectively).^8,9^ Two-sided *P* < .10 for differences between subgroups was considered statistically significant. Publication bias was assessed qualitatively using funnel plots when more than 10 studies were identified for a comparison, and asymmetry of funnel plots was quantitatively assessed using the Egger test of the intercept.^10^ All analyses were performed using the meta package in R, version 4.3.2 (R Project for Statistical Computing).

## Results

### Study and Patient Characteristics

The systematic literature review identified 5659 unique articles, of which full texts of 88 articles were reviewed in detail (eFigure 1 in Supplement 1). Of these, 41 published studies were included.^11,12,13,14,15,16,17,18,19,20,21,22,23,24,25,26,27,28,29,30,31,32,33,34,35,36,37,38,39,40,41,42,43,44,45,46,47,48,49,50,51^ Additionally, data from 1 unpublished cohort from the investigator team was also included.^52^ Hence, data from 42 head-to-head comparative effectiveness studies were used for quantitative synthesis, representing 813 881 patients (128 705 treated with JAK inhibitors compared with 685 176 treated with TNF antagonists). The median age was 55.7 years (IQR, 53.0-59.0 years) for JAK inhibitor users and 51.5 years (IQR, 42.7-57.4 years) for TNF antagonist users; 76.5% of patients were female and 23.5% were male.

Several studies included multiple databases, each representing a distinct population. Therefore, analyses were conducted at the cohort level rather than the study level. In total, 18 cohorts evaluated the risk of serious infections,^11,12,15,17,20,28,29,30,31,36,38,40,48,51,52^ 17 cohorts examined the risk of cancer,^20,23,26,28,34,36,37,38,39,40,41,42,45^ 29 cohorts assessed the risk of MACEs,^12,13,16,18,19,22,24,25,27,28,31,32,33,36,37,39,41,44,46,47,48,49,50,51,52^ and 16 cohorts compared the risk of VTE^12,14,18,21,22,32,36,37,41,43,49,50,51,52^ with JAK inhibitors vs TNF antagonists.

eTable 1 in Supplement 1 presents study-level characteristics of the included studies. Most of the studies (n = 35 of 42 [83.3%]) involved retrospective cohorts,^11,12,13,14,15,17,18,20,22,23,24,25,26,27,28,29,30,31,32,33,36,37,38,39,41,42,43,44,45,46,47,48,49,50,52^ whereas the remainder (n = 7 [16.7%]) used prospective cohorts.^16,19,21,34,35,40,51^ A total of 20 studies (47.6%) used administrative data,^11,12,13,14,15,18,22,23,25,29,30,33,37,42,43,44,45,47,50,52^ 20 (47.6%) relied on national health care databases,^13,14,16-18,21,23-25,28,29,31-33,36,38,43,45,46,48^ and 2 (4.8%) used medical record reviews.^38,40^ Most of the studies were conducted in North America (n = 12 [28.6%]),^11,15,22,24,30,33,36,42,43,44,45,52^ Europe (n = 12 [28.6%]),^16,19,20,21,26,29,31,32,34,35,39,49^ or Asia (n = 14 [33.3%])^13,14,17,18,23,27,28,37,38,40,41,48,50,51^; a few (n = 4 [9.5%]) were transcontinental.^12,25,46,47^ Propensity score (PS) methods were used in 22 studies (52.4%),^11,12,13,14,17,18,19,22,23,25,29,30,32,33,40,41,42,43,44,45,47,52^ whereas 15 studies (35.7%) applied a Cox proportional hazards regression model without PS^15,16,20,21,24,26,28,31,34,35,36,37,38,49,50^ and 5 (11.9%) reported adjusted IRs, unadjusted IRs, or both.^27,39,46,48,51^ The median follow-up was 23 months (IQR, 16-35 months) for patients treated with JAK inhibitors and 22 months (IQR, 14-34 months) for those treated with TNF antagonists.

eTable 2 in Supplement 1 presents the baseline characteristics of patients in each intervention arm across the included studies. In terms of IMIDs studied, 38 studies focused on RA,^11,12,13,14,15,16,17,18,19,21,23,24,25,26,27,28,31,32,33,35,36,37,38,39,40,41,42,43,44,45,46,49,50,51^ 4 on IBD,^22,30,48,52^ 4 on PsA,^20,29,34,47^ and 2 on SpA.^20,47^ Patient characteristics were generally similar, especially in PS-matched cohorts.

eTable 3 in Supplement 1 presents the risk of bias assessment for the cohort studies. Overall, the studies had a low to moderate risk of bias.

### Risk of Serious Infections

**Figure 1.**
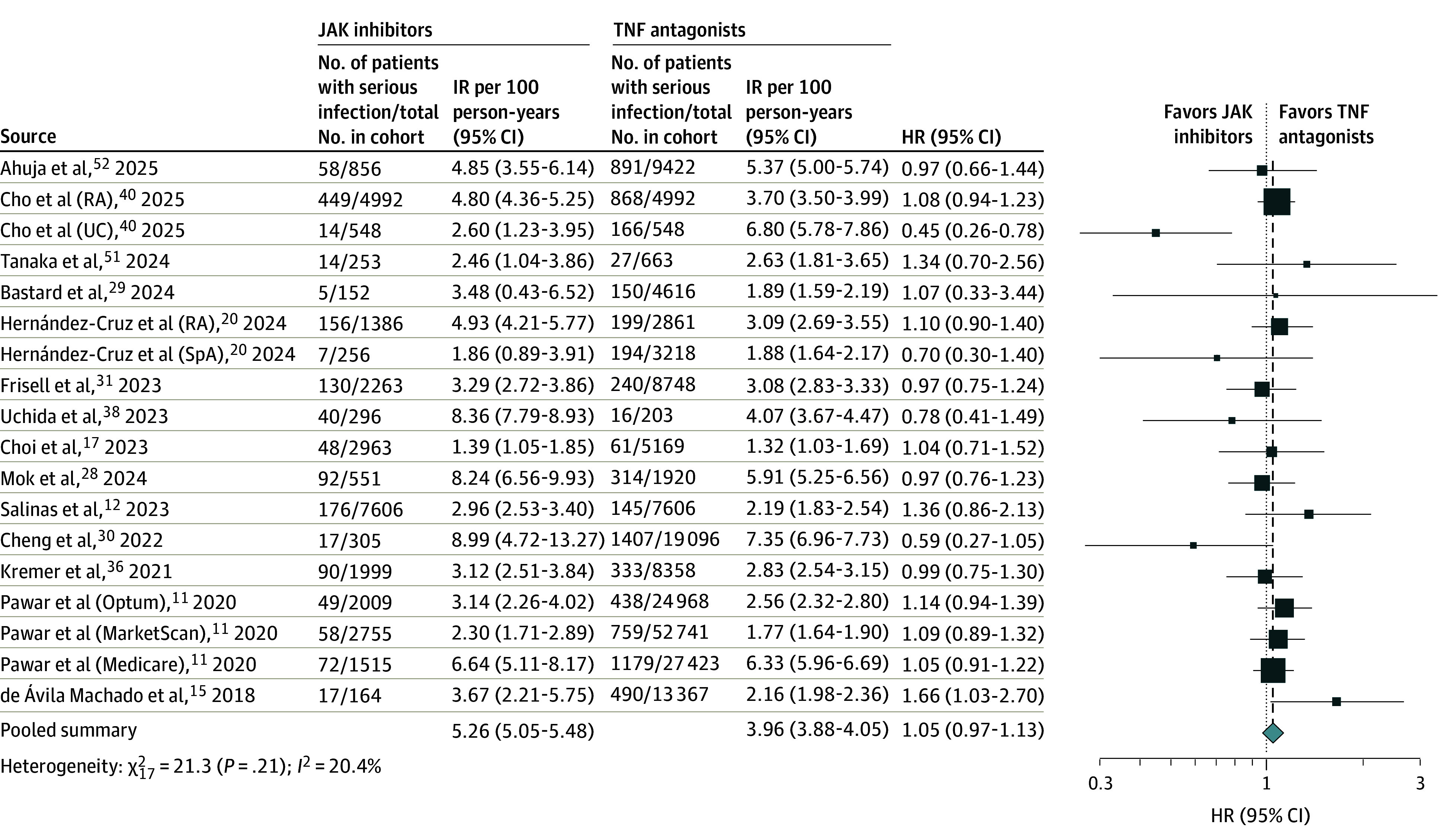
Risk of Serious Infections With Janus Kinase (JAK) Inhibitors vs Tumor Necrosis Factor (TNF) Antagonists in All Patients With Immune-Mediated Inflammatory Diseases Box represents the effect size estimate from the individual study included in the meta-analysis. The size of the box corresponds to the weight of that study's estimate, with larger boxes indicating more weight or precision. Horizontal lines extending from the box represent the CI for that study's effect. HR indicates hazard ratio; IR, incidence rate; RA, rheumatoid arthritis; SpA, spondyloarthropathy; UC, ulcerative colitis.

The meta-analysis included 18 cohorts involving a total of 226 788 patients (30 869 treated with JAK inhibitors vs 195 919 treated with TNF antagonists), with 9369 case patients with serious infections.^11,12,15,17,20,28,29,30,31,36,38,40,48,51,52^ The IR of serious infections was 3.79 (95% CI, 2.85-5.05) per 100 person-years with JAK inhibitors vs 3.03 (95% CI, 2.32-3.95) per 100 person-years with TNF antagonists. There was no statistically significant difference in the risk of serious infections between JAK inhibitors vs TNF antagonists (pooled HR, 1.05 [95% CI, 0.97-1.13]), with low heterogeneity (*I*^2^ = 20.4%) (Figure 1).

Overall findings remained stable on subgroup analyses stratified by geographic location, IMID type, and JAK inhibitor type (Table and eFigures 2-4 in Supplement 1). On meta-regression, a higher proportion of males and a higher prevalence of concomitant immunomodulator use was associated with increased risk of serious infections with JAK inhibitors vs TNF antagonists (eTable 4 in Supplement 1). Mean age, midpoint of study recruitment, concomitant corticosteroid use, prevalence of major comorbidities (eg, diabetes, hypertension, hyperlipidemia, prior cardiovascular disease, or obesity), prior infections, and smoking did not change the direction of the association (eTable 4 in Supplement 1). We did not observe any evidence of publication bias (Egger regression coefficient, *P* = .22) (eFigure 5 in Supplement 1).

**Table.** Subgroup Analysis With Different Outcomes

Variable	No. of cohorts	IR per 100 person-years		JAK inhibitors vs TNF antagonists, HR (95% CI)	*I* ^2^	*P* values for difference in subgroups
		JAK inhibitors	TNF antagonists			
**Serious infections**						
Geographic location						
Asia	7	3.79 (2.2-6.49)	3.44 (2.24-5.3)	0.97 (0.72-1.30)	49.9	.65
North America	8	3.83 (2.85-5.15)	3.34 (2.36-5.0)	1.08 (0.97-1.20)	4.8	
Europe	5	3.41 (2.55-4.55)	2.38 (1.9-2.97)	1.11 (0.85-1.46)	38.5	
IMID type						
Rheumatoid arthritis	13	3.72 (2.78-4.98)	2.93 (2.3-3.7)	1.07 (1.01-1.14)	0	.15
Spondyloarthritis	1	1.86 (0.89-3.91)	1.88 (1.64-2.17)	0.70 (0.3-1.40)	0	
Psoriatic arthritis	1	3.48 (0.43-6.52)	1.89 (1.59-2.19)	1.07 (0.33-3.44)	0	
IBD	3	4.4 (2.22-8.72)	6.41 (5.28-6.82)	0.66 (0.24-1.83)	69.2	
JAK inhibitor type						
Baricitinib	4	4.31 (2.89-6.41)	3.18 (2.43-4.16)	1.06 (0.86-1.31)	0	.92
Tofacitinib	12	9.92 (2.95-5.21)	3.3 (2.52-4.29)	1.09 (0.99-1.21)	1.2	
Upadacitinib	2	5.2 (4.09-6.6)	4.47 (3.14-6.37)	1.10 (0.51-2.33)	0	
**Malignant neoplasms**						
Geographic location						
Asia	8	9.67 (0.39-1.14)	0.63 (0.4-0.98)	0.98 (0.70-1.39)	26.8	.75
North America	5	1.21 (0.73-2.03)	1.12 (0.71-1.75)	1.11 (0.78-1.56)	49.9	
Europe	6	1.14 (0.76-1.72)	1.09 (0.72-1.64)	0.99 (0.79-1.25)	0	
Malignant neoplasm subtype						
Solid organ	5	0.2 (0.16-0.27)	0.22 (0.19-0.24)	0.93 (0.70-1.25)	0	.5
Hematologic	5	0.2 (0.12-0.32)	0.23 (0.2-0.27)	1.10 (0.59-2.04)	0	
IMID type						
Rheumatoid arthritis	17	1.0 (0.76-1.33)	0.99 (0.75-1.29)	1.05 (0.91-1.20)	21.1	.71
Spondyloarthritis	1	1.06 (0.4-2.84)	1.34 (1.13-1.58)	0.90 (0.30-2.50)	NA	
Psoriatic arthritis	1	0.73 (0.11-1.61)	0.58 (0.44-0.71)	1.88 (0.68-5.16)	NA	
IBD	1	0.18 (00-0.53)	0.19 (0.02-0.37)	0.98 (0.11-8.42)	NA	
JAK inhibitor type						
Baricitinib	3	0.65 (0.34-1.25)	0.63 (0.31-1.3)	0.95 (0.16-5.68)	0	.82
Tofacitinib	9	0.96 (0.63-1.45)	0.91 (0.6-1.37)	1.07 (0.86-1.34)	12.5	
Upadacitinib	1	0.35 (0-0.76)	0.37 (0.29-0.44)	1.84 (0.35-9.73)	NA	
Age group, y						
<65	4	1.07 (0.79-1.45)	0.94 (0.85-1.04)	0.85 (0.41-1.74)	56.8	.38
≥65	5	2.64 (2.14-3.26)	2.49 (2.31-2.68)	1.05 (0.82-1.34)	0	
**Major adverse cardiovascular events**						
Geographic location						
Asia	11	0.64 (0.38-1.09)	0.57 (0.31-1.03)	0.97 (0.80-1.19)	14.5	.67
North America	10	0.71 (0.45-1.12)	0.66 (0.42-1.02)	0.70 (0.31-1.59)	76.3	
Europe	7	0.69 (0.53-0.92)	0.65 (0.49-0.86)	0.91 (0.69-1.19)	30.4	
Cardiovascular event subtype						
Myocardial infarction	11	0.68 (0.59-0.77)	0.91 (0.87-0.96)	0.86 (0.69-1.09)	37.6	.89
Stroke	11	0.41 (0.35-0.49)	1.03 (1.005-1.06)	0.85 (0.72-1.00)	0	
IMID type						
Rheumatoid arthritis	25	0.69 (0.52-0.91)	0.61 (0.45-0.83)	0.92 (0.79-1.06)	53	.88
IBD	2	0.51 (0.09-2.84)	0.83 (0.57-1.19)	0.89 (0-26718.38)	70.8	
JAK inhibitor type						
Baricitinib	6	0.68 (0.51-0.91)	0.57 (0.39-0.82)	0.93 (0.65-1.32)	39.7	<.001
Tofacitinib	14	0.62 (0.42-0.91)	0.62 (0.45-0.85)	0.84 (0.58-1.22)	62.2	
Upadacitinib	2	0.11 (0.01-1.29)	0.48 (0.24-0.95)	0.48 (0.37-0.62)	0	
Age group, y						
<65	7	0.53 (0.41-0.69)	0.57 (0.51-0.63)	0.90 (0.76-1.08)	0	.45
≥65	8	1.73 (1.45-2.05)	1.71 (1.59-1.85)	0.98 (0.82-1.16)	0	
**Venous thromboembolism**						
Geographic location						
Asia	6	0.38 (0.22-0.63)	0.34 (0.31-0.37)	1.28 (0.53-3.13)	49.5	.21
North America	7	0.56 (0.28-1.11)	0.57 (0.35-0.81)	1.06 (0.72-1.55)	2.2	
Europe	4	0.72 (0.47-1.1)	0.69 (0.23-2.02)	1.46 (1.03-2.50)	0	
Venous thromboembolic event subtype						
Deep venous thrombosis	8	2.16 (1.79-2.6)	1.37 (1.29-1.45)	1.09 (0.75-1.59)	58.9	.22
Pulmonary embolism	11	0.53 (0.35-0.79)	0.32 (0.28-0.36)	1.44 (1.00-2.07)	62.3	
IMID type						
Rheumatoid arthritis	14	0.49 (0.35-0.69)	0.46 (0.32-0.66)	1.31 (1.07-1.60)	30.4	.007
IBD	2	1.32 (0.76-2.3)	1.09 (0.99-1.21)	0.70 (0.05-10.49)	0	
JAK inhibitor type						
Baricitinib	3	0.84 (0.57-1.23)	0.98 (0.32-3.04)	1.53 (0.99-2.37)	0	.03
Tofacitinib	10	0.52 (0.32-0.85)	0.67 (0.41-1.09)	1.08 (0.84-1.38)	0	
Upadacitinib	1	1.32 (0.00-2.81)	1.22 (1.05-1.39)	0.64 (0.20-2.08)	8.4	
Age group, y						
<60	1	NA	NA	2.11 (1.09-4.07)	0	.25
≥60	6	NA	NA	1.42 (1.14-1.77)	0	

Abbreviations: HR, hazard ratio; IBD, inflammatory bowel disease; IMID, immune-mediated inflammatory disease; IR, incidence rate; JAK, Janus kinase; NA, not applicable; TNF, tumor necrosis factor.

### Risk of Malignant Neoplasms

**Figure 2.**
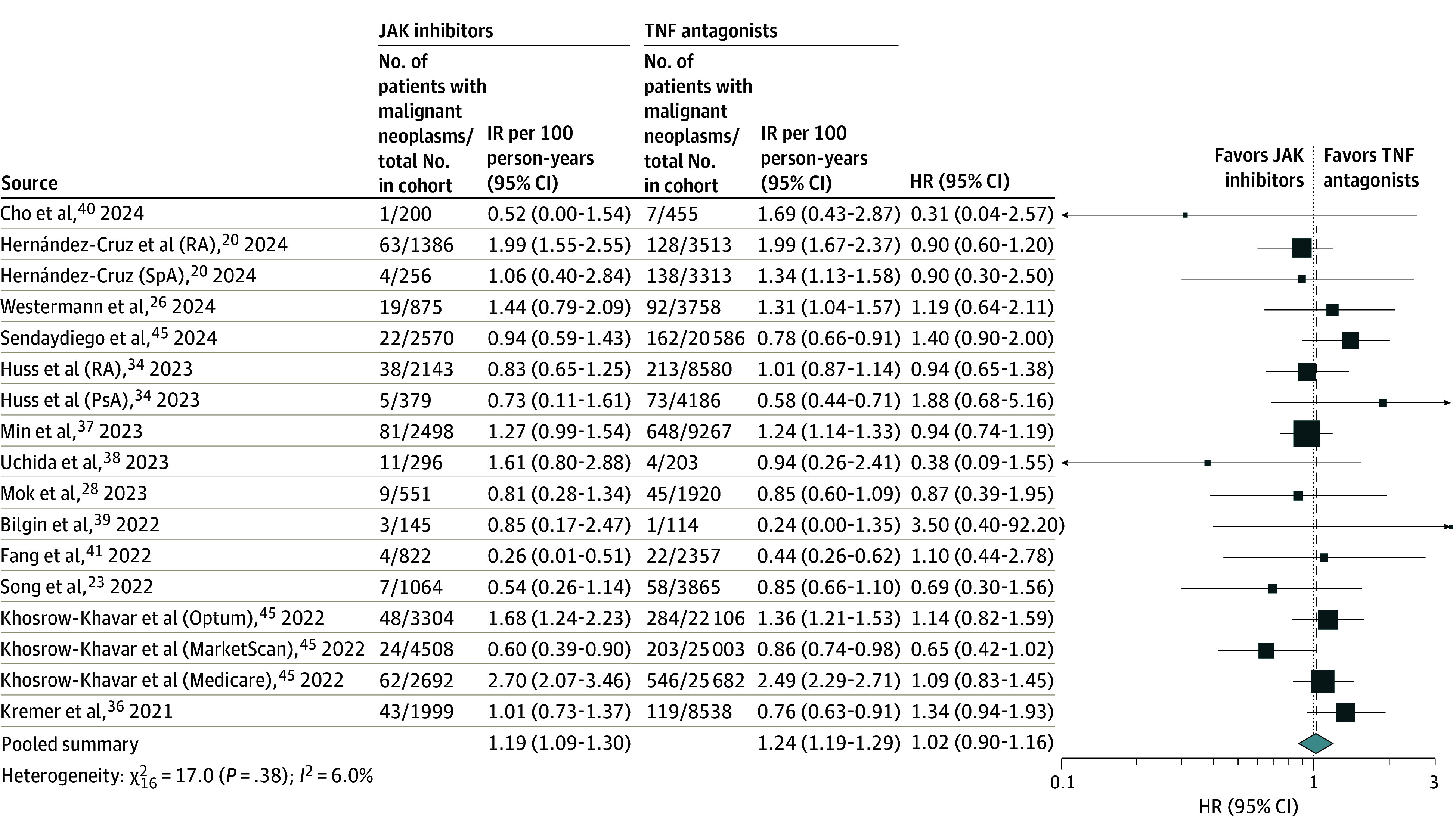
Risk of Malignant Neoplasms With Janus Kinase (JAK) Inhibitors vs Tumor Necrosis Factor (TNF) Antagonists in All Patients With Immune-Mediated Inflammatory Diseases Box represents the effect size estimate from the individual study included in the meta-analysis. The size of the box corresponds to the weight of that study's estimate, with larger boxes indicating more weight or precision. Horizontal lines extending from the box represent the CI for that study's effect. HR indicates hazard ratio; IR, incidence rate; PsA, psoriatic arthritis; RA, rheumatoid arthritis; SpA, spondyloarthropathy.

The meta-analysis included 17 cohorts involving a total of 185 530 patients with IMIDs (33 681 treated with JAK inhibitors vs 151 849 treated with TNF antagonists), with 3467 case patients with malignant neoplasms.^20,23,26,28,34,36,37,38,39,40,41,42,45^The IR of malignant neoplasms was 1.00 (95% CI, 0.77-1.31) per 100 person-years with JAK inhibitors vs 0.94 (95% CI, 0.72-1.22) per 100 person-years with TNF antagonists. There was no significant difference in the risk of malignant neoplasms between JAK inhibitors vs TNF antagonists (pooled HR, 1.02 [95% CI, 0.90-1.16]), with low heterogeneity (*I*^2^ = 6.0%) (Figure 2).

Overall findings remained stable on subgroup analyses based on geographic location, IMID type, JAK inhibitor type, age group, and malignant neoplasm type (solid organ vs hematologic) (Table and eFigures 6-10 in Supplement 1) and on sensitivity analysis excluding studies without adjustment for confounding. On meta-regression, mean age, sex distribution, midpoint of study recruitment, concomitant methotrexate or corticosteroid use (or both), prevalence of major comorbidities (eg, diabetes, hypertension, hyperlipidemia, prior cardiovascular disease, or obesity), and smoking did not alter the association (eTable 4 in Supplement 1). We did not observe any evidence of publication bias (Egger regression coefficient, *P* = .63) (eFigure 11 in Supplement 1).

### Risk of MACEs

**Figure 3.**
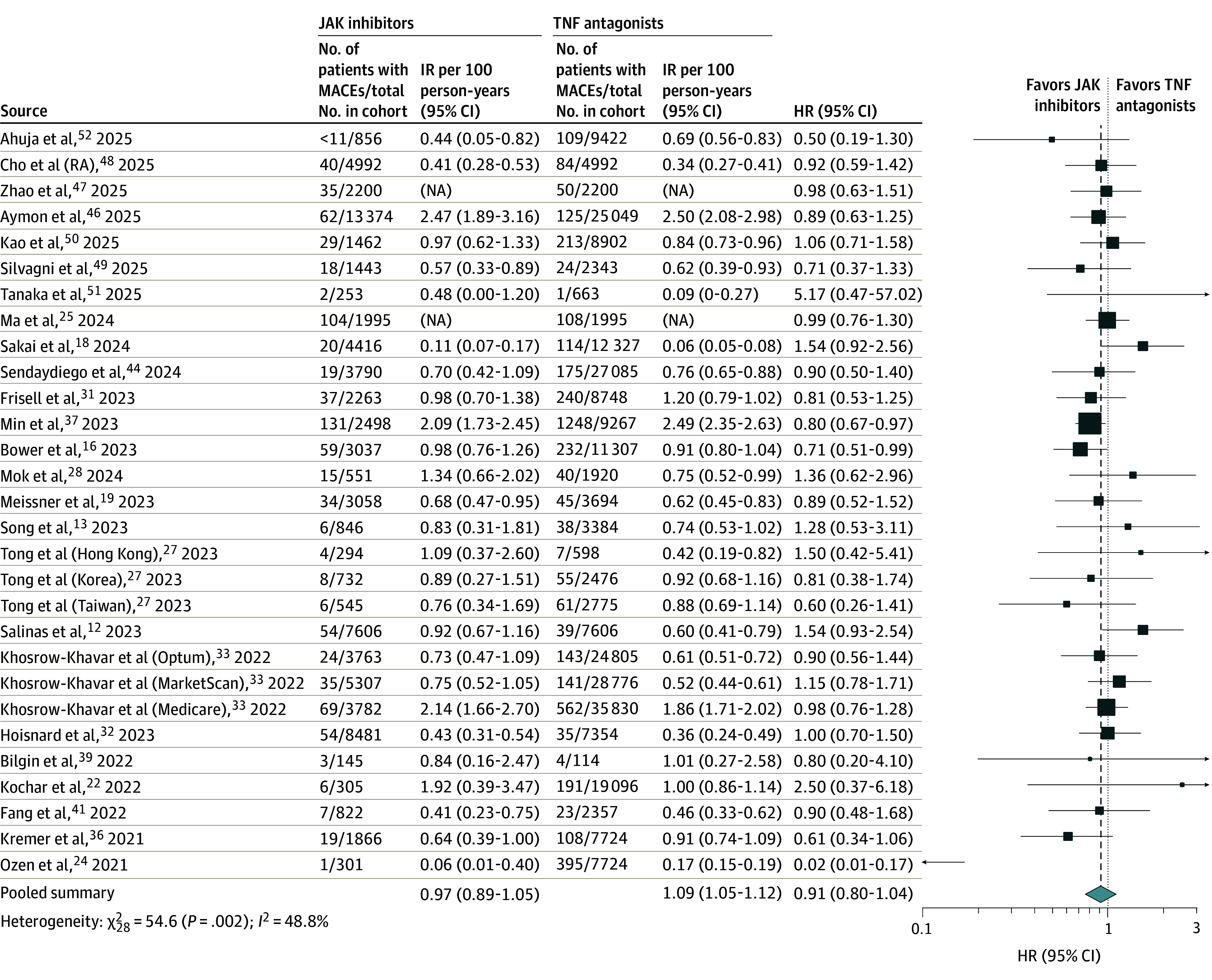
Risk of Major Adverse Cardiovascular Events (MACEs) With Janus Kinase (JAK) Inhibitors vs Tumor Necrosis Factor (TNF) Antagonists in All Patients With Immune-Mediated Inflammatory Diseases Box represents the effect size estimate from the individual study included in the meta-analysis. The size of the box corresponds to the weight of that study's estimate, with larger boxes indicating more weight or precision. Horizontal lines extending from the box represent the CI for that study's effect. HR indicates hazard ratio; IR, incidence rate; NA, not applicable; RA, rheumatoid arthritis.

The meta-analysis included 29 cohorts involving a total of 361 516 patients with IMIDs (80 983 treated with JAK inhibitors vs 280 533 treated with TNF antagonists), with 5522 case patients with MACEs.^12,13,16,18,19,22,24,25,27,28,31,32,33,36,37,39,41,44,46,47,48,49,50,51,52^ The IR of MACEs was 0.72 (95% CI, 0.56-0.92) per 100 person-years with JAK inhibitors vs 0.66 (95% CI, 0.49-0.89) per 100 person-years with TNF antagonists. There was no significant difference in the risk of MACEs between JAK inhibitors vs TNF antagonists (pooled HR, 0.91 [95% CI, 0.80-1.04]), with moderate heterogeneity (*I*^2^ = 48.8%) (Figure 3).

Overall findings remained stable on subgroup analyses based on geographic location, IMID type, age group, and MACE type (Table and eFigures 12-16 in Supplement 1) and on sensitivity analysis excluding studies without adjustment for confounding. On subgroup analysis by JAK inhibitor type, the magnitude of increased risk of MACEs was lower with upadacitinib vs TNF antagonists (HR, 0.48 [95% CI, 0.37-0.62]; *P* < .001 for subgroup differences). On meta-regression, obesity was associated with increased risk of MACEs with JAK inhibitors vs TNF antagonists (eTable 4 in Supplement 1). Mean age, sex distribution, midpoint of recruitment, concomitant immunomodulator or corticosteroid use (or both), and prevalence of other major comorbidities (including diabetes, smoking status, hypertension, hyperlipidemia, or prior coronary artery disease) did not alter the association. There was no evidence of publication bias (Egger regression coefficient, *P* = .94) (eFigure 17 in Supplement 1).

### Risk of VTE

**Figure 4.**
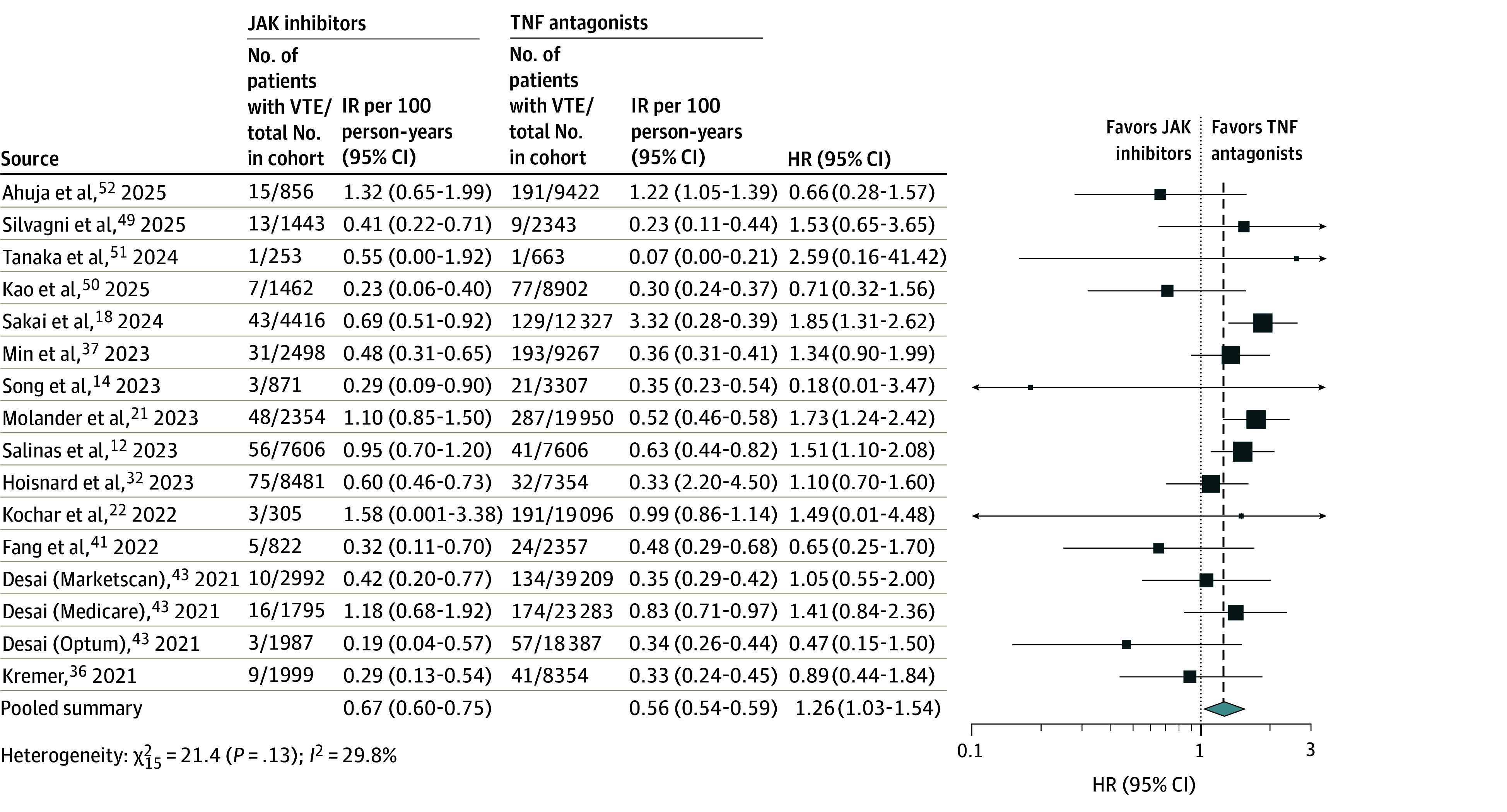
Risk of Venous Thromboembolism (VTE) With Janus Kinase (JAK) Inhibitors vs Tumor Necrosis Factor (TNF) Antagonists in All Patients With Immune-Mediated Inflammatory Diseases Box represents the effect size estimate from the individual study included in the meta-analysis. The size of the box corresponds to the weight of that study's estimate, with larger boxes indicating more weight or precision. Horizontal lines extending from the box represent the CI for that study's effect. HR indicates hazard ratio; IR, incidence rate.

The meta-analysis included 16 cohorts involving a total of 231 967 patients with IMID (40 140 treated with JAK inhibitors vs 191 827 treated with TNF antagonists), with 1940 case patients with VTE.^12,14,18,21,22,32,36,37,41,43,49,50,51,52^ The IR of VTE was 0.57 (95% CI, 0.40-0.82) per 100 person-years with JAK inhibitors vs 0.52 (95% CI, 0.37-0.73) per 100 person-years with TNF antagonists. There was a significantly higher risk of VTE with JAK inhibitors compared with TNF antagonists (pooled HR, 1.26 [95% CI, 1.03-1.54]), with moderate heterogeneity (*I*^2^ = 29.8%) (Figure 4).

Overall findings remained stable on subgroup analyses based on geographic location, age group, and VTE type (Table and eFigures 18-21 in Supplement 1). However, there was a significant difference in the magnitude of risk based on IMID type comparing RA vs IBD (*P* = .03 for difference between subgroups). In patients with RA, JAK inhibitors were associated with a 31.0% higher risk of VTE compared with TNF antagonists (HR, 1.31 [95% CI, 1.07-1.60]), with moderate heterogeneity; in contrast, in patients with IBD, there was no significant difference in the risk of VTE (HR, 0.70 [95% CI, 0.05-10.49]). On subgroup analysis by JAK inhibitor type, the magnitude of increased risk of VTE was higher with baricitinib vs TNF antagonists (n = 3 cohorts; HR, 1.53 [95% CI, 0.99-2.37]), compared with tofacitinib vs TNF antagonists (n = 10 cohorts; HR, 1.08 [95% CI, 0.84-1.38]) and upadacitinib vs TNF antagonists (n = 1 cohort; HR, 0.64 [95% CI, 0.20-2.08]) (*P* = .03 for difference between subgroups) (Table and eFigure 22 in Supplement 1). On meta-regression, obesity and smoking were associated with increased risk of VTE with JAK inhibitors vs TNF antagonists (eTable 4 in Supplement 1). Mean age, sex distribution, midpoint of recruitment, concomitant immunomodulator or corticosteroid use (or both), and prevalence of other major comorbidities (including diabetes, hypertension, hyperlipidemia, or prior coronary artery disease) did not alter the association. We observed publication bias in this analysis (Egger regression coefficient, *P* = .03) (eFigure 23 in Supplement 1).

## Discussion

Since regulatory concerns on the safety of JAK inhibitors were raised in 2019, leading to a label change in the US, there has been intense debate on the comparative safety of JAK inhibitors vs TNF antagonists in patients with IMIDs. Through a comprehensive systematic review and meta-analysis of 42 head-to-head comparative effectiveness studies involving a total of 813 881 patients with IMIDs treated with JAK inhibitors or TNF antagonists, we made several key observations on the safety of these agents, as described next.

First, we observed that there was no significant difference in the risk of serious infections between JAK inhibitors vs TNF antagonists, with an overall IR of approximately 3.79 vs 3.03 per 100 person-years. Second, we observed no significant difference in the risk of malignant neoplasms with JAK inhibitors vs TNF antagonists, after excluding nonmelanoma skin cancers, with an overall incidence of 1.00 vs 0.94 per 100 person-years. Third, we observed no significant difference in the risk of MACEs or VTE with JAK inhibitors vs TNF antagonists, with an overall low incidence of 0.72 vs 0.66 or 0.57 vs 0.52 per 100 person-years, respectively. However, in patients with RA, there was a modest 31.0% higher risk of VTE with JAK inhibitors vs TNF antagonists, which was higher than the risk observed in patients with IBD. The magnitude of increased risk was also higher with baricitinib (vs TNF antagonists) compared with tofacitinib or upadacitinib (vs TNF antagonists). Overall, our findings from head-to-head comparative effectiveness studies support the safety of JAK inhibitors compared with TNF antagonists. In contrast with the ORAL Surveillance trial, which was restricted to older patients with RA and cardiovascular risk factors treated with low and high doses of tofacitinib, our findings are more generalizable across age groups, different IMIDs, and different types of JAK inhibitors and inform decision-making in clinical practice. These findings also call for revisiting the strict regulatory guidance imposed by the US Food and Drug Administration restricting the use of all JAK inhibitors after failure of, or contraindications to, TNF antagonists, across all indications.

Several factors may explain why our observations from head-to-head comparative effectiveness studies are distinct from the findings of the ORAL Surveillance trial. First, we included patients with all IMIDs, across all age groups, treated with all JAK inhibitors and did not restrict our analysis to patients with increased risk of cardiovascular events. This approach makes our findings more generalizable. Second, it is possible that with evolving regulatory guidance, clinical utilization of JAK inhibitors evolved with careful selection of patients prescribed JAK inhibitors, avoiding their use in patients with higher risk of MACEs and VTE. Although our analyses accounted for risk factors associated with MACEs and VTE, both at an individual study level through PS methods and at an analysis level through subgroup analyses and meta-regression, it is not possible to fully account for unmeasured confounders. Third, whereas the ORAL Surveillance trial was restricted to patients naïve to TNF antagonist and JAK inhibitor treatment, we did not impose any restrictions on prior exposure to advanced therapies. Recent studies suggest that prior exposure to TNF antagonists might potentiate the effectiveness of JAK inhibitors.^53,54^ Including patients with prior TNF antagonist exposure in the analysis may modify the risk of disease-mediated adverse events, such as serious infections, MACEs, and VTE, in which high disease activity and ability to control the disease with effective therapies might modify the association between JAK inhibitors and TNF antagonists as discussed next. Moreover, most patients in our cohort were treated with lower doses of tofacitinib, whereas higher doses of tofacitinib were associated with higher risk of MACEs and VTE in the ORAL Surveillance trial. Fourth, although outcomes were carefully adjudicated in the ORAL Surveillance trial, we relied on administrative claims codes or medical record review for registry studies for outcome ascertainment. Although the former is more rigorous, the definitions used for outcome ascertainment in the included comparative effectiveness studies have been validated and generally have high positive predictive value. Finally, the duration of follow-up was longer in the ORAL Surveillance trial, with a median follow-up of approximately 4 years compared with 14 to 35 months in the studies included in this meta-analysis.

Although safety is a critical factor in clinical decision-making, it should be interpreted in the context of medication effectiveness. Several safety outcomes, such as serious infections, MACEs, and VTE, may be driven by both immune-suppressing and prothrombotic potential of medications, as well as effectiveness of the treatment strategy in controlling disease activity. For example, JAK inhibitors may be more effective than TNF antagonists in controlling disease in patients with ulcerative colitis, leading to lower rates of corticosteroid use.^55,56^ This finding may explain the lower risk of serious infections with JAK inhibitors vs TNF antagonists in patients with IBD. In contrast, for RA where the effectiveness of JAK inhibitors may be comparable to TNF antagonists, we observed a modestly higher risk of VTE with JAK inhibitors, likely related to an intrinsic prothrombotic potential of JAK inhibitors particularly at higher doses.

We accounted for all JAK inhibitors in our analysis, providing an opportunity to examine relative differences in safety profiles of different agents. Although we did not observe any significant difference in the magnitude of risk of adverse events with tofacitinib or JAK1 inhibitors vs TNF antagonists for most outcomes, we observed a higher risk of VTE with baricitinib vs TNF antagonists, but not with tofacitinib vs TNF antagonists. This finding should be interpreted with caution due to the limited number of patients treated with baricitinib across all studies. Differing selectivity profiles of JAK inhibitors may potentially modify the safety profile of different agents. Baricitinib is a more selective JAK1 and JAK2 inhibitor, whereas tofacitinib is a pan-JAK inhibitor, acting on JAK1, JAK2, JAK3, and TYK2. Further data on selective JAK1 inhibitors such as upadacitinib and filgotinib are essential to better understand the full scope of safety outcomes as well as the association between JAK selectivity and risk of thromboembolic events and other adverse events.^57^

### Limitations

There are important limitations to our analysis. First, as a meta-analysis focusing on head-to-head comparative effectiveness studies, it is difficult to draw causal inference due to absence of randomization and limited patient-level data for individual studies. Several factors may drive preferential use of JAK inhibitors vs TNF antagonists in clinical practice that could not be fully accounted for despite attempts at balancing groups through propensity scores. These outcomes are confounded by several factors and not just the medication received. Although most studies reported HRs adjusted for confounders, the approach to this analysis was not consistent, and pooling of HRs across variable studies cannot ensure satisfactory coverage of impact of known confounders either. Second, although most analyses had minimal to moderate heterogeneity, we were unable to fully account for observed heterogeneity despite a priori subgroup analyses and study-level meta-regression. Third, the median duration of follow-up was less than 2 years across the included studies. It may be hard to infer long-term comparative safety of JAK inhibitors vs TNF antagonists, particularly for malignant neoplasms. Fourth, the patients had diversity not only in the type of IMIDs but also the characteristics independently influencing the risk of those outcomes. Due to lack of patient-level data, it is hard to precisely attribute the relative contribution of these outcomes due to medications alone. Fifth, the outcome definitions are not standardized across all studies, and the criteria for reporting the outcome may vary across studies. Additionally, not all adverse event categories of interest were consistently reported across studies; this likely reflects selective reporting based on study objectives or data availability and may introduce reporting bias, limiting comparability across outcomes. To reduce this risk, we focused on adverse events that were most consistently reported and aligned with regulatory safety concerns and the ORAL Surveillance trial. There was also variability in the reported metric of association. We preferentially used HRs adjusted for key confounding variables, but if that was not reported, we relied on IRRs. Finally, although emerging evidence suggests potential differences in safety profiles between individual TNF antagonists (eg, adalimumab and etanercept), this level of granularity was not consistently reported across studies. As such, we were unable to explore whether specific TNF antagonist comparators factored into the magnitude of observed risks.

## Conclusions

In this systematic review and meta-analysis of head-to-head comparative effectiveness studies involving patients with IMIDs treated with JAK inhibitors or TNF antagonists, we observed no meaningful differences in the risk of serious infections, malignant neoplasms, MACEs, or VTE, with a low incidence of serious adverse events. These findings support JAK inhibitor use with appropriate patient selection, monitoring, and strategies to optimize disease control. Further research, especially long-term studies, is needed to fully elucidate the safety of these agents across diverse populations and optimize clinical use.

## Notes

Supplement 1. **eAppendix.** Search Strategy
eMethods
**eTable 1.** Characteristics of Included Studies **eTable 2.** Characteristics of Patients in Included Studies **eTable 3.** Risk of Bias in the Studies Included in the Systematic Review According to the Newcastle-Ottawa Scale **eTable 4.** Meta-Regression Analyses for All Outcomes of Interest **eFigure 1.** PRISMA Flow Diagram **eFigure 2.** Risk of Serious Infections With JAK Inhibitors vs TNF Antagonists in All Patients With Immune-Mediated Inflammatory Diseases (Subgroup Analysis Based on Location of Study) **eFigure 3.** Risk of Serious Infections With JAK inhibitors vs TNF Antagonists in All Patients With Immune-Mediated Inflammatory Diseases (IMIDs) (Subgroup Analysis Based on Type of IMID) **eFigure 4.** Risk of Serious Infections With JAK Inhibitors vs TNF Antagonists in All Patients With Immune-Mediated Inflammatory Diseases (Subgroup Analysis Based on Type of JAK Inhibitor) **eFigure 5.** Funnel Plots for Publication Bias for Serious Infection Outcome **eFigure 6.** Risk of Malignant Neoplasms With JAK Inhibitors vs TNF Antagonists in All Patients With Immune-Mediated Inflammatory Diseases (Subgroup Analysis Based on Location of Study) **eFigure 7.** Risk of Malignant Neoplasms With JAK Inhibitors vs TNF Antagonists in All Patients With Immune-Mediated Inflammatory Diseases (Subgroup Analysis Based on Subtype of Malignant Neoplasm) **eFigure 8.** Risk of Malignant Neoplasms With JAK Inhibitors vs TNF Antagonists in All Patients With Immune-Mediated Inflammatory Diseases (Subgroup Analysis Based on Type of JAK Inhibitor) **eFigure 9.** Risk of Malignant Neoplasms With JAK Inhibitors vs TNF Antagonists in All Patients With Immune-Mediated Inflammatory Diseases (IMIDs) (Subgroup Analysis Based on Type of IMID) **eFigure 10.** Risk of Malignant Neoplasms With JAK Inhibitors vs TNF Antagonists in All Patients With Immune-Mediated Inflammatory Diseases (Subgroup Analysis Based on Age Group) **eFigure 11.** Funnel Plots for Publication Bias for Malignant Neoplasm Outcome **eFigure 12.** Risk of Major Cardiovascular Events With JAK Inhibitors vs TNF Antagonists in All Patients With Immune-Mediated Inflammatory Diseases (Subgroup Analysis Based on Location of Study) **eFigure 13.** Risk of Major Cardiovascular Events (MACEs) With JAK Inhibitors vs TNF Antagonists in All Patients With Immune-Mediated Inflammatory Diseases (Subgroup Analysis Based on Subtype of MACE) **eFigure 14.** Risk of Major Cardiovascular Events With JAK Inhibitors vs TNF Antagonists in All Patients With Immune-Mediated Inflammatory Diseases (Subgroup Analysis Based on Type of JAK Inhibitor) **eFigure 15.** Risk of Major Cardiovascular Events With JAK Inhibitors vs TNF Antagonists in All Patients With Immune-Mediated Inflammatory Diseases (Subgroup Analysis Based on Type of IMID) **eFigure 16.** Risk of Major Cardiovascular Events With JAK inhibitors vs TNF Antagonists in All Patients With Immune-Mediated Inflammatory Diseases (Subgroup Analysis Based on Age Group) **eFigure 17.** Funnel Plots for Publication Bias for Major Cardiovascular Event Outcome **eFigure 18.** Risk of Venous Thromboembolism With JAK Inhibitors vs TNF Antagonists in All Patients With Immune-Mediated Inflammatory Diseases (Subgroup Analysis Based on Location of Study) **eFigure 19.** Risk of Venous Thromboembolism (VTE) With JAK Inhibitors vs TNF Antagonists in All Patients With Immune-Mediated Inflammatory Diseases (Subgroup Analysis Based on Subtype of VTE) **eFigure 20.** Risk of Venous Thromboembolism With JAK inhibitors vs TNF Antagonists in All Patients With Immune-Mediated Inflammatory Diseases (Subgroup Analysis Based on Type of JAK Inhibitors) **eFigure 21.** Risk of Venous Thromboembolism With JAK inhibitors vs TNF Antagonists in All Patients With Immune-Mediated Inflammatory Diseases (IMIDs) (Subgroup Analysis Based on Type of IMID) **eFigure 22.** Risk of Venous Thromboembolism With JAK Inhibitors vs TNF Antagonists in All Patients With Immune-Mediated Inflammatory Diseases (Subgroup Analysis Based on Age Group) **eFigure 23.** Funnel Plots for Publication Bias for Venous Thromboembolism Outcome
eReferences

Supplement 2.
Data Sharing Statement
