# Supplementary material for: Comparative Safety of JAK Inhibitors vs TNF Antagonists in Immune-Mediated Inflammatory Diseases: A Systematic Review and Meta-Analysis
Source: JAMA Netw Open. 2025 Sep 10;8(9):e2531204. doi: 10.1001/jamanetworkopen.2025.31204 (PMC12423869; doi:10.1001/jamanetworkopen.2025.31204)
Supplement: Supplement 1. — eAppendix. Search Strategy eMethods eTable 1. Characteristics of Included Studies eTable 2. Characteristics of Patients in Included Studies eTable 3. Risk of Bias in the Studies Included in the Systematic Review According to the Newcastle-Ottawa Scale eTable 4. Meta-Regression Analyses for All Outcomes of Interest eFigure 1. PRISMA Flow Diagram eFigure 2. Risk of Serious Infections With JAK Inhibitors vs TNF Antagonists in All Patients With Immune-Mediated Inflammatory Diseases (Subgroup Analysis Based on Location of Study) eFigure 3. Risk of Serious Infections With JAK inhibitors vs TNF Antagonists in All Patients With Immune-Mediated Inflammatory Diseases (IMIDs) (Subgroup Analysis Based on Type of IMID) eFigure 4. Risk of Serious Infections With JAK Inhibitors vs TNF Antagonists in All Patients With Immune-Mediated Inflammatory Diseases (Subgroup Analysis Based on Type of JAK Inhibitor) eFigure 5. Funnel Plots for Publication Bias for Serious Infection Outcome eFigure 6. Risk of Malignant Neoplasms With JAK Inhibitors vs TNF Antagonists in All Patients With Immune-Mediated Inflammatory Diseases (Subgroup Analysis Based on Location of Study) eFigure 7. Risk of Malignant Neoplasms With JAK Inhibitors vs TNF Antagonists in All Patients With Immune-Mediated Inflammatory Diseases (Subgroup Analysis Based on Subtype of Malignant Neoplasm) eFigure 8. Risk of Malignant Neoplasms With JAK Inhibitors vs TNF Antagonists in All Patients With Immune-Mediated Inflammatory Diseases (Subgroup Analysis Based on Type of JAK Inhibitor) eFigure 9. Risk of Malignant Neoplasms With JAK Inhibitors vs TNF Antagonists in All Patients With Immune-Mediated Inflammatory Diseases (IMIDs) (Subgroup Analysis Based on Type of IMID) eFigure 10. Risk of Malignant Neoplasms With JAK Inhibitors vs TNF Antagonists in All Patients With Immune-Mediated Inflammatory Diseases (Subgroup Analysis Based on Age Group) eFigure 11. Funnel Plots for Publication Bias for Malignant Neoplasm Outcome eFigure 12. R [file jamanetwopen-e2531204-s001.pdf]

Supplemental Online Content

Solitano V, Ahuja D, Lee HH, et al. Comparative safety of JAK inhibitors vs TNF antagonists in immune-mediated inflammatory diseases: a systematic review and meta-analysis. *JAMA Netw Open*. 2025;8(7):e2522211. doi:10.1001/jamanetworkopen.2025.22211

eAppendix. Search Strategy

eMethods

eTable 1. Characteristics of Included Studies

eTable 2. Characteristics of Patients in Included Studies

eTable 3. Risk of Bias in the Studies Included in the Systematic Review According to the Newcastle-Ottawa Scale

eTable 4. Meta-Regression Analyses for All Outcomes of Interest

eFigure 1. PRISMA Flow Diagram

eFigure 2. Risk of Serious Infections With JAK Inhibitors vs TNF Antagonists in All Patients With Immune-Mediated Inflammatory Diseases (Subgroup Analysis Based on Location of Study)

eFigure 3. Risk of Serious Infections With JAK inhibitors vs TNF Antagonists in All Patients With Immune-Mediated Inflammatory Diseases (IMIDs) (Subgroup Analysis Based on Type of IMID)

eFigure 4. Risk of Serious Infections With JAK Inhibitors vs TNF Antagonists in All Patients With Immune-Mediated Inflammatory Diseases (Subgroup Analysis Based on Type of JAK Inhibitor)

eFigure 5. Funnel Plots for Publication Bias for Serious Infection Outcome

eFigure 6. Risk of Malignant Neoplasms With JAK Inhibitors vs TNF Antagonists in All Patients With Immune-Mediated Inflammatory Diseases (Subgroup Analysis Based on Location of Study)

eFigure 7. Risk of Malignant Neoplasms With JAK Inhibitors vs TNF Antagonists in All Patients With Immune-Mediated Inflammatory Diseases (Subgroup Analysis Based on Subtype of Malignant Neoplasm)

eFigure 8. Risk of Malignant Neoplasms With JAK Inhibitors vs TNF Antagonists in All Patients With Immune-Mediated Inflammatory Diseases (Subgroup Analysis Based on Type of JAK Inhibitor)

eFigure 9. Risk of Malignant Neoplasms With JAK Inhibitors vs TNF Antagonists in All Patients With Immune-Mediated Inflammatory Diseases (IMIDs) (Subgroup Analysis Based on Type of IMID)

eFigure 10. Risk of Malignant Neoplasms With JAK Inhibitors vs TNF Antagonists in All Patients With Immune-Mediated Inflammatory Diseases (Subgroup Analysis Based on Age Group)

eFigure 11. Funnel Plots for Publication Bias for Malignant Neoplasm Outcome

© 2025 Solitano V et al. *JAMA Network Open*.

eFigure 12. Risk of Major Cardiovascular Events With JAK Inhibitors vs TNF Antagonists in All Patients With Immune-Mediated Inflammatory Diseases (Subgroup Analysis Based on Location of Study)

eFigure 13. Risk of Major Cardiovascular Events (MACEs) With JAK Inhibitors vs TNF Antagonists in All Patients With Immune-Mediated Inflammatory Diseases (Subgroup Analysis Based on Subtype of MACE)

eFigure 14. Risk of Major Cardiovascular Events With JAK Inhibitors vs TNF Antagonists in All Patients With Immune-Mediated Inflammatory Diseases (Subgroup Analysis Based on Type of JAK Inhibitor)

eFigure 15. Risk of Major Cardiovascular Events With JAK Inhibitors vs TNF Antagonists in All Patients With Immune-Mediated Inflammatory Diseases (Subgroup Analysis Based on Type of IMID)

eFigure 16. Risk of Major Cardiovascular Events With JAK inhibitors vs TNF Antagonists in All Patients With Immune-Mediated Inflammatory Diseases (Subgroup Analysis Based on Age Group)

eFigure 17. Funnel Plots for Publication Bias for Major Cardiovascular Event Outcome

eFigure 18. Risk of Venous Thromboembolism With JAK Inhibitors vs TNF Antagonists in All Patients With Immune-Mediated Inflammatory Diseases (Subgroup Analysis Based on Location of Study)

eFigure 19. Risk of Venous Thromboembolism (VTE) With JAK Inhibitors vs TNF Antagonists in All Patients With Immune-Mediated Inflammatory Diseases (Subgroup Analysis Based on Subtype of VTE)

eFigure 20. Risk of Venous Thromboembolism With JAK inhibitors vs TNF Antagonists in All Patients With Immune-Mediated Inflammatory Diseases (Subgroup Analysis Based on Type of JAK Inhibitors)

eFigure 21. Risk of Venous Thromboembolism With JAK inhibitors vs TNF Antagonists in All Patients With Immune-Mediated Inflammatory Diseases (IMIDs) (Subgroup Analysis Based on Type of IMID)

eFigure 22. Risk of Venous Thromboembolism With JAK Inhibitors vs TNF Antagonists in All Patients With Immune-Mediated Inflammatory Diseases (Subgroup Analysis Based on Age Group)

eFigure 23. Funnel Plots for Publication Bias for Venous Thromboembolism Outcome

eReferences

This supplemental material has been provided by the authors to give readers additional information about their work.

eAppendix. Search Strategy

Database (via Ovid): Embase <1974 to 2024 July 10>, EBM Reviews - Cochrane Central Register of Controlled Trials <June 2024>, Ovid MEDLINE(R) ALL <1946 to July 10, 2024>

1. exp Inflammatory Bowel Diseases/ use medall,cctr
2. exp inflammatory bowel disease/ use oemezd
3. (ulcerative colitis or idiopathic proctocolitis or colitis Gravis).ti,ab,kw.
4. (crohn* or regional colitis or regional enteritis or granulomatous enteritis or granulomatous colitis).ti,ab,kw.
5. (IBD or inflammatory bowel disease*).ti,ab,kw.
6. or/1-5 [IBD]
7. exp Arthritis, Rheumatoid/ use medall,cctr
8. exp rheumatoid arthritis/ use oemezd
9. (arthritis or rheumarthritis).ti,ab,kw.
10. (rheumat* adj3 (articular or joints or nodule* or periarthritis)).ti,ab,kw.
11. or/7-10 [RA]
12. exp Psoriasis/ use medall,cctr,oemezd
13. (psoriasis or psoriatic or Psoriases or Palmoplantaris Pustulosis or psoriasiform spondyloarthropathy).ti,ab,kw.
14. (IMIDs or (Immune-mediated adj2 (disease* or disorder*))).ti,ab,kw.
15. or/12-14 [PA or IMIDs]
16. 6 or 11 or 15 [IMIDs]
17. Janus Kinase Inhibitors/ use medall,cctr
18. exp Janus kinase inhibitor/ use oemezd
19. ((Janus Kinase or Janus tyrosine kinase or JAK) adj3 Inhibit*).ti,ab,kw.
20. (tofacitinib or cgb 500 or cgb500 or cp 690 550 or cp 690550 or jaquinus or pgn 600 or pgn600 or prd 4862257 or prd4862257 or ro 5169503 or ro5169503 or Xeljanz).ti,ab,kw.
21. (upadacitinib or abt 494 or abt494 or Rinvoq).ti,ab,kw.
22. (Filgotinib or Jyseleca).ti,ab,kw.
23. (baricitinib or "incb 028050" or incb 28050 or incb028050 or incb28050 or ly 3009104 or ly3009104 or Olumiant).ti,ab,kw.
24. or/17-23 [JAKs]
25. 16 and 24 [IMIDs and JAKs]

© 2025 Solitano V et al. *JAMA Network Open*.

26. exp Tumor Necrosis Factor Inhibitors/ use medall,cctr
27. exp tumor necrosis factor inhibitor/ use oemezd
28. ((TNF* or tumor necrosis factor* or tumour necrosis factor* or Cachectin or Cachetin) adj3 (anti or inhibit* or block* or agent* or antagonist*)).ti,ab,kw.
29. Infliximab/
30. (Infliximab or "ABP 710" or ABP710 or Avakine or Avsola or cA2 or Flixabi or "GP 1111" or GP1111 or IFX or Inflectra or Ixifi or "PF 06438179" or "PF 6438179" or PF06438179 or PF6438179 or Remicade or Remsima or Renflexis or Revellex or "TA 650" or TA650 or Zessly).ti,ab,kw.
31. Adalimumab/
32. (Adalimumab or Humira or Amjevita or Cyltezo or "D2E7 Antibody" or Trudexa or "abp 501" or abp501 or "abt d2e7" or abtd2e7 or adaly or amgevita or "avt 02" or avt02 or "bat 1406" or bat1406 or "bax 2923" or "bax 923" or bax2923 or bax923 or "bi 695501" or bi695501 or "chs 1420" or chs1420 or "ct p17" or ctp17 or "da 3113" or da3113 or "dmb 3113" or dmb3113 or exemptia or "fkb 327" or fkb327 or fyzoclad or "gp 2017" or gp2017 or hadlima or halimatoz or hefiya or "hlx 03" or hlx03 or hulio or hyrimoz or "ibi 303" or ibi303 or idacio or imraldi or kromeya or "lu 200134" or lu200134 or "m 923" or m923 or mabura or "msb 11022" or msb11022 or "ons 3010" or ons3010 or "pf06410293" or "pf 6410293" or pf06410293 or pf6410293 or raheara or "sb 5" or sb5 or solymbic or "zrc 3197" or zrc3197).ti,ab,kw.
33. ertolizumab Pegol/
34. (Certolizumab or "cdp 870" or cdp870 or cimzia or pf688 or pf 688 or "pha 738144" or pha738144 or "428863-50-7" or simziya or xcimzane).ti,ab,kw.
35. golimumab/
36. (Golimumab or "CNTO 148" or CNTO148 or mk 8259 or mk8259 or sch 900259 or sch900259 or shinponi or Simponi).ti,ab,kw.
37. Etanercept/
38. (Etanercept or tumor necrosis factor receptor Fc fusion protein or tumour necrosis factor receptor Fc fusion protein or "TNT Receptor Fusion Protein" or "TNTR-Fc" or "TNF Receptor Type II IgG Fusion Protein" or altebrel or avent or benepali or brenzys or "chs 0214" or chs0214 or davictrel or dwp 422 or dwp422 or embrel or enbrel or enerceptan or "enia 11" or enia11 or erelzi or etacept or etanar or eticovo or "gp 2015" or gp2015 or hd 203 or hd203 or infinitam or lifmior or nepexto or opinercept or reumatocept or "tnr 001" or tnr001 or tunex or yisaipu or "ylb 113" or ylb113).ti,ab,kw.
39. or/26-38 [TNFs]
40. 25 and 39 [IMIDs and JAKs and TNFs]
41. animals/ not humans/ use medall
42. animal/ not human/ use oemezd

© 2025 Solitano V et al. *JAMA Network Open*.

43. 40 not (41 or 42) [non animials]
44. limit 43 to yr="2012 -Current" [from 2012]
45. conference abstract.pt.
46. 44 not 45 [excluded conference abstracts]
47. limit 46 to english language [English]
48. child/ not adult/
49. 47 not 48 [excluded children]
50. case reports/ use medall
51. case report/ use oemezd
52. (case report or case series).ti.
53. 49 not (50 or 51 or 52) [excluded case reports before deduplicates]
54. remove duplicates from 53

© 2025 Solitano V et al. *JAMA Network Open*.

eMethods

Data Sources and Searches

We conducted a systematic literature search across multiple electronic databases, including Ovid Medline, Ovid EMBASE, and Web of Science, from inception to June 25, 2025. We used a combination of controlled vocabulary and keywords to identify studies reporting the risk of VTE, MACE, serious infections and cancers in patients with IMIDs treated with JAK inhibitors or TNF antagonists. We also manually searched the references of selected articles and review papers for additional studies. Furthermore, we searched clinical trial registries (clinicaltrials.gov, clinicaltrialsregister.eu) for unpublished or ongoing studies. This study was based on a systematic review and meta-analysis of previously published observational studies and did not involve direct interaction with human subjects or access to identifiable personal data. As such, it was deemed exempt from institutional ethics review. For each included study, we relied on the ethical approvals and informed consent procedures as reported by the original study authors.

Study Selection

We included real-world comparative cohort studies that met the following criteria: adults with immune-mediated inflammatory diseases (IMIDs), including RA, IBD, PsO/PsA, and SpA, treated with JAK inhibitors or TNF antagonists, and reported risk of serious infections, malignancy, MACE and/or VTE.

We excluded randomized clinical trials, non-comparative observational studies (e.g., studies evaluating safety risk with JAK inhibitors vs. a non-exposed group), studies that did not report outcomes of interest or focused solely on specific safety events (e.g., zoster or specific opportunistic infections) and studies with a sample size less than 500.

Two authors (VS, SS) independently reviewed the titles and abstracts to exclude irrelevant studies based on predefined inclusion and exclusion criteria. Full texts of remaining studies were reviewed independently for eligibility.

Data Extraction and Quality Assessment

Four investigators (VS, DA, HHL, RG) independently, and in pairs, abstracted data from included studies using a standardized data abstraction form, after initially piloting the form. The following data were extracted: (a) study characteristics: primary author, study

© 2025 Solitano V et al. *JAMA Network Open*.

design and source of data (e.g., database based: clinical registry, administrative claims data, or electronic health records), country of origin, time period, factors pertinent to risk of bias assessment; (b) patient characteristics: age, sex, smoking status, comorbidities like hypertension, diabetes, obesity, hyperlipidemia, prior CAD and other relevant outcome specific comorbidities; IMID subtype; (c) disease specific characteristics: type of IMID (RA, IBD, PsO/PsA, SpA), use of concomitant immunomodulators and corticosteroids (d) exposure characteristics: treatment with JAK inhibitors or TNF antagonists, duration of therapy; (d) outcomes studied: type and definition of serious infection, malignancy, MACE, and VTE; and (e) risk estimate variable: incidence rate (IR), hazard ratio (HR), risk ratio; (f) analytic approach for calculating estimate: methods to control for bias including use of propensity score matching, use of propensity score weights and inclusion of time-varying covariates; potential confounding variables accounted for in the analysis including disease activity (objectively or via surrogates), disease duration, risk factors including comorbidities (hypertension, dyslipidemia), prior safety events, and concomitant use of IMID related-and other medications. Of note, for studies that did not report IR, we used total person-year follow-up reported in the study or estimated based on mean or median follow-up.

Risk of bias was assessed by two sets of investigators independently using the Newcastle-Ottawa Scale[1].

Outcomes Assessed

The primary outcomes were the risk of serious infections (defined as infection requiring hospitalization, need for intravenous antibiotics, requiring cessation of immunosuppressive therapy and/or causing death), malignancy (excluding non-melanoma skin cancer), MACE (defined as hospitalization with myocardial infarction, stroke, and/or cardiovascular mortality) and VTE (defined as composite of pulmonary embolism and/or deep venous thrombosis). Most outcomes were ascertained based on administrative claims codes.

To assess the stability of the association between JAK inhibitors vs. TNF antagonists and the risk of safety outcomes, and to explore potential sources of heterogeneity, we conducted several pre-specified subgroup analyses. These included study location (North America, Europe and Asia), IMID type (RA vs. IBD vs. PsO/PsA vs. SpA), JAK inhibitor type (tofacitinib vs. baricitinib vs. upadacitinib), age group (younger vs. older patients, specifically those >50–65 years at the time of biologic initiation), and outcome subtype (malignancy: solid organ vs. hematological; MACE: myocardial infarction vs. stroke; VTE: pulmonary embolism vs. deep venous thrombosis). We also performed a meta-regression analysis to assess the impact of study-level characteristics on the overall

© 2025 Solitano V et al. *JAMA Network Open*.

effect size, including mean age, sex distribution (proportion males), midpoint of study recruitment (to examine whether change in regulatory guidance in 2019 modified the risk association between JAK inhibitors vs. TNF antagonists), concomitant immunomodulator and/or corticosteroid use, and prevalence of major comorbidities including diabetes, smoking status, hypertension, hyperlipidemia, prior coronary artery disease, and obesity. We conducted sensitivity analyses after excluding studies that did not adjust for key confounders.

Data Synthesis and Analysis

We performed a random-effects meta-analysis described by Hartung-Knapp to estimate summary risk of adverse safety outcomes by calculating the pooled estimates of HR (and their 95%) for safety outcomes in JAK inhibitors vs TNF antagonists[2]. To account for confounding, we used HRs adjusted for covariates. In cases where HRs were not available, we estimated unadjusted incidence rate ratios (IRRs) based on the reported incidence rates of events in each treatment group. Effect estimates and their standard errors were incorporated into the meta-analysis using the inverse-variance method, which assigns weights to individual studies according to the precision of their estimates. To account for inter-study variance, restricted maximum-likelihood (REML) was utilized to estimate variability between the studies[3, 4]. The I^2 statistic which quantifies the percentage of total variation across studies attributed by heterogeneity between studies rather than sampling error was calculated using chi-squared statistic and degrees of freedom[5, 6]. The Hartung-Knapp adjustment was applied for random effects model as it provides more accurate and conservative confidence intervals[2, 7]. However, Hartung-Knapp method uses t-distribution with (k-1) degrees of freedom which can amplify the width resulting in extremely wide confidence intervals when inter-study variance is large. In those instances, we used DerSimonian-Liard method.

To further study the characteristics, sub-group analyses were conducted by using the variable of interest as sub-group in the meta-analysis (as described above). In this analysis, a p-value for differences between sub-groups of <0.10 was considered statistically significant. Heterogeneity across studies was assessed using the I^2 statistic, where values of <30%, 30%-60%, 60%-75%, and >75% indicated low, moderate, substantial, and considerable heterogeneity, respectively. To account for the impact of heterogeneity by different variables and the impact of these variables on the estimate, univariable meta-regression based on study-level continuous variables, and a p-value for differences between subgroups of <0.10 was considered statistically significant. Publication bias was

© 2025 Solitano V et al. *JAMA Network Open*.

assessed qualitatively using funnel plots when >10 studies were identified for a comparison, and asymmetry of funnel plots was quantitatively assessed using the Egger's test of the intercept[8]. All analyses were performed using "*meta*" package in R version 4.3.2.

PROTOCOL

Review title

Comparative safety with JAK vs. TNF inhibitors in immune-mediated inflammatory diseases (IMIDs): a systematic review and meta-analysis of observational studies

Original language title

English

Review objectives

To compare the risk of serious adverse events—including serious infections, malignancy, major adverse cardiovascular events (MACE), and venous thromboembolism (VTE)—with JAK inhibitors versus TNF antagonists in adult patients with IMIDs using real-world comparative cohort data.

SEARCHING AND SCREENING

Searches

We searched Ovid MEDLINE, Ovid EMBASE, and Web of Science from inception to June 25, 2024. The search strategy combined MeSH terms and keywords related to IMIDs, JAK inhibitors, and TNF antagonists. References of selected articles and reviews were also screened, and clinical trial registries (e.g., ClinicalTrials.gov) were searched. There were no language restrictions during the search phase, but included studies were in English.

ELIGIBILITY CRITERIA

Condition or domain being studied

© 2025 Solitano V et al. *JAMA Network Open*.

Immune-mediated inflammatory diseases (IMIDs), including rheumatoid arthritis (RA), inflammatory bowel disease (IBD), psoriasis/psoriatic arthritis (PsO/PsA), and spondyloarthropathy (SpA).

Population

Adults (≥18 years) diagnosed with any IMID and treated with JAK inhibitors or TNF antagonists.

Intervention(s)

Treatment with any JAK inhibitor (e.g., tofacitinib, baricitinib, upadacitinib, filgotinib).

Comparator(s)

Treatment with any TNF antagonist (e.g., infliximab, adalimumab, certolizumab, golimumab, etanercept).

Study Design

We included comparative real-world cohort studies. Randomized clinical trials were excluded. Studies needed to report at least one outcome of interest with both treatment groups.

OUTCOMES TO BE ANALYSED

Primary outcomes

- Serious infections (requiring hospitalization, IV antibiotics, therapy cessation, or causing death)
- Malignancy (excluding non-melanoma skin cancer)
- MACE (myocardial infarction, stroke, cardiovascular mortality)
- VTE (deep vein thrombosis or pulmonary embolism)

Secondary/Additional analyses

- Subgroup analyses by IMID type, JAK inhibitor type, age group, and comorbidity profile
- Meta-regression by study-level characteristics including age, sex, smoking, comorbidities, corticosteroid use

© 2025 Solitano V et al. *JAMA Network Open*.

- Sensitivity analyses excluding studies without adjustment for confounding

---

DATA COLLECTION PROCESS

Study Selection and Data Extraction

Titles and abstracts were screened by two reviewers independently. Full texts were then assessed for eligibility. Data were extracted in duplicate using a standardized form. Discrepancies were resolved through consensus or adjudication by a third reviewer.

Extracted data included

- Study characteristics: country, data source, period, design
- Patient demographics and disease characteristics
- Treatment details and follow-up duration
- Outcomes (IR, HR) and statistical methods used

RISK OF BIAS ASSESSMENT

Risk of bias for each included study was independently assessed by two reviewers using the Newcastle-Ottawa Scale (NOS). Disagreements were resolved through consensus. Publication bias was assessed using funnel plots and Egger's test if ≥10 studies were available for an outcome.

PLANNED DATA SYNTHESIS

Strategy for data synthesis

We performed a random-effects meta-analysis using the Hartung-Knapp method to pool adjusted hazard ratios (HRs) for each safety outcome. For studies lacking HRs, unadjusted incidence rate ratios (IRRs) were calculated. Heterogeneity was assessed using the I^2 statistic.

© 2025 Solitano V et al. *JAMA Network Open*.

Subgroup analysis

Planned subgroups included:

- Type of IMID (RA vs. IBD vs. PsA vs. SpA)
- Type of JAK inhibitor (e.g., tofacitinib vs. baricitinib)
- Geographic region
- Age and comorbidity strata
- Type of adverse event (e.g., solid vs. hematological malignancy)

Sensitivity analysis

- Excluding studies without key confounder adjustment
- Fixed vs. random-effects model
- Meta-regression to explore heterogeneity

© 2025 Solitano V et al. *JAMA Network Open*.

eTable 1. Characteristics of Included Studies

Study	Geographic location; time period	Disease type	Study design; Database	Comparison (and # of patients in each group), and follow-up in each group (mean [SD] or total person-years)	Definition of outcome of interest	Analysis approach
Ahuja,[9] 2025	USA<br>January 1, 2016, to June 30, 2023	IBD	Retrospective; administrative claims from a large database, OptumLabs® Data Warehouse	TNFi (n=9422)<br>28.4 (8.4) months<br><br>JAKi (n=856)<br>26.7 (8.3) months	Serious infections: infection (identified in top five diagnoses) requiring hospitalization<br><br>VTE: deep venous thrombosis and/or pulmonary embolism during inpatient, emergency room and/or office visit, identified based on top five visit diagnoses (ICD-10 codes)<br><br>MACE: composite of myocardial infarction, unstable angina, cardiovascular revascularization procedure, stroke (ischemic or hemorrhagic) or TIA	Cox proportional hazards with stabilized IPTW

© 2025 Solitano V et al. *JAMA Network Open*.

Bastard,[10] 2024	France 2015-2021	Psoriatic arthritis	Retrospective; Administrative healthcare claims dataset	TNF antagonists<br>• Adalimumab (n=4616): 12.6 months (6.2–29.5)<br>• Etanercept (n=2623): 13.4 months (5.6–36.5)<br>• Golimumab (n=954): 14.0 months (6.5–36.2)<br>• Certolizumab (n=683): 11.2 months (5.5–27.9)<br>• Infliximab: (n=70) 10.7 months (6.1–32.7)<br><br>JAK inhibitors (n=152): 8.5 months (4.7–14.3)	Serious infections as any hospitalization with a discharge main diagnosis of an infection (ICD-10 codes)	Cox proportional hazards with IPTW
Bower,[11] 2023	Sweden 2026-2021	Rheumatoid arthritis	Prospective; National healthcare database	TNF antagonists (n=9525): 1.7 (2.7-7.0) yrs<br><br>JAK inhibitors (n=2697): 1.6 (2.5-5.9) yrs	MACE ICD-10 codes	Cox proportional hazards
Huss,[12] 2022	Sweden 2001-2018	Rheumatoid arthritis	Prospective; National healthcare database	TNF antagonists (n=21,365):<br>6.6 (3.1–10.9) yrs<br><br>JAK inhibitors (n=1289): 0.7 (0.3–1.1)	Cancer ICD-10 codes excluding cutaneous	Cox proportional hazards
Huss,[13] 2023	Sweden 2016-2020	Rheumatoid arthritis or psoriatic arthritis	Prospective; National healthcare database	TNF antagonists (n=7343): 2.5 yrs<br><br>JAK inhibitors (n=1967): 2.0 yrs	Cancer ICD-10 codes	Cox proportional hazards
Khosrow-Khavar,[14] 2022	USA 2012-2020	Rheumatoid arthritis	Retrospective, insurance claims data from Optum	**Optum**<br>• TNF antagonists (n=21934): 13.7 (13.2) months	Cancer ICD-10 excluding NMSC	Cox proportional hazards with PS fine-

© 2025 Solitano V et al. *JAMA Network Open*.

			Clinformatics, IBM MarketScan, and Medicare	• JAK inhibitors (n=3301): 16.1 (14.9) months<br>**MarketScan**<br>• TNF antagonists (n=24960): 14.0 (12.8) months<br>• JAK inhibitors (n=4499): 16.1 (14.1) months<br>**Medicare**<br>• TNF antagonists (n=25673): 13.4 (11.6) months<br>• JAK inhibitors (n=2689): 17.3 (13.7) months		stratification weighting
Min,[15] 2023	Korea	Rheumatoid arthritis	Retrospective; Administrative data	Set 1<br>• TNF antagonists (n=951): 2.5y<br>• JAK inhibitors (n=645): 2.1y<br><br>Set 2<br>• TNF antagonists (n=9267): 5.8y<br>• JAK inhibitors (n=2498): 2.6y	Acute myocardial infarction, stroke, cardiovascular-related mortality, MACE, CTE, arterial thromboembolism, cancer, and all-cause mortality ICD-10	Cox proportional hazards
Uchida,[16] 2023	Japan 2013-2020	Rheumatoid arthritis	Retrospective; medical record review	TNF antagonists (n = 203) : 1.4y (0.4–3.1)<br>Tofacitinib (n = 192): 2.0y (0.5–3.8) yrs<br>Baricitinib (n = 104): 0.8y (0.3–1.5) yrs	Serious infectious diseases according to treating physician	Cox proportional hazards

© 2025 Solitano V et al. *JAMA Network Open*.

					MACE as composite of cardiovascular death, non-fatal myocardial infarction, and non-fatal stroke by attending rheumatologist<br><br>Cancer as cases of malignancy confirmed by the attending rheumatologist or physician	
Kremer,[17] 2021	USA<br>2012-2018	Rheumatoid arthritis	Retrospective; National healthcare database	For MACE, serious infections,<br>• Biologic DMARD (n=8358): 12869.4 PY<br>• Tofacitinib (n=1999): 3152.1 PY<br>For malignancy/death<br>• Biologic DMARD (n=6354): 16670.8 PY<br>• Tofactinib (n=1999): 4505.6 PY	MACE as myocardial infarction, stroke/ transient ischemic attack, and cardiovascular death<br><br>Serious infections leading to hospitalization and/or intravenous antibiotics<br><br>Cancer as malignancy excluding NMSC<br><br>VTE (DVT or PE)	Cox proportional hazards with PS matching

© 2025 Solitano V et al. *JAMA Network Open*.

Bilgin,[18] 2022	Turkey<br>2005-2022	Rheumatoid arthritis	Retrospective; National healthcare database	Etanercept (n=114): 356.2 (316.4–396.0) PY<br><br>Tofacitinib (n = 145): 412.8 (336.5–489.2) PY	Cancers excluding NMSC<br><br>VTE<br><br>MACE as cardiovascular death<br>(not due to PE), non-fatal myocardial infarction, or non-fatal stroke	Unadjusted incidence rate ratio
Cheng,[19] 2022	USA 2008-2019	Inflammatory bowel diseases	Retrospective; National commercial health insurance plan	TNF antagonists (n= 19096): 366 (411) days<br><br>Tofacitinib (n=305): 227 (211) days	Infection-related hospitalization as having<br>any medical claim during the follow-up with the place of service at an emergency room or inpatient hospital and an infection-related ICD diagnosis code in the primary position	Cox proportional hazards with PS weighting
Cho,[20] 2024	Korea<br>2014-2021	Rheumatoid arthritis	Prospective; medical record review	TNF antagonists (n= 455): 11.2 (10.9, 11.9) months<br><br>Tofacitinib (n=200): 11.5 (11.1, 11.9) months	AEs and SAEs	Adjusted incidence rate
Choi,[21] 2023	Korea 2000-2019	Rheumatoid arthritis	Retrospective; National healthcare database	TNF antagonists (n=13152)<br>JAK inhibitors (n=2967)	Infections according to ICD codes	Cox proportional hazards with PS weighting

© 2025 Solitano V et al. *JAMA Network Open*.

				Mean follow-up 1.2 yrs		
de Avila Machado,[22] 2018	USA,<br>2010-2014	Rheumatoid arthritis	Retrospective; administrative healthcare claims dataset	TNF antagonists (n=13,367)<br>Tofacitinib (n=164)<br><br>12 months	Serious infections occurrence of infection requiring hospitalization identified from the primary diagnosis recorded in the hospitalization discharge records (ICD-9 codes)	Cox proportional hazards
Fang,[23] 2022	Taiwan, 2015-2017	Rheumatoid arthritis	Retrospective; National healthcare database	TNF antagonists (n=2357): 2.1y<br><br>JAKi (n= 822): 2.0y	CHD, stroke, overall thromboembolism, DVT, malignancy, and all-cause mortality according to ICD-codes	Cox proportional hazards with PS stratification weighting
Frisell,[24] 2023	Sweden 2010-2020	Rheumatoid arthritis	Retrospective; National healthcare database	TNF antagonists<br>• Adalimumab (n=5526): 13566.1 PY<br>• Etanercept (n=8748): 26508.3PY<br>• Golimumab (n=1889): 6051.1 PY<br>• Certolizumab (n=2179): 6180.3 PY<br>• Infliximab (n=2971): 8217.7 PY<br>JAK inhibitors<br>• Tofacitinib (n=426): 648.9 PY<br>• Baricitinib (n=1837): 3401.8 PY	MACE, including acute coronary syndrome, stroke and fatal cardiovascular events)<br><br>Serious infection as requiring inpatient treatment	Cox proportional hazards

© 2025 Solitano V et al. *JAMA Network Open*.

Hernández-Cruz,[25] 2024	Spain, 2015-2023	Rheumatoid arthritis, psoriatic arthritis and SpA	Retrospective; National healthcare database	Rheumatoid arthritis<br>• TNF antagonists (n=2681): 1.8y (1.8)<br>• JAK inhibitors (n=1386): 1.9y(1.6)<br><br>Psoriatic arthritis<br>• TNF antagonists (n=1673): 2.2y (2.1)<br>• JAK inhibitors (n=198): 1.3y (1.2)	AEs according to the Medical Dictionary for Regulatory Activities (version 26.1)	Cox proportional hazards
Hoisnard,[26] 2022	France, 2017-2021	Rheumatoid arthritis	Retrospective; National healthcare database	TNF antagonists (adalimumab) (n=7354): 344 (185–686) days<br><br>JAK inhibitors<br>Tofacitinib (n=3416)<br>Baricitinib (n=5065)<br><br>440 (203–846) days	MACE identified on hospital discharge with (1) ICD-10 codes I21 and I24 for acute myocardial infarction or (2) ICD-10 codes<br><br>VTE as hospital discharge with ICD-10 codes	Cox proportional hazards with IPTW
Kochar,[27] 2021	USA, 2008-2019	Inflammatory bowel diseases	Retrospective; administrative healthcare claims dataset	TNF antagonists (n= 19,096): 793.1 (716.3) days<br><br>Tofacitinib (n=305): 372.8 (382.3) days	VTE event or cardiovascular event according to ICD-9/10 codes	Cox proportional hazards with IPTW
Ma,[28] 2024	Transcontinental 2018-2020	Rheumatoid arthritis	Retrospective, using TriNetX	TNF antagonists<br>• Adalimumab (n=5,582)<br>JAK inhibitors	Dyslipidemia and MACE according to ICD-10 codes	Cox proportional

© 2025 Solitano V et al. *JAMA Network Open*.

			collaborative network	• Tofacitinib (n=1998)<br>Within 3 yrs		hazards with PS matching
Khosrow-Khavar,[29] 2022	USA, 2012-2020	Rheumatoid arthritis	Retrospective new user, active comparator cohort study using claims data from the Optum Clinformatics, IBM MarketScan, and Medicare databases	Optum<br>• TNF antagonists (n=24,688)<br>• Tofacitinib (n=3,761)<br><br>MarketScan<br>• TNF antagonists (n=28,727)<br>• Tofacitinib (n=5,298)<br><br>Medicare<br>• TNF antagonists (n=35,816)<br>• Tofacitinib (n=3,782)<br>12 months	Composite CV outcome consisting of hospitalizations for myocardial infarction or stroke.	Cox proportional hazards with PS fine stratification weighting
Meissner,[30] 2023	Germany, 2007-2022	Rheumatoid arthritis	Prospective; National healthcare database	TNF antagonists (n=3694): 19.2 (15.3) months<br><br>JAK inhibitors (n=3058): 18.2 (14.3) months	MACE as the composite of non-fatal or fatal stroke or myocardial infarction, and other cardiovascular death by the rheumatologist	Cox proportional hazards with IPTW
Mok,[31] 2024	China, 2008-2021	Rheumatoid arthritis	Retrospective; National healthcare database	TNF antagonists (n=1920): 5315 PY<br><br>JAK inhibitors (n=551): 1116 PY	MACE ischemic vascular events on record<br><br>Cancer as clinical diagnosis of malignant tumors	Cox proportional hazards

© 2025 Solitano V et al. *JAMA Network Open*.

					that were documented<br><br>Serious infections hospitalization, requiring intravenous antibiotic or anti-viral therapy or resulting in significant morbidity or mortality	
Molander,[32] 2022	Sweden, 2010-2021	Rheumatoid arthritis	Retrospective; National healthcare database	TNF antagonists (n=15090) : 2 (1-4) yrs<br><br>JAK inhibitors (n=2150) : 2 (1-3) yrs	VTE according to ICD-10 code in primary or secondary position in the NPR, or PE listed as the underlying cause of death in the Cause of Death Register	Cox proportional hazards
Ozen,[33] 2021	USA, 1998-2017	Rheumatoid arthritis	Retrospective; National healthcare database	TNF antagonists (7724): 21983 PY<br><br>Tofacitinib (n=301): 2301 PY	Composite CVD events (myocardial infarction, stroke, heart failure, and CVD-related death validated from hospital/death records)	Cox proportional hazards
Pawar,[34] 2020	USA, 2012-2017	Rheumatoid arthritis	Retrospective cohort study using claims data from	(Medicare, Optum, MarketScan)<br>TNF antagonists<br>• Adalimumab (n=10205;20752;5320)	Composite endpoint of admission to hospital for serious infection including	Cox proportional hazards with PS weighting

© 2025 Solitano V et al. *JAMA Network Open*.

			Medicare fee-for-service, Optum Clinformatics, and IBM MarketScan	• Etanercept (n=8128;20826;7007)<br>• Golimumab (n=1911;2508;2089)<br>• Certolizumab (n=2137;2277;3852)<br>• Infliximab (n=2597;6029;8897)<br><br>JAK inhibitors<br>• Tofacitinib (n=1705; 2090; 1204)<br><br>Median follow-up time in the as-treated analysis ranged from 168 days to 182 days	bacterial, viral, or opportunistic infection according to ICD-9/10 codes	
Sendaydiego,[35] 2024	USA, 2012-2018	Rheumatoid arthritis	Retrospective cohort study using MerativeTM Marketscan® Research Databases	TNF antagonists (n=20,586): 300 (180-550) days<br><br>JAK inhibitors (n= 2,570): 270 (170-450) days	Cancer excluding NMSC ICD-9/10 codes	Cox proportional hazards with PS
Sendaydiego,[36] 2024	USA, 2012-2021	Rheumatoid arthritis	Retrospective cohort study using MerativeTM Marketscan® Research Databases	TNF antagonists (n = 27,085):<br>240 (120, 470) days<br><br>JAK inhibitors (n = 3,790): 190 (90, 360) days	MACE as a composite of myocardial infarction, cardiac arrest, sudden death, stroke, percutaneous coronary intervention, and coronary artery bypass graft,	Cox proportional hazards

© 2025 Solitano V et al. *JAMA Network Open*.

					identified using at least one ICD-9/10 codes	
Sakai,[37] 2024	Japan, 2023-2020	Rheumatoid arthritis	Retrospective; administrative healthcare claims dataset	Biologic DMARD (n=20964): 31 (15–57) months<br>JAK inhibitors (n=4416): 26 (12–43) months	Cardiovascular events (VTE, deep vein thrombosis, PE, arterial thrombosis, AMI, stroke) were identified via ICD-10 codes + treatment during hospitalization. CV death not an outcome.	Cox proportional hazards
Salinas,[38] 2023	USA, Europe, and Japan (transcontinental)<br><br>2020-2023	Rheumatoid arthritis	Retrospective; administrative healthcare claims dataset	TNF antagonists<br>HealthVerity: 748<br>ARTIS: 1685<br>SNDS: 2859<br>6512 PY<br><br>Baricitinib<br>HealthVerity: 748<br>ARTIS: 1685<br>SNDS: 2859<br>5879 PY	VTE, as a composite of PE, DVT, or other venous thrombosis<br><br>MACE as myocardial infarction or ischemic or hemorrhagic stroke.<br><br>Serious infection based on primary ICD-10 diagnosis codes	Incidence rate ratio with PS matching

© 2025 Solitano V et al. *JAMA Network Open*.

Song,[39] 2022	Korea, 2015-2019	Rheumatoid arthritis	Retrospective; administrative healthcare claims dataset	TNF antagonists (n=3865): 1.8±1.3 yrs<br><br>JAK inhibitors (n=1064): 1.2±0.7 yrs	Cancer as appearance of a new malignancy according to ICD-10 codes	Cox proportional hazards with IPTW
Song,[40] 2023	Korea, 2015-2019	Rheumatoid arthritis	Retrospective; administrative healthcare claims dataset	TNF antagonists (n=3307): 5,940.3 PY<br><br>JAK inhibitors (n=871): 1,029.2 PY	VTE, including both PE and DVT ICD-10 codes	Cox proportional hazards with IPTW
Song,[41] 2023	Korea, 2013-2018	Rheumatoid arthritis	Retrospective; administrative healthcare claims dataset	Biologic DMARD (n=3384): 6.5 months<br><br>JAK inhibitors (n=846): 6.5 months	MACE as myocardial infarction, ischemic stroke, coronary revascularization such as angioplasty or bypass surgery according to ICD-10 codes	Cox proportional hazards with PS matching
Tong,[42] 2023	Hong-Kong, Korea, Taiwan 2010-2020	Rheumatoid arthritis	Retrospective; National healthcare database from Hong Kong, Taiwan and Korea	TNF antagonists (n=5849): 1.85 (3.41); 1.87 (2.82);1.63 (2.83) yrs<br><br>JAK inhibitors (n=1571): 0.91 (1.38); 1.32 (1.39); 1.02 (1.30) yrs	Hospitalized CVE (including acute coronary heart diseases, stroke, heart failure, venous thromboembolism events and systemic embolism) according to ICD-9/10 codes	Unadjusted incidence rate ratio

© 2025 Solitano V et al. *JAMA Network Open*.

Desai,[43] 2021	USA,2012-2019	Rheumatoid arthritis	Retrospective using claims data from the IBM 'MarketScan', Medicare or 'Optum' Clinformatics databases	TNF antagonists<br>• MarketScan (n=39209)<br>• Medicare (n=23283)<br>• Optum (n=18387)<br>6 months<br><br>JAK inhibitors<br>• MarketScan (n=2992)<br>• Medicare (n=1795)<br>• Optum (n=1987)<br>6 months	VTE including PE or DVT based on inpatient diagnoses	Cox proportional hazards with PS
Westermann,[44] 2024	Denmark, 2017-2020	Rheumatoid arthritis	Retrospective; National healthcare database	bDMARD (n=8597): 1.98y (1.11–3.09)<br><br>JAK inhibitors<br>• Tofacitinib (n=694)<br>• Baricitinib (n=181)<br>1315 PY (1.48; 0.98–1.93)	Cancer diagnosis excluding NMSC according to ICD-10 codes	Cox proportional hazards with IPTW
Cho[45], 2025	South Korea	Rheumatoid arthritis and ulcerative colitis	Retrospective; administrative healthcare claims dataset	TNF antagonists<br>Rheumatoid arthritis (n=4992) 1.58 yrs<br>Ulcerative colitis (n=548) 1.14 yrs<br><br>JAK inhibitors<br>Rheumatoid arthritis (n=4992) 1.84 yrs<br>Ulcerative colitis (n=548) 1.41 yrs	3P-MACE defined as the composite of nonfatal myocardial infarction, nonfatal stroke, and cardiovascular death<br><br>Cancer excluding NMSC ICD-9/10 codes<br><br>Serious infection	Cox proportional hazards with PS

© 2025 Solitano V et al. *JAMA Network Open*.

					based on primary ICD-10 diagnosis codes	
Zhao[46], 2025	Global network TriNetX (transcontinental)	PsA<br>Axial SpA	Retrospective, using TriNetX US collaborative network	TNF antagonists (n=2200) 4618 PY<br>JAK inhibitors (n=2200) 3092 PY	CVD: coronary artery disease (myocardial infarction, other acute ischaemic heart diseases, coronary revascularization) or stroke (ischaemic stroke, cerebral revascularization, non-traumatic intracerebral haem-orrhage)<br><br>Cancer as common solid cancers	Cox proportional hazards with PS
Aymon[47], 2025	15 registries worldwide (transcontinental)	Rheumatoid arthritis	Retrospective; National healthcare database	TNF antagonists (n=25,049)<br>Etanercept<br>Adalimumab<br>Certolizumab<br>Infliximab<br>Golimumab<br>1.33 yrs<br><br>JAK inhibitors (n=13,374)<br>Baricitinib<br>Tofacitinib<br>Upadacitinib<br>Filgotinib	MACE as defined as the occurrence of a stroke, MI, or transient ischemic attack (ICD-9 and 10)	Adjusted IRR

© 2025 Solitano V et al. *JAMA Network Open*.

				1.39 yrs		
Silvagni,[48] 2025	Italy, 2020-2023	Rheumatoid arthritis	Retrospective; National healthcare database	TNF antagonists (n=2343) 3851 PY<br>JAK inhibitors (n=1443) 3173 PY	CV events according to ICD-9	Adjusted time-dependent Cox regression
Kao,[49] 2025	Taiwan, 2001-2020	Rheumatoid arthritis	Retrospective; administrative healthcare claims dataset Taiwan National Health Insurance	TNF antagonists (n=8902)<br>For MACE, 25,277 PY<br>For VTE, 25,401 PY<br><br>JAK inhibitors (n=1462)<br>For MACE, 2986 PY<br>For VTE, 3010 PY	MACE defined as the composite of myocardial infarction necessitating ⩾3 days of admission unless death, ischemic stroke, CV mortality, coronary artery bypass graft or procedures of coronar revascularization<br><br>VTE as a composite of DVT or PE	Cox proportional hazard
Tanaka,[50] 2025	Japan, 2016-2022	Rheumatoid arthritis	Prospective; National healthcare database	TNFi (n = 663) 3 yrs<br>Tofacitinib (n = 253) 2.9 yrs	MACE defined as non-fatal myocardial infarction, non-fatal stroke, and cardiovascular deaths, excluding fatal pulmonary embolism.	Adjusted incidence ratio

© 2025 Solitano V et al. *JAMA Network Open*.

eTable 2. Characteristics of Patients in Included Studies

© 2025 Solitano V et al. *JAMA Network Open*.

Study	Exposure	Disease type	# of patients	Age (mean, SD)	% females	Disease duration, year (mean, SD)	% on steroids	% on con IMM	% with prior biologic exposure	Comorbidities						
										HBP	DM	Dyslipidemia	Obesity	CKD	CHF	Other [illegible]
Ahuja[9] 2025	TNF antagonists	IBD	9422	45.1 (17.8)	51.1	N/A	42.4	19.5%	< 0.1%	29.6	10.9	23	14.1	4.6	NA	NA
	JAK inhibitors	IBD	856	47.4 (16.6)	52.9	N/A	48.5	14.8	30.6 %	30.5	11.3	25	14.8	5.1	NA	NA
Bastard[10] 2024	TNF antagonists<br>Adalimumab<br>Etanercept<br>Golimumab<br>Certolizumab<br>Infliximab	PsA	 4616<br>2623<br>954<br>683<br>70	 48.0<br>(12.5)<br>50.5<br>(13.2)<br>47.7<br>(12.3)<br>44.3<br>(12.7)<br>50.1<br>(14.8)	 55.0<br>59.3<br>58.4<br>75.1<br>58.6	N/A	 15.4<br>19.0<br>18.9<br>18.5<br>11.4	N/A	N/A		 8.3<br>9.3<br>6.1<br>5.9<br>10.0		 9.2<br>9.3<br>8.9<br>12.0<br>8.6	 0.2<br>0.3<br>0<br>0.3<br>0	 0.6<br>0.9<br>0.2<br>0.3<br>1.4	Pso<br>IBD<br>Uve
	JAK inhibitors<br>Tofacitinib	PsA	 152	 52.7<br>(13.0)	 75.0	N/A	 29.0	N/A	N/A		 7.9		 6.6	 0.7	 3.3	Pso<br>IBD<br>Uve
Bower[11] 2023	TNF antagonists	RA	9525	58 (47-68)	77	7.0 (2.5-14.7)	47	MTX 63%	37		10			1	0	
	JAK inhibitors	RA	2697	60 (51-70)	82	13.3 (6.9-22.7)	51	MTX 42%	87		11			1	1	
Huss[12] 2022	TNF antagonists	RA	21365	56 (46–65)	77	9 (3–17)	56	MTX 62%	N/A	15	6			1		
	JAK inhibitors	RA	1289	59 (49–69)	82	13 (7–22)	64	MTX 37%	N/A	27	9			2		
Huss[13] 2023	TNF antagonists	RA or PsA	7343	56 (46–67) 57	78	7.1 (2.6–14.7)	61	MTX 63%	37	9	10			1	1	
	JAK inhibitors	RA or PsA	1967	59 (50–69)	82	13.2 (6.8–22.3)	64	MTX 42%	88	14	10			2	2	

© 2025 Solitano V et al. *JAMA Network Open*.

Khosrow-Khavar[14] 2022	TNF antagonists	RA	Optum 21934	56.1 (13.1)	81.9	N/A	49.4	MTX 45.8 LEF 21.4	N/A	51.2	21.3	42	23.4	5	4.9	
			MarketScan 24960	54.0 (11.7)	82.4		48.8	MTX 50.6 LEF 19.7		43.4	15.4	36.9	15.4	3	2.9	
			Medicare 25673	71.4 (5.3)	86.0		55.8	MTX 51.5 LEF 22.8		82.0	30.4	65.8	16.0	10.8	10.9	
	JAK inhibitors Tofacitinib	RA	Optum 3301	55.8 (12.4)	81.5	N/A	49.5	MTX 46.1 LEF 21.3	N/A	50.6	21.3	41.5	23.2	5	4.7	
			MarketScan 4499	53.8 (11.4)	82.2		48.7	MTX 51.2 LEF 19.8		42.9	15.2	36.5	15.4	3	2.9	
			Medicare 2689	71.3 (5.4)	85.5		56.0	MTX 52.2 LEF 22.5		82.1	30.5	66.2	16.0	10.7	10.9	
Min[15] 2023	TNF antagonists	RA	Set1 951	50.16 ±14.44	72.3	N/A	85.7	N/A	N/A	24.4	15.7			0.6	0.8	
			Set2 9267	52.00 ±13.27	81.9		85.9			27.6	14.4			0.8	1.2	
	JAK inhibitors	RA	Set1 645	52.30 ±12.80	75.5	N/A	93.3	N/A	N/A	24	17.2			1.1	1.4	

© 2025 Solitano V et al. *JAMA Network Open*.

			Set2<br>2498	51.50<br>±12.25	83.2		90.4			27.1	15.9			1.1	1.5	
Uchida[16] 2023	TNF antagonists	RA	203	51 (37–61)	81.8	6 (2–14)	55.2	MTX 65.0	TNFi 34<br>IL-6i 14<br>Aba 17<br>JAKi 5		15.8					
	JAK inhibitors<br>Tofacitinib<br><br><br>Baricitinib	RA	 192<br><br><br>104	 67 (58–73)<br><br>68 (58–75)	 80.7<br><br><br>84.6	 11 (6–18)<br><br>12 (3–18)	 54.7<br><br><br>40.4	 MTX 70.3<br><br><br>MTX 44.2	 TNFi 59<br>IL-6i 53<br>Aba 31<br>TNFi 27<br>IL-6i 15<br>Aba 14<br>JAKi 24		 19.8<br><br><br>24.0					
Kremer[17] 2021	bDMARD	RA	8358	58.5 (13.0)	80.4	N/A	30.7	MTX 52.2	70.8	32.3	10.3	13.1				
	JAK inhibitors<br>Tofacitinib	RA	 1999	 59.6 (12.1)	 80.5	 N/A	 31.6	 MTX 36.1	 88.8	 35.5	 11.6	 13.5				
Bilgin[18] 2022	TNF antagonists<br>Etanercept	RA	114	47.7 (12.7)	72.8	6.4 (6.0)	N/A	81.6	N/A	19.3	6.1			0	0	
	JAK inhibitors<br>Tofacitinib	RA	 145	 53.1 (13.1)	 86.2	 7.9 (7.5)	 N/A	 75.9	 N/A	 33.1	 9.0			 0	 0	
Cheng[19] 2022	TNF antagonists	IBD	19096	39.2 (17)	51	1.9 (2.1)	N/A	12.7	0.8							
	JAK inhibitors<br>Tofacitinib	IBD	 305	 43.8 (15.9)	 56.4	 2.8 (2.7)	 N/A	 29.8	 36							
Cho[20] 2024	TNF antagonists	RA	455	51.3(39.9, 59.6)	84.6	5.3 (1.3, 11.6)	79.6	MTX 92.5	10.1		6.2				0.4	
	JAK inhibitors<br>Tofacitinib	RA	 200		 90.5		 75.5		 52.0		11.0				1.0	

© 2025 Solitano V et al. *JAMA Network Open*.

				54.6(43.8, 62.3)		9.8 (5.0, 15.5)		MTX 84.5								
Choi[21] 2023	TNF antagonists	RA	13152	53.8 ± 13.5	80.9	N/A	87.0	MTX 88.9 LEF 45.3 Tac 22.0 Cyc 2.5 Aza 1.2	17.3	35.3	24.5	51.6		2.6	2.9	
	JAK inhibitors	RA	2967	55.9 ± 12.7	83.7	N/A	84.5	MTX 87.1 LEF 39.0 Tac 28.2 Cyc 0.6 Aza 0.5	40.2	34.9	29.3	63.7		3.3	5.2	
de Avila Machado[22] 2018	TNF antagonists	RA	13367	56 (48–63)	76.1	N/A	66.6	N/A	N/A							
	JAK inhibitors Tofacitinib	RA	164	58 (50–64)	78.7	N/A	69.0	N/A	N/A							
Sendaydiego[35] 2024	TNF antagonists	RA	20,586	50 (41-55)	78	N/A	47	N/A	N/A							
	JAK inhibitors	RA	2,570	50 (43-56)	81	N/A	42	N/A	N/A							
Sendaydiego[36] 2024	TNF antagonists	RA	27,085	50 (42-56)	78	N/A	46	N/A	N/A							

© 2025 Solitano V et al. *JAMA Network Open*.

	JAK inhibitors	RA	3,790	50 (43-56)	81	N/A	43	N/A	N/A							
Frisell[24] 2023	TNF antagonists<br>Adalimumab<br>Etanercept<br>Golimumab<br>Certolizumab<br>Infliximab	RA	 5526<br>8748<br>1889<br>2179<br>2971	 58 (14)<br>58 (14)<br>57 (14)<br>56 (15)<br>58 (14)	 76<br>77<br>78<br>78<br>74	 8.4(11.5)<br>7.7(10.6)<br>8.9(10.9)<br>8.3(12.1)<br>6.8(10.8)	 40<br>43<br>41<br>47<br>44	 MTX 60<br>MTX 59<br>MTX 66<br>MTX 56<br>MTX 76	 50<br>38<br>52<br>47<br>25		 7.1<br>7.2<br>5.7<br>6.7<br>6					
	JAK inhibitors<br>Tofacitinib<br>Baricitinib	RA	 426<br>1837	 59 (13)<br>61 (14)	 82<br>82	 13.1(11.1)<br>13.2(11.7)	 52<br>45	 MTX 35<br>MTX 39	 91<br>85		 8.9<br>8					
Hernández-Cruz[25] 2024	TNF antagonists	RA<br>All SpA	2681<br>3218	55.7 (12.4)<br>49.4 (12.5)	N/A	9.3 (8.7)<br>8.9 (9.3)	57.5<br>17.4	N/A	46.2<br>52.0							
	JAK inhibitors	RA<br>All SpA	1386<br>256	57.0 (12.1)<br>52.6 (10.3)	N/A	11.8 (8.8)<br>11.2 (9.1)	56.9<br>39.2	N/A	76.2<br>90.3							
Hoisnard[26] 2022	TNF antagonists<br>Adalimumab	RA	 7354	 55.3 (13.4)	 71.2	 N/A	 32.0	 MTX 20.5<br>LEF 5.4	 39.1	 20.2	 8.0	 6.3		 1.0	 1.4	
	JAK inhibitors<br>Tofacitinib<br>Baricitinib	RA	8481<br>3416<br>5065	59.3 (13.3)	78.3	N/A	41.1	MTX 11.3<br>LEF 5.2	67.0	22.3	10.8	7.8		2.0	2.9	

© 2025 Solitano V et al. *JAMA Network Open*.

Kochar[27] 2021	TNF antagonists	IBD	19,096	39.2 (17.0)	51	1.9 (2.1)	N/A	N/A	0.8						1.1	
	JAK inhibitors<br>Tofacitinib	IBD	305	43.8 (15.9)	56.4	2.8 (2.7)	N/A	N/A	32.1						2.6	
Ma[28] 2024	TNF antagonists<br>Adalimumab	RA	5582	48.7±14.1	71.1	N/A	54.2	MTX 39.0<br>LEF 8.6<br>Aza 1.3<br>Cyc 0.8	N/A	11.1	3.8		7.4	1.0		
	JAK inhibitors<br>Tofacitinib	RA	1998	51.7 ±13.7	78.5	N/A	54.0	MTX 32.3<br>LEF 10.2<br>Aza 1.9<br>Cyc 1.2	N/A	11.8	4.3		6.6	1.2		
Khosrow-Khavar[29] 2022	TNF antagonists	RA	Optum 24688<br><br>MarketScan 28727<br><br>Medicare 35816	57.1 (13.2)<br><br>55.0 (12.0)<br><br>72.2 (5.6)	81.2<br><br>81.8<br><br>83.3	N/A	50.5<br><br>49.7<br><br>56.0	MTX 45.5<br>LEF 21.4<br><br>MTX 50.8<br>LEF 20.0<br><br>MTX 50.9	N/A	53.0<br><br>45.0<br><br>82.1	21.7<br><br>15.9<br><br>30.5	43.4<br><br>38.1<br><br>67.5	23.8<br><br>15.3<br><br>15.2		5.4<br><br>3.3<br><br>11.9	

© 2025 Solitano V et al. *JAMA Network Open*.

								LEF<br>22.2								
	JAK inhibitors<br>Tofacitinib	RA	Optum<br>3761<br><br>MarketScan<br>5298<br><br>Medicare<br>3782	56.8<br>(12.5)<br><br>54.7<br>(11.5)<br><br>72.1 (5.6)	80.9<br><br>81.8<br><br>82.9	N/A	50.5<br><br>49.5<br><br>55.9	MTX<br>46.0<br>LEF<br>21.2<br><br>MTX<br>51.4<br>LEF<br>20.1<br><br>MTX<br>51.7<br>LEF<br>21.9	N/A	52.3<br><br>44.5<br><br>82.2	21.4<br><br>15.8<br><br>30.7	43.0<br><br>37.8<br><br>67.9	23.5<br><br>15.3<br><br>15.4		5.1<br><br>3.3<br><br>11.9	
Meissner[30]<br>2023	TNF<br>antagonists	RA	3694	57.7±13.3	74.0	9.6±8.7	50.6	N/A	N/A	41.5	11.6	9.1	28.0			
	JAK inhibitors	RA	3058	60.2±11.8	76.4	12.5±9.5	52.2	N/A	N/A	47.6	13.9	12.8	28.4			
Mok[31] 2024	TNF<br>antagonists	RA	1920	52.6<br>(12.6)	84.3	7.4 (6.5)	52.4	MTX<br>79.5<br>LEF<br>18.7	N/A	23.0	5.8	8.3				
	JAK inhibitors	RA	551	57.9<br>(11.4)	81.9	10.6 (7.6)	47.4	MTX<br>59.2<br>LEF<br>13.2	N/A	33.0	9.6	20.7				
Molander[32]<br>2022	TNF<br>antagonists	RA	15090	57 (47–<br>67)	77	7 (3–15)	70	N/A	N/A		9			1		
	JAK inhibitors	RA	2150	60 (51–<br>70)	82	13 (7–23)	73	N/A	N/A		10			2		

© 2025 Solitano V et al. *JAMA Network Open*.

Ozen[33] 2021	TNF antagonists	RA	7724	N/A												
	JAK inhibitors<br>Tofacitinib	RA	301	N/A												
Pawar[34] 2020	TNF antagonists<br>Adalimumab	RA	Optum<br>10205	55 (13·4)	77.7	N/A	60.7	MTX<br>63.8	N/A	51.1	18.8	30.0		6.4	2.9	
			MarketScan<br>20752	52 (12·5)	77.2		58.0	LEF<br>12.2		45.0	14.3	23.4		3.2	1.5	
			Medicare<br>5320	72 (6·3)	80.7		65.7	MTX<br>66.5		80.9	28.9	54.3		12.1	7.6	
	Etanercept		Optum<br>8128	55 (14·1)	77.8		60.8	LEF<br>10.9		52.3	19.2	30.7		6.3	2.9	
			MarketScan<br>20826	52 (12·9)	77.1		58.0	MTX<br>70.6		45.1	14.4	23.4		3.2	1.5	
			Medicare<br>7007	72 (6·4)	80.4		66.7	LEF<br>14.8		80.9	29.1	54.7		11.9	8.3	
	Golimumab		Optum<br>1911	54 (14)	77.0		60.9	MTX<br>64.1		49.8	18.3	29.8		6.1	2.7	
			MarketScan<br>2508	51 (13·5)	76.7		57.4	LEF<br>12.8		43.8	14.0	23.4		3.2	1.5	
			Medicare<br>2089	72 (6)	82.8		62.9	MTX<br>66.8		81.5	29.5	52.4		12.4	8.1	
	Certolizumab		Optum<br>2137	54 (13·9)	77.0		61.1	LEF<br>11.0		50.4	18.2	29.7		5.8	2.7	
			MarketScan<br>2277	52 (14),	77.2		57.7	MTX<br>71.6		44.4	14	23.1		3.2	1.6	
			Medicare<br>3852	72 (6·2)	80.5		66.0	LEF<br>15.3		80.6	29.0	54.8		11.9	8.4	
	Infliximab		Optum<br>2597	54 (15)	77.4		62.6	MTX<br>64.4<br>LEF<br>12.7		51.7	19.0	30.0		6.5	2.7	

© 2025 Solitano V et al. *JAMA Network Open*.

			MarketScan 6029<br>Medicare 8897	52 (13·8)<br>72 (6)	77.7<br>80.2		60.2<br>66.0	MTX 67.0<br>LEF 11.2<br>MTX 69.7<br>LEF 13.6<br><br>MTX 64.1<br>LEF 12.8<br>MTX 65.2<br>LEF 10.4<br>MTX 70.5<br>LEF 14.7<br><br>MTX 64.8<br>LEF 12.5<br>MTX 69.2<br>LEF 11.4<br>MTX 71.6<br>LEF 15.3		47.7<br>80.8	15.8<br>28.4	24.4<br>54.1		3.5<br>11.9	1.8<br>8.3	
	JAK inhibitors<br>Tofacitinib	RA	Optum	55 (12·5)	77.0	N/A	61.3		N/A	51.4	19.1	28.9		6.4	2.9	

© 2025 Solitano V et al. *JAMA Network Open*.

			1705<br>MarketScan<br>2090<br>Medicare<br>1204	53 (10·7)<br><br>72 (5·4)	77.8<br><br>81.0		59.5<br><br>65.4	MTX 66<br>LEF 13.9<br>MTX 68.5<br>LEF 12.5<br>MTX 69.9<br>LEF 15.3		48.2<br><br>80.8	14.9<br><br>28.9	24.1<br><br>54.3		3.7<br><br>13.5	1.6<br><br>8.2	
Sakai[37] 2024	bDMARD	RA	20964	63.4±15.0	74.9	N/A	30.1	MTX 67.6	N/A	19.0	8.4	12.2		3.8	9.4	
	JAK inhibitors	RA	4416	65.3±13.6	77.3	N/A	33.9	MTX 70.1	N/A	20.8	7.6	14.9		3.9	9.8	
Salinas[38] 2023	TNF antagonists	RA	HealthVerity 748<br>ARTIS 1685<br>SNDS 2859	56 (12)<br><br>59 (14)<br><br>58 (13)	85<br><br>83<br><br>81	N/A	47<br><br>62<br><br>70	MTX 28<br>LEF 14<br>MTX 43<br>LEF 6<br>MTX 52<br>LEF 11	N/A	45<br><br>0<br><br>N/A	19<br><br>7<br><br>10	37<br><br><5<br><br>N/A			0<br><br><5<br><br>0.5	
	JAK inhibitors Baricitinib	RA	HealthVerity 748<br>ARTIS 1685<br>SNDS 2859	55 (11)<br><br>59 (14)<br><br>58 (13)	86<br><br>82<br><br>79	N/A	47<br><br>64<br><br>71	MTX 29<br>LEF 12<br>MTX 43<br>LEF 4	N/A	43<br><br>1<br><br>N/A	19<br><br>8<br><br>10	36<br><br><5<br><br>N/A			1<br><br><5<br><br>0.4	

© 2025 Solitano V et al. *JAMA Network Open*.

								MTX<br>53<br>LEF<br>13								
Song[39] 2022	TNF antagonists	RA	3865	54.2±13.3	79.1	N/A	75.2	MTX<br>78.5	N/A		25.4				3.6	
	JAK inhibitors	RA	1064	55.7±12.5	82.8	N/A	78.5	MTX<br>73.0	N/A		24.9				3.9	
Song[40] 2023	TNF antagonists	RA	3307	54.2±13.2	80.5	N/A	83.0	MTX<br>81.8	N/A		25.0				3.4	
	JAK inhibitors	RA	871	54.6±12.3	83.1	N/A	81.4	MTX<br>75.3	N/A		22.3				3.2	
Song[41] 2023	bDMARD	RA	3384	48.5±12.6	70.7	N/A	93.4	MTX<br>79.3<br>LEF<br>38.7	N/A	56.7	21.2	50.7				
	JAK inhibitors	RA	846	48.1±12.7	70.6	N/A	92.9	MTX<br>78.0<br>LEF<br>38.8	N/A	57.3	22.3	51.7				
Tong[42] 2023	TNF antagonists	RA	5849	51.5 (15.6)	74.7	2.1 (2)	N/A	N/A	N/A	31.7	19.5	33.1		5.1		
	JAK inhibitors	RA	1571	53.8 (13.6)	78	3.2 (3.6)	N/A	N/A	N/A	30.9	19	34.6		4.1		
Desai[43] 2022	TNF antagonists	RA	MarketScan 39209<br><br>Medicare 23283<br><br>Optum 18387	53 (12.3)<br><br>71 (5.5)<br><br>56 (13.3)	82.1<br><br>86.8<br><br>82.8	N/A	63<br><br>67.9<br><br>62.2	N/A	N/A		15.7<br><br>26<br><br>20.8				1.6<br><br>7.2<br><br>3.9	
	JAK inhibitors	RA	MarketScan	53 (11.7)	82	N/A	63.3	N/A	N/A		15.8				1.7	

© 2025 Solitano V et al. *JAMA Network Open*.

			2992<br><br>Medicare 1795<br><br>Optum 1987	 <br>71 (5.7)<br><br>56 (12.7)	 <br>86.6<br><br>82.5		 <br>68<br><br>62.5				 <br>26.1<br><br>20.9				 <br>7.3<br><br>4.2	
Westermann[44] 2024	bDMARD	RA	4247	57.6 (13.8)	75.1	10.6 (9.7)	28.5	N/A	47.5		8.5					
	JAK inhibitors	RA	875	57.8 (13.1)	79.8	10.5 (9.1)	31.7	N/A	82.3		10.1					
Cho[45] 2025	TNF antagonists	RA	4992	54.6 (9.4)	79.1	N/A	N/A	MTX 88<br>LEF 46.1	4.5	24.9	13.1	26.5	24.3	2.7	0.3	
		UC	548	41.6 (8.6)	28.5	N/A	N/A	MTX 1<br>LEF 0	5.3	6.8	4.3	15.7	22	0.6	0	
	JAK inhibitors	RA	4992	54.6 (13.2)	79.1	N/A	N/A	MTX 88<br>LEF 46.1	4.5	24.9	13.1	26.5	23.7	2.7	0.3	
		UC	548	41.6 (18.8)	28.5	N/A	N/A	MTX 1<br>LEF 0	5.3	6.8	4.3	15.7	20.6	0.6	0	
Zhao[46] 2025	TNF antagonists	PsA or SpA	2200	51.1 (14.4)	64.9	N/A	N/A	N/A	N/A	31.8	14.5	28.4				
	JAK inhibitors	PsA or SpA	2200	50.7 (13.6)	62.9	N/A	N/A	N/A	N/A	32.7	14.4	28.6				
Aymon[47] 2025	TNF antagonists	RA	25,049	57.12 (13.34)	75.5	8.35 (3.70–16.00)	34.4	MTX 35	N/A	27.4	8.8	14.2				
	JAK inhibitors	RA	13,734	58.29 (12.22)	78.3	10.96 (5.36–18.43)	44.2	MTX 32.6	N/A	30.4	9.1	18.3				

© 2025 Solitano V et al. *JAMA Network Open*.

Silvagni[48] 2025	TNF antagonists	RA	2343	57 (47, 66)	75	N/A	46.9	MTX 44.5	N/A	25.9	6.7	8.9			0.3	
	JAK inhibitors	RA	1443	59 (51, 68)	79.1	N/A	53.8	MTX 46	N/A	29.9	7.3	10.2			0.8	
Kao[49] 2025	TNF antagonists	RA	8902	53.6 ± 13.2	76.3	4.7 ± 4.0	89.3	MTX 91.4	N/A	23.8	9.3	10			4.3	
	JAK inhibitors	RA	1462	55.7 ± 12.8	78	6.0 ± 5.2	86.4	MTX 91.4	N/A	24.1	10.9	13.1			4.1	
Tanaka[50] 2025	TNF antagonists	RA	663	60.3 (15.1)	81.1	7.2 (9.4)	30.9	MTX 75	0.9	28.2	8.1	14.3	4.6			
	JAK inhibitors	RA	253	62.1 (13.2)	82.2	11.6 (11)	29.2	MTX 64 4	1.4	29.6	10.7	20.6	6.6			

© 2025 Solitano V et al. *JAMA Network Open*.

eTable 3. Risk of Bias in the Studies Included in the Systematic Review According to the Newcastle-Ottawa Scale

	Selection				Outcome			Comparability	
**Study**	Item 1	Item 2	Item 3	Item 4	Item 5	Item 6	Item 7	Item 8	**Score**
Ahuja[9] 2025	★	★	★	★	★	★	★	★	8
Bastard[10] 2024	★	★	★	★	★	★	★	★★	9
Bower[11] 2023	★	★	★	★	★	★	★	★★	9
Huss[12] 2022	★	★	★	★	★	★	★	★★	9
Huss[13] 2023	★	★	★	★	★	★	★	★★	9
Khosrow-Khavar[14] 2022	★	★	★	★	★	★	★	★★	9
Min[15] 2023	★	★	★	★		★	★	★★	8
Uchida[16] 2023	★	★	★	★	★	★	★	★★	9
Kremer[17] 2021	★	★	★	★	★	★	★	★★	9
Bilgin[18] 2022	★	★	★	★	★		★		6
Cheng[19] 2022	★	★	★	★	★	★	★	★★	9
Cho[20] 2024	★	★	★	★	★	★	★	★★	9
Choi[21] 2023	★	★	★	★	★	★	★	★★	9

© 2025 Solitano V et al. *JAMA Network Open*.

de Avila Machado[22] 2018	★	★	★	★	★	★	★	★★	9
Fang[23] 2022	★	★	★	★	★	★	★	★★	9
Frisell[24] 2023	★	★	★	★	★	★	★	★★	9
Hernández-Cruz[25] 2024	★	★	★	★	★	★	★	★★	9
Hoisnard[26] 2022	★	★	★	★	★	★	★	★★	9
Kochar[27] 2021	★	★	★	★	★	★	★	★★	9
Ma[28] 2024	★	★	★	★	★	★	★	★★	9
Khosrow-Khavar[29] 2022	★	★	★	★	★	★	★	★★	9
Meissner[30] 2023	★	★	★	★	★	★	★	★★	9
Mok[31] 2024	★	★	★	★	★	★	★	★★	9
Molander[32] 2022	★	★	★	★		★	★	★★	8
Ozen[33] 2021	★	★	★	★	★	★	★	★	8
Pawar[34] 2020	★	★	★	★	★	★	★	★★	9
Sendaydiego[35] 2024	★	★	★	★	★		★	★★	8
Sendaydiego[36] 2024	★	★	★	★	★	★	★	★★	9
Sakai[37] 2024	★	★	★	★	★	★	★	★★	9
Salinas[38] 2023	★	★	★	★	★	★	★	★★	9

© 2025 Solitano V et al. *JAMA Network Open*.

Song[39] 2022	★	★	★	★	★	★	★	★	8
Song[40] 2023	★	★	★	★	★		★	★★	8
Song[41] 2023	★	★	★	★	★	★	★	★★	9
Tong[42] 2023	★	★	★	★	★	★	★	★★	9
Desai[43] 2021	★	★	★	★	★	★	★	★★	9
Westermann[44] 2024	★	★	★	★	★		★	★★	8
Cho[45] 2025	★	★	★	★	★	★	★	★★	9
Zhao[46] 2025		★	★	★	★	★	★	★★	8
Aymon[47] 2025	★	★	★	★	★	★	★	★★	9
Silvagni[48] 2025	★	★	★	★	★	★	★	★★	9
Kao[49] 2025	★	★	★	★	★	★	★	★★	9
Tanaka[50] 2025	★	★	★	★	★	★	★	★★	9

Items: 1, representativeness of the exposed cohort; 2, selection of the non-exposed cohort; 3, ascertainment of exposure; 4, demonstration that outcome of interest was not present at start of study; 5, assessment of outcome; 6, follow-up was long enough for outcomes to occur; 7, adequacy of follow-up (>75% follow-up, or description for those lost); 8, comparability of cohorts on the basis of the design or analysis.

© 2025 Solitano V et al. *JAMA Network Open*.

eTable 4. Meta-Regression Analyses for All Outcomes of Interest

	**Serious infections**	**Malignancy**	**MACE**	**VTE**
Concomitant steroid	0.68	0.37	0.70	0.71
Concomitant immunomodulators	**0.045**	0.83	0.32	0.89
Prior infections	0.51	-	-	-
Diabetes	0.45	0.80	0.92	0.28
Mean age	0.51	0.86	0.75	0.08
Proportion of males	**0.01**	0.71	0.44	0.26
Midpoint of recruitment	0.83	0.38	0.39	0.83
Smoker	0.51	0.53	0.18	0.06
Hypertension	0.64	0.92	0.88	0.67
Hyperlipidemia	0.71	0.79	0.99	0.73
Prior CAD	0.99	0.92	0.39	0.33
Obesity	0.48	0.52	**0.02**	**0.05**

© 2025 Solitano V et al. *JAMA Network Open*.

eFigure 1. PRISMA Flow Diagram

PRISMA 2020 flow diagram for new systematic reviews which included searches of databases, registers and other sources

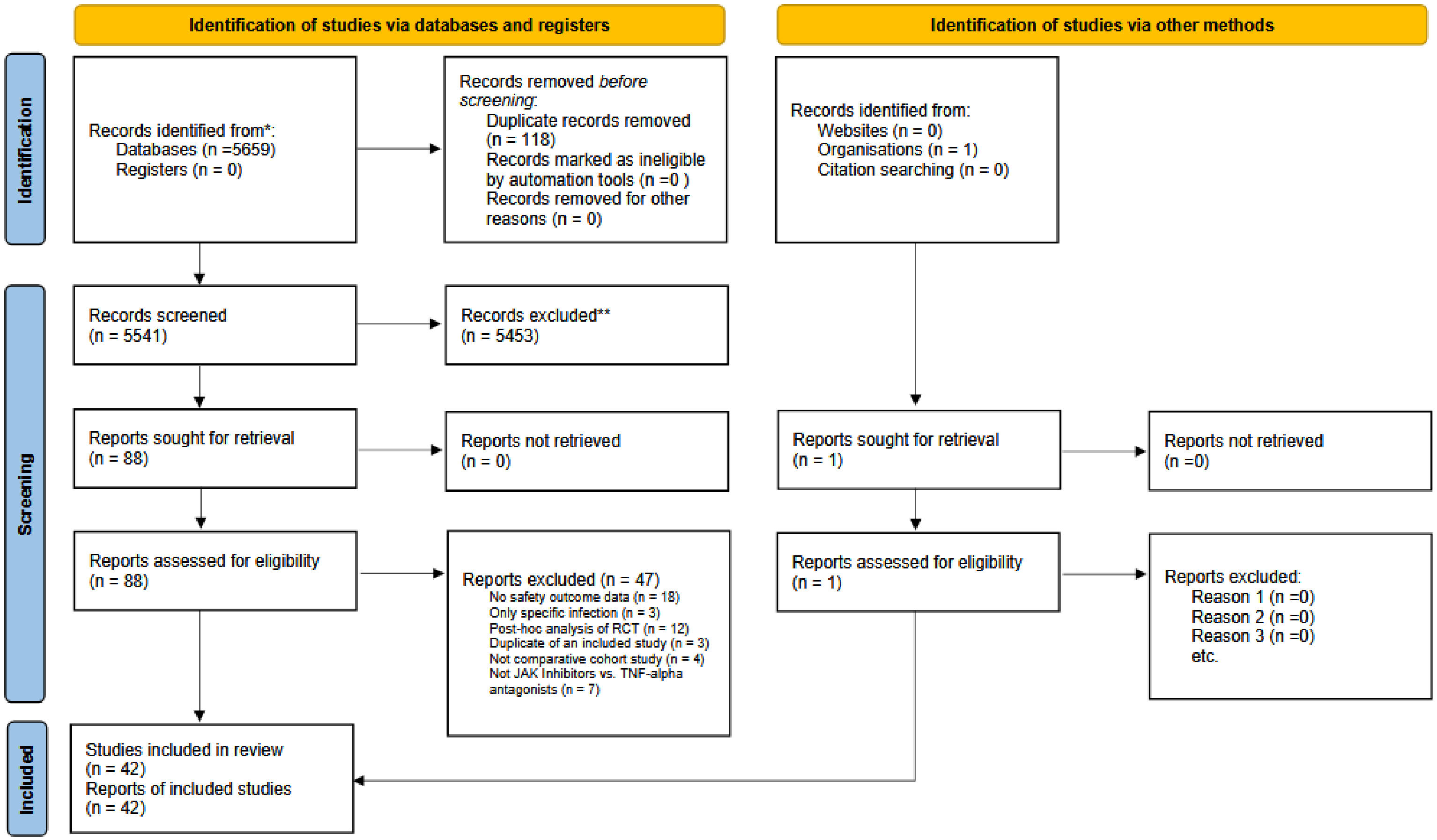

*Consider, if feasible to do so, reporting the number of records identified from each database or register searched (rather than the total number across all databases/registers).
**If automation tools were used, indicate how many records were excluded by a human and how many were excluded by automation tools.

Source: Page MJ, et al. BMJ 2021;372:n71. doi: 10.1136/bmj.n71.

This work is licensed under CC BY 4.0. To view a copy of this license, visit https://creativecommons.org/licenses/by/4.0/

eFigure 2. Risk of Serious Infections With JAK Inhibitors vs TNF Antagonists in All Patients With Immune-Mediated Inflammatory Diseases (Subgroup Analysis Based on Location of Study)

Author	Counts	Total	IR	Counts	Total	IR	HR	95%-CI
**North America**								
Ahuja, 2025	58	856	4.85	891	9,422	5.37	0.97	[0.66; 1.44]
Salinas (USA), 2023	15	1,913	2.85	24	1,913	2.75	1.18	[0.74; 1.88]
Cheng, 2022	17	305	8.99	1,407	19,096	7.35	0.59	[0.27; 1.05]
Kremer, 2021	90	1,999	3.12	333	8,358	2.83	0.99	[0.75; 1.30]
Pawar (Optum), 2020	49	2,009	3.14	438	24,968	2.56	1.14	[0.94; 1.39]
Pawar (MarketScan), 2020	58	2,755	2.3	759	52,741	1.77	1.09	[0.89; 1.32]
Pawar (Medicare), 2020	72	1,515	6.64	1,179	27,423	6.33	1.05	[0.91; 1.22]
de Ávila Machado, 2018	17	164	3.67	490	13,367	2.16	1.66	[1.03; 2.70]
**Random effects model (HK)**							**1.08**	**[0.96; 1.20]**
Heterogeneity: $I^2 = 4.8\%$								
**Europe**								
Bastard, 2024	5	152	3.48	150	4,616	1.89	1.07	[0.33; 3.44]
Hernandez-Cruz (RA), 2024	156	1,386	4.93	199	2,861	3.09	1.10	[0.90; 1.40]
Hernandez-Cruz (SpA), 2024	7	256	1.86	194	3,218	1.88	0.70	[0.30; 1.40]
Frisell, 2023	130	2,263	3.29	240	8,748	3.08	0.97	[0.75; 1.24]
Salinas (Europe), 2023	147	5,530	3.38	114	5,530	2.23	1.43	[1.12; 1.83]
**Random effects model (HK)**							**1.11**	**[0.85; 1.46]**
Heterogeneity: $I^2 = 38.5\%$								
**Asia**								
Cho (RA), 2025	449	4,992	4.8	868	4,992	3.7	1.08	[0.94; 1.23]
Cho (UC), 2025	14	548	2.6	166	548	6.8	0.45	[0.26; 0.78]
Tanaka, 2025	14	253	2.46	27	663	2.63	1.34	[0.70; 2.56]
Uchida, 2023	40	296	8.36	16	203	4.07	0.78	[0.41; 1.49]
Choi, 2023	48	2,963	1.39	61	5,169	1.32	1.04	[0.71; 1.52]
Mok, 2023	92	551	8.24	314	1,920	5.91	0.97	[0.76; 1.23]
Salinas (Asia), 2023	9	384	4.7	7	384	2.29	1.83	[0.65; 5.14]
**Random effects model (HK)**							**0.97**	**[0.72; 1.30]**
Heterogeneity: $I^2 = 49.9\%$								
**Random effects model (HK)**							**1.07**	**[0.99; 1.16]**
Heterogeneity: $I^2 = 30.1\%$								
Test for subgroup differences: $\chi^2_2 = 0.85$, df = 2 ($p = 0.6550$)								

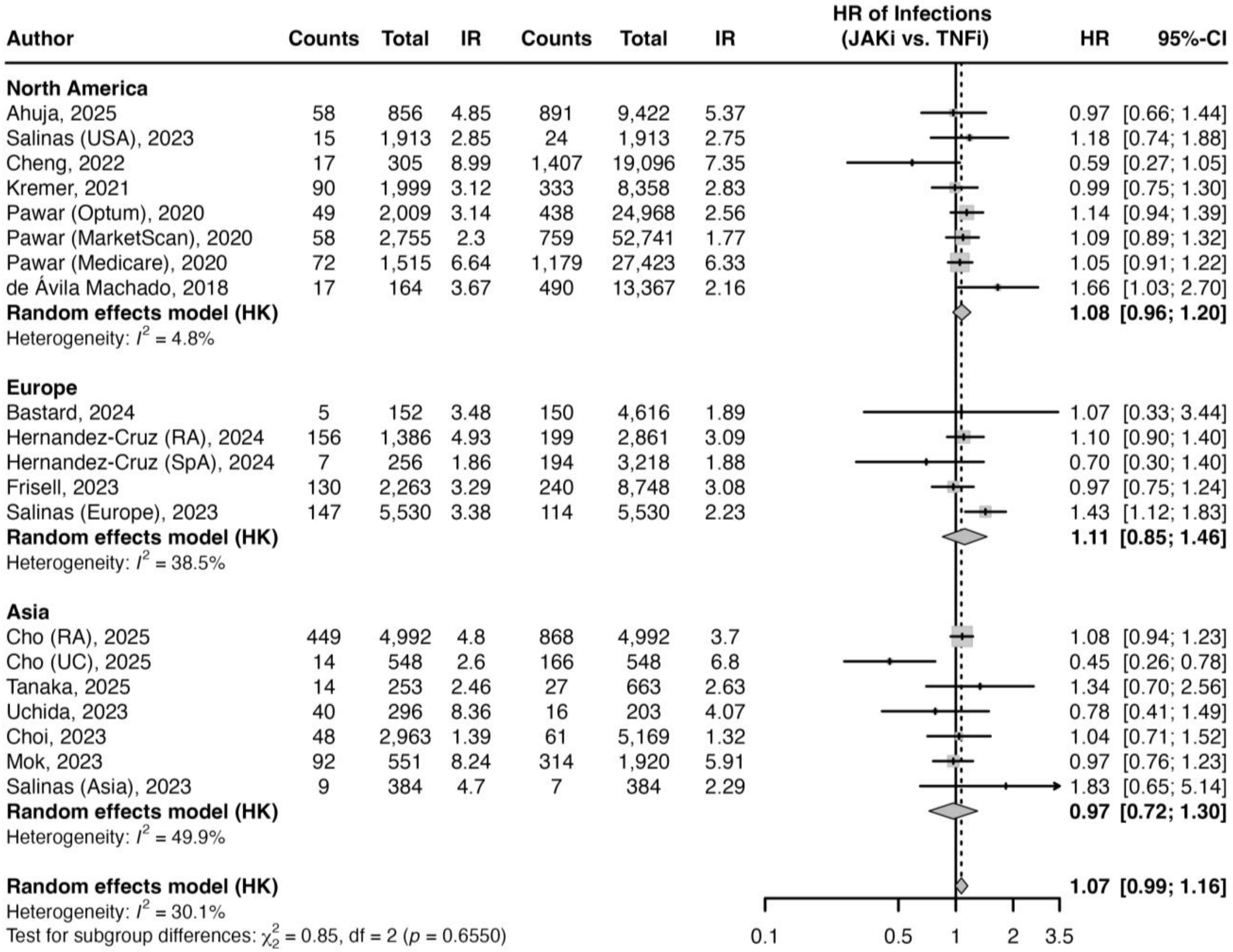

© 2025 Solitano V et al. *JAMA Network Open*.

eFigure 3. Risk of Serious Infections With JAK inhibitors vs TNF Antagonists in All Patients With Immune-Mediated Inflammatory Diseases (Subgroup Analysis Based on Type of Immune-Mediated Inflammatory Disease)

Author	Counts	Total	IR	Counts	Total	IR	HR	95%-CI
**RA**								
Cho (RA), 2025	449	4,992	4.8	868	4,992	3.7	1.08	[0.94; 1.23]
Tanaka, 2025	14	253	2.46	27	663	2.63	1.34	[0.70; 2.56]
Hernandez-Cruz (RA), 2024	156	1,386	4.93	199	2,861	3.09	1.10	[0.90; 1.40]
Frisell, 2023	130	2,263	3.29	240	8,748	3.08	0.97	[0.75; 1.24]
Uchida, 2023	40	296	8.36	16	203	4.07	0.78	[0.41; 1.49]
Choi, 2023	48	2,963	1.39	61	5,169	1.32	1.04	[0.71; 1.52]
Mok, 2023	92	551	8.24	314	1,920	5.91	0.97	[0.76; 1.23]
Salinas, 2023	176	7,606	2.96	145	7,606	2.19	1.36	[0.86; 2.13]
Kremer, 2021	90	1,999	3.12	333	8,358	2.83	0.99	[0.75; 1.30]
Pawar (Optum), 2020	49	2,009	3.14	438	24,968	2.56	1.14	[0.94; 1.39]
Pawar (MarketScan), 2020	58	2,755	2.3	759	52,741	1.77	1.09	[0.89; 1.32]
Pawar (Medicare), 2020	72	1,515	6.64	1,179	27,423	6.33	1.05	[0.91; 1.22]
de Ávila Machado, 2018	17	164	3.67	490	13,367	2.16	1.66	[1.03; 2.70]
**Random effects model (HK)**							**1.07**	**[1.01; 1.14]**
Heterogeneity: $I^2 = 0\%$								
**SpA**								
Hernandez-Cruz (SpA), 2024	7	256	1.86	194	3,218	1.88	0.70	[0.30; 1.40]
**PsA**								
Bastard, 2024	5	152	3.48	150	4,616	1.89	1.07	[0.33; 3.44]
**IBD**								
Ahuja, 2025	58	856	4.85	891	9,422	5.37	0.97	[0.66; 1.44]
Cho (UC), 2025	14	548	2.6	166	548	6.8	0.45	[0.26; 0.78]
Cheng, 2022	17	305	8.99	1,407	19,096	7.35	0.59	[0.27; 1.05]
**Random effects model (HK)**							**0.66**	**[0.24; 1.83]**
Heterogeneity: $I^2 = 62.9\%$								
**Random effects model (HK)**							**1.05**	**[0.97; 1.13]**
Heterogeneity: $I^2 = 20.4\%$								
Test for subgroup differences: $\chi^2_3 = 5.29$, df = 3 ($p = 0.1520$)								

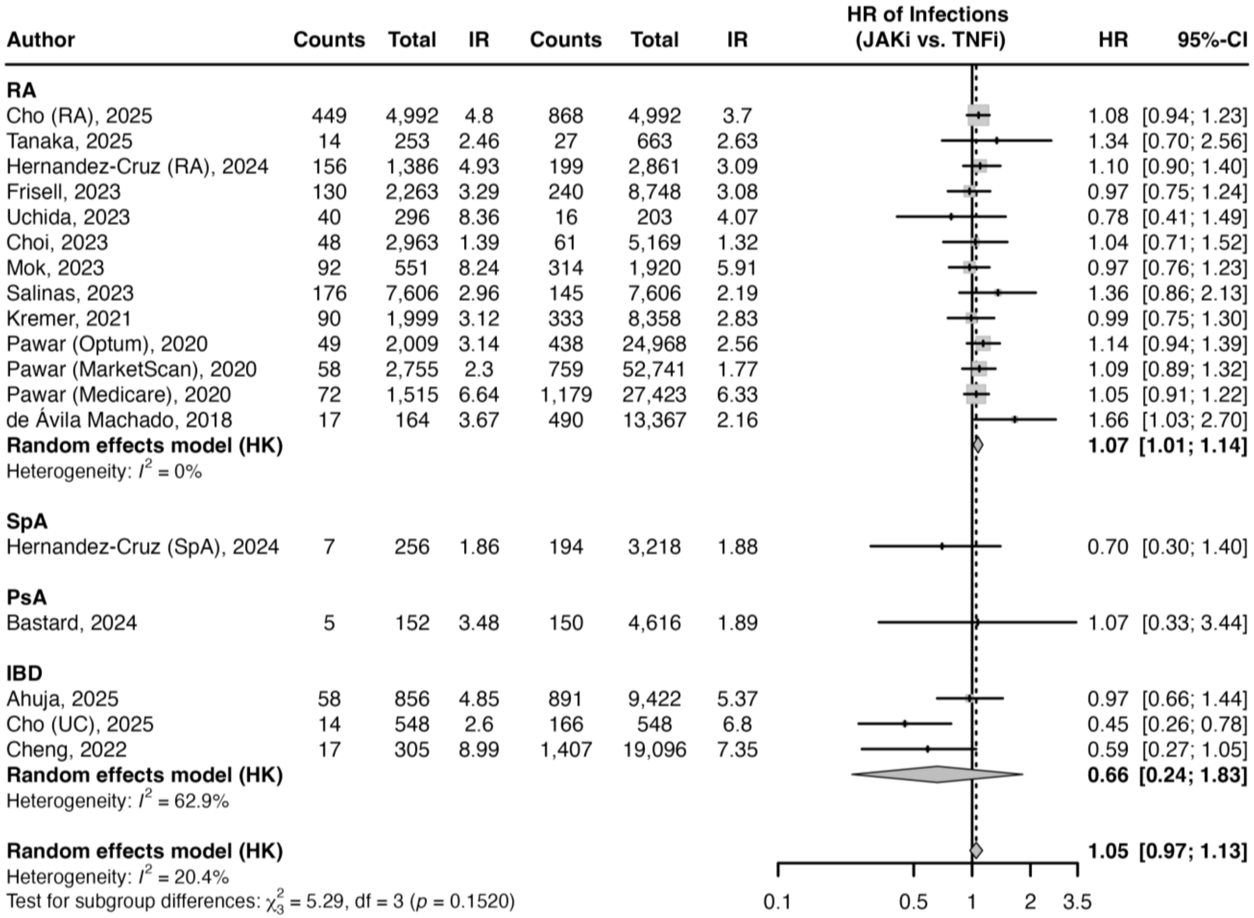

© 2025 Solitano V et al. *JAMA Network Open*.

eFigure 4. Risk of Serious Infections With JAK Inhibitors vs TNF Antagonists in All Patients With Immune-Mediated Inflammatory Diseases (Subgroup Analysis Based on Type of JAK Inhibitor)

Author	Counts	Total	IR	Counts	Total	IR	HR of Infections (JAKi vs. TNFi)	HR	95%-CI
**Tofacitinib**									
Ahuja, 2025	42	659	4.71	811	9,422	5.37		1.00	[0.64; 1.55]
Cho (RA), 2025	209	2,019	4.4	868	4,992	3.75		1.05	[0.89; 1.24]
Tanaka, 2025	14	253	2.46	27	663	2.63		1.34	[0.70; 2.56]
Bastard, 2024	5	152	3.48	150	4,616	1.89		1.07	[0.33; 3.44]
Frisell (Tofacitinib), 2023	25	426	3.05	571	8,748	3.08		0.89	[0.47; 1.69]
Uchida (Tofacitinib), 2023	33	192	8.68	16	203	4.07		1.53	[0.84; 2.77]
Cheng, 2022	17	305	8.99	1,407	19,096	7.35		0.59	[0.27; 1.05]
Kremer, 2021	90	1,999	3.12	333	8,358	2.83		0.99	[0.75; 1.30]
Pawar (Optum), 2020	49	2,009	3.14	438	24,968	2.56		1.14	[0.94; 1.39]
Pawar (MarketScan), 2020	58	2,755	2.3	759	52,741	1.77		1.09	[0.89; 1.32]
Pawar (Medicare), 2020	72	1,515	6.64	1,179	27,423	6.33		1.05	[0.91; 1.22]
de Ávila Machado, 2018	17	164	3.67	490	13,367	2.16		1.66	[1.03; 2.70]
**Random effects model (HK)**								**1.08**	**[0.99; 1.16]**
Heterogeneity: $I^2 = 0\%$									
**Baricitinib**									
Cho (RA), 2025	181	1,858	5	868	4,992	3.75		1.03	[0.83; 1.27]
Salinas, 2023	176	7,606	2.96	145	7,606	2.19		1.36	[0.86; 2.13]
Frisell (Baricitinib), 2023	105	1,837	3.36	571	8,748	3.08		0.98	[0.75; 1.29]
Uchida (Baricitinib), 2023	7	104	7.09	16	203	4.07		1.49	[0.61; 3.63]
**Random effects model (HK)**								**1.06**	**[0.86; 1.31]**
Heterogeneity: $I^2 = 0\%$									
**Upadacitinib**									
Ahuja, 2025	12	197	5.39	811	9,422	5.37		0.93	[0.37; 2.34]
Cho (RA), 2025	59	1,115	5.5	868	4,992	3.75		1.12	[0.80; 1.56]
**Random effects model (HK)**								**1.10**	**[0.51; 2.33]**
Heterogeneity: $I^2 = 0\%$									
**Random effects model (HK)**								**1.07**	**[1.01; 1.14]**
Heterogeneity: $I^2 = 0.0\%$									
Test for subgroup differences: $\chi^2_2 = 0.16$, df = 2 ($p = 0.9242$)									

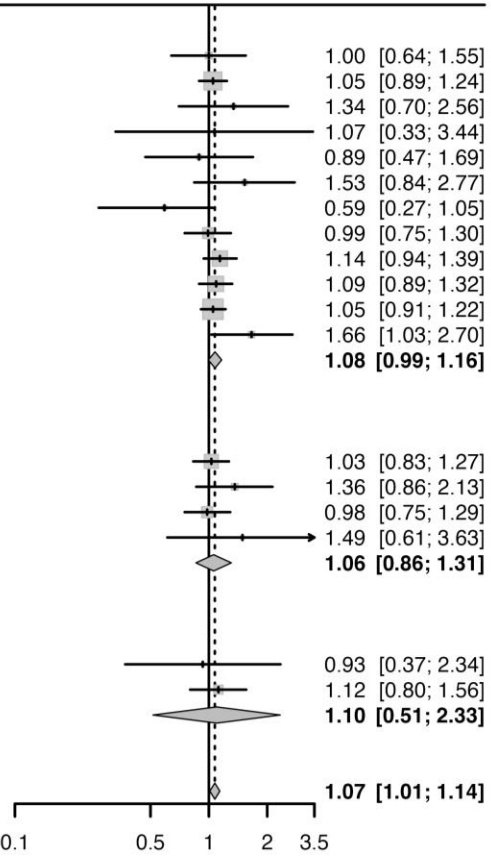

© 2025 Solitano V et al. *JAMA Network Open*.

eFigure 5. Funnel Plots for Publication Bias for Serious Infection Outcome

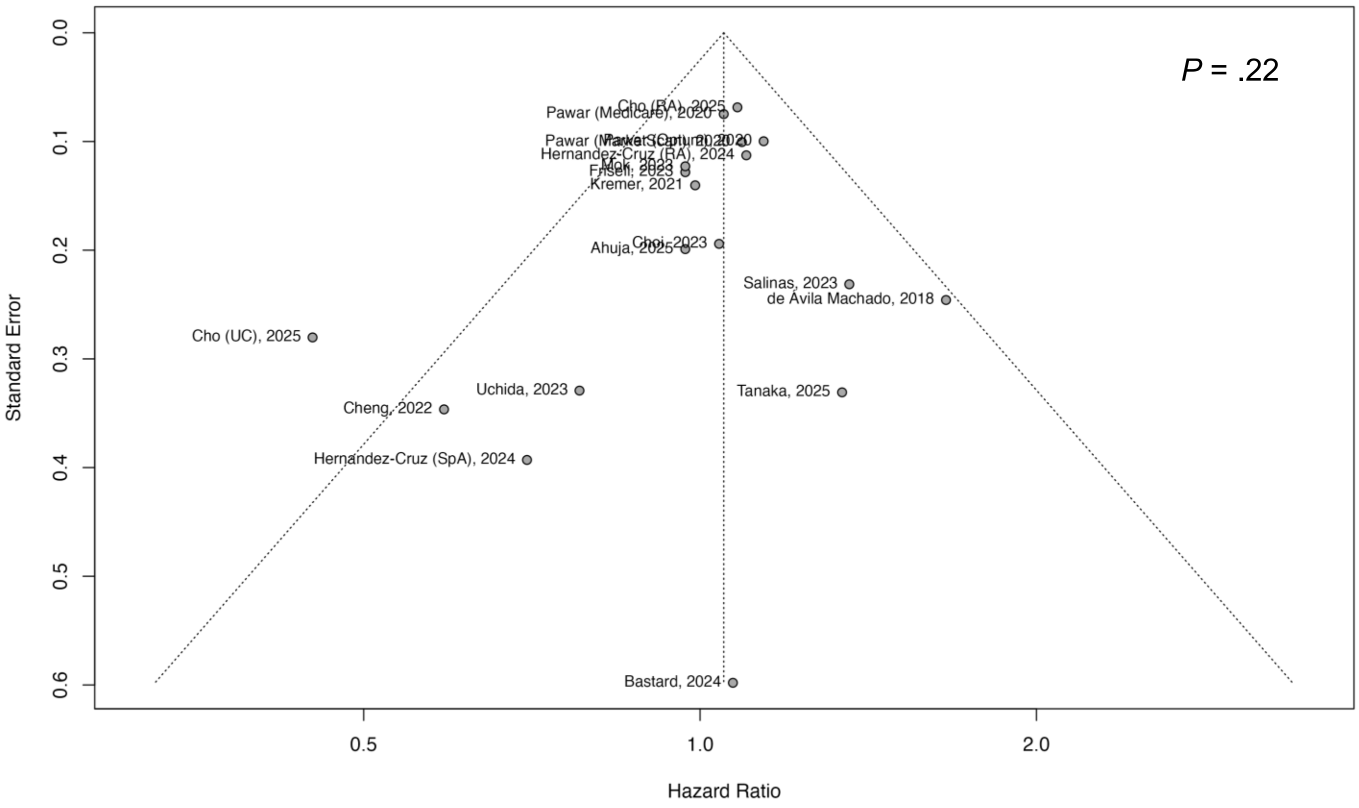

© 2025 Solitano V et al. *JAMA Network Open*.

eFigure 6. Risk of Malignant Neoplasms With JAK Inhibitors vs TNF Antagonists in All Patients With Immune-Mediated Inflammatory Diseases (Subgroup Analysis Based on Location of Study)

Author	Counts	Total	IR	Counts	Total	IR	HR	95%-CI
**North America**								
Sendaydiego, 2024	22	2,570	0.94	162	20,586	0.78	1.40	[0.90; 2.00]
Khosrow-Khavar (Optum), 2022	48	3,304	1.68	284	22,106	1.36	1.14	[0.82; 1.59]
Khosrow-Khavar (MarketScan), 2022	24	4,508	0.6	203	25,003	0.86	0.65	[0.42; 1.02]
Khosrow-Khavar (Medicare), 2022	62	2,692	2.7	546	25,682	2.49	1.09	[0.83; 1.45]
Kremer, 2021	43	1,999	1.01	119	8,538	0.76	1.34	[0.94; 1.93]
**Random effects model (HK)**							**1.11**	**[0.78; 1.56]**
Heterogeneity: $I^2 = 49.9\%$								
**Europe**								
Hernandez-Cruz (RA), 2024	63	1,386	1.99	128	3,513	1.99	0.90	[0.60; 1.20]
Hernandez-Cruz (SpA), 2024	4	256	1.06	138	3,313	1.34	0.90	[0.30; 2.50]
Westermann, 2024	19	875	1.44	92	3,758	1.31	1.19	[0.64; 2.11]
Huss (RA), 2023	38	2,143	0.83	213	8,580	1.01	0.94	[0.65; 1.38]
Huss (PsA), 2023	5	379	0.73	73	4,186	0.58	1.88	[0.68; 5.16]
Bilgin, 2022	3	145	0.85	1	114	0.24	3.50	[0.40; 92.20]
**Random effects model (HK)**							**0.99**	**[0.79; 1.25]**
Heterogeneity: $I^2 = 0\%$								
**Asia**								
Cho (RA), 2025	53	4,992	0.54	90	4,992	0.37	1.61	[1.08; 2.41]
Cho (UC), 2025	1	548	0.18	5	548	0.19	0.98	[0.11; 8.42]
Cho, 2024	1	200	0.52	7	455	1.69	0.31	[0.04; 2.57]
Min, 2023	81	2,498	1.27	648	9,267	1.24	0.94	[0.74; 1.19]
Uchida, 2023	11	296	1.61	4	203	0.94	0.38	[0.09; 1.55]
Mok, 2023	9	551	0.81	45	1,920	0.85	0.87	[0.39; 1.95]
Fang, 2022	4	822	0.26	22	2,357	0.44	1.10	[0.44; 2.78]
Song, 2022	7	1,064	0.54	58	3,865	0.85	0.69	[0.30; 1.56]
**Random effects model (HK)**							**0.98**	**[0.70; 1.39]**
Heterogeneity: $I^2 = 26.8\%$								
**Random effects model (HK)**							**1.06**	**[0.92; 1.21]**
Heterogeneity: $I^2 = 16.7\%$								
Test for subgroup differences: $\chi^2_2 = 0.57$, df = 2 ($p = 0.7513$)								

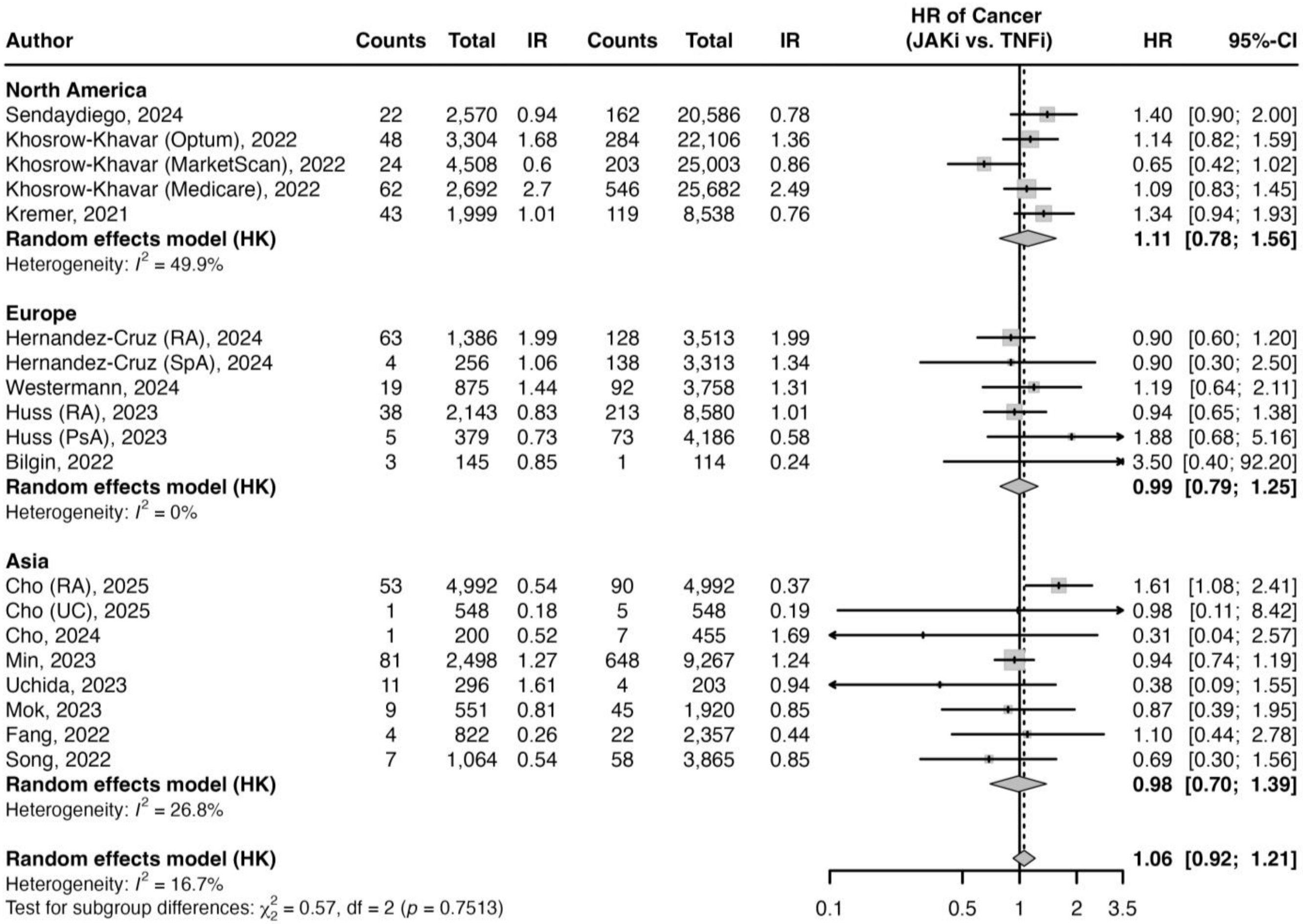

© 2025 Solitano V et al. *JAMA Network Open*.

eFigure 7. Risk of Malignant Neoplasms With JAK Inhibitors vs TNF Antagonists in All Patients With Immune-Mediated Inflammatory Diseases (Subgroup Analysis Based on Subtype of Malignant Neoplasms)

Author	Counts	Total	IR	Counts	Total	IR	HR	95%-CI
**Solid organ cancers**								
Huss (RA), 2023	14	2,143	0.17	72	8,580	0.16	0.98	[0.56; 1.71]
Song, 2022	6	1,064	0.47	56	3,865	0.82	0.61	[0.26; 1.47]
Khosrow-Khavar (Optum), 2022	28	3,304	0.19	156	22,106	0.13	1.22	[0.78; 1.90]
Khosrow-Khavar (MarketScan), 2022	13	4,508	0.06	100	25,003	0.09	0.73	[0.39; 1.35]
Khosrow-Khavar (Medicare), 2022	26	2,692	0.29	312	25,682	0.29	0.89	[0.59; 1.33]
Min- No solid organ/hemat/skin cancer, NA	NA	NA	--	NA	NA	--		
**Random effects model (HK)**							**0.93**	**[0.70; 1.25]**
Heterogeneity: $I^2 = 0\%$								
**Hematological cancers**								
Huss (RA), 2023	6	2,143	0.15	22	8,580	0.1	1.90	[0.70; 5.16]
Song, 2022	1	1,064	0.08	2	3,865	0.03	2.41	[0.15; 37.99]
Khosrow-Khavar (Optum), 2022	2	3,304	0.07	25	22,106	0.12	0.44	[0.10; 1.97]
Khosrow-Khavar (MarketScan), 2022	5	4,508	0.13	28	25,003	0.12	1.33	[0.49; 3.61]
Khosrow-Khavar (Medicare), 2022	9	2,692	0.39	93	25,682	0.42	0.88	[0.43; 1.84]
Min- No solid organ/hemat/skin cancer, NA	NA	NA	--	NA	NA	--		
**Random effects model (HK)**							**1.10**	**[0.59; 2.04]**
Heterogeneity: $I^2 = 0\%$								
**Random effects model (HK)**							**0.96**	**[0.78; 1.19]**
Heterogeneity: $I^2 = 0.0\%$								
Test for subgroup differences: $\chi^2_1 = 0.45$, df = 1 ($p = 0.5023$)								

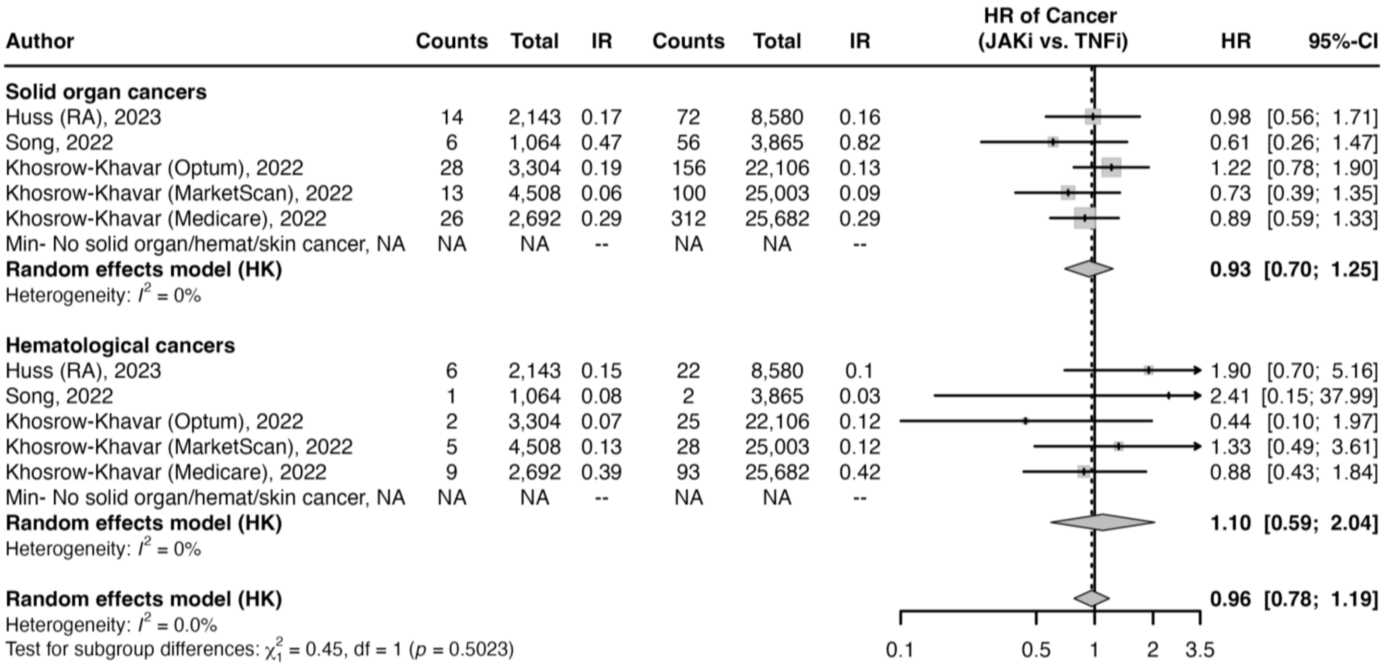

© 2025 Solitano V et al. *JAMA Network Open*.

eFigure 8. Risk of Malignant Neoplasms With JAK Inhibitors vs TNF Antagonists in All Patients With Immune-Mediated Inflammatory Diseases (Subgroup Analysis Based on Type of JAK Inhibitor)

Author	Counts	Total	IR	Counts	Total	IR	HR of Cancer (JAKi vs. TNFi)	HR	95%-CI
**Tofacitinib**									
Cho (RA), 2025	34	2,019	0.71	90	4,992	3.95		1.60	[1.03; 2.50]
Tanaka, 2025	11	253	1.54	28	663	2.14		0.99	[0.49; 2.00]
Cho, 2024	1	200	0.52	7	455	1.69		0.31	[0.04; 2.57]
Huss (RA- Tofacitinib), 2023	8	377	1.12	213	8,580	1.01		1.08	[0.52; 2.24]
Uchida (Tofacitinib), 2023	9	192	1.59	4	203	0.94		1.67	[0.51; 5.41]
Bilgin, 2022	3	145	0.85	1	114	0.24		3.50	[0.40; 92.20]
Fang, 2022	4	822	0.26	22	2,357	0.44		1.10	[0.44; 2.78]
Khosrow-Khavar (Optum), 2022	48	3,304	1.68	284	22,106	1.36		1.14	[0.82; 1.59]
Khosrow-Khavar (MarketScan), 2022	24	4,508	0.6	203	25,003	0.86		0.65	[0.42; 1.02]
Khosrow-Khavar (Medicare), 2022	62	2,692	2.7	546	25,682	2.49		1.09	[0.83; 1.45]
Kremer, 2021	43	1,999	1.01	119	8,538	0.76		1.34	[0.94; 1.93]
**Random effects model (HK)**								**1.12**	**[0.91; 1.37]**
Heterogeneity: $I^2 = 16.5\%$									
**Baricitinib**									
Cho (RA), 2025	16	1,858	0.45	90	4,992	3.95		1.74	[0.79; 3.85]
Huss (RA- Baricitinib), 2023	30	1,676	0.8	213	8,580	1.01		0.92	[0.61; 1.38]
Uchida (Baricitinib), 2023	2	104	1.71	4	203	0.94		1.71	[0.31; 9.32]
**Random effects model (HK)**								**1.18**	**[0.46; 3.01]**
Heterogeneity: $I^2 = 11.7\%$									
**Upadacitinib**									
Cho (RA), 2025	3	1,115	0.35	90	4,992	3.95		1.84	[0.35; 9.73]
**Random effects model (HK)**								**1.12**	**[0.95; 1.32]**
Heterogeneity: $I^2 = 4.3\%$									
Test for subgroup differences: $\chi^2_2 = 0.38$, df = 2 ($p = 0.8274$)									

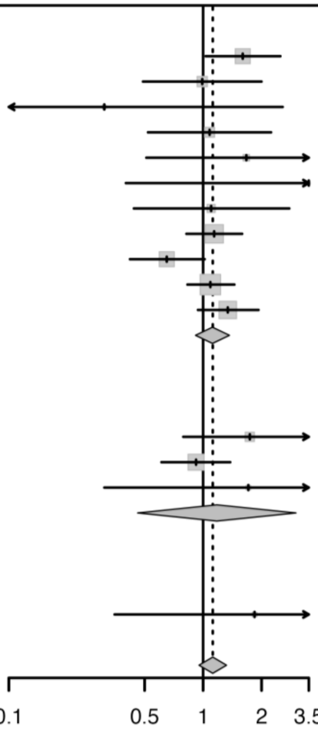

© 2025 Solitano V et al. *JAMA Network Open*.

eFigure 9. Risk of Malignant Neoplasms With JAK Inhibitors vs TNF Antagonists in All Patients With Immune-Mediated Inflammatory Diseases (IMIDs) (Subgroup Analysis Based on Type of IMID)

Author	Counts	Total	IR	Counts	Total	IR	HR	95%-CI
**RA**								
Cho (RA), 2025	53	4,992	0.54	90	4,992	0.37	1.61	[1.08; 2.41]
Tanaka, 2025	11	253	1.54	28	663	2.14	0.99	[0.49; 2.00]
Cho, 2024	1	200	0.52	7	455	1.69	0.31	[0.04; 2.57]
Hernandez-Cruz (RA), 2024	63	1,386	1.99	128	3,513	1.99	0.90	[0.60; 1.20]
Westermann, 2024	19	875	1.44	92	3,758	1.31	1.19	[0.64; 2.11]
Sendaydiego, 2024	22	2,570	0.94	162	20,586	0.78	1.40	[0.90; 2.00]
Huss (RA), 2023	38	2,143	0.83	213	8,580	1.01	0.94	[0.65; 1.38]
Min, 2023	81	2,498	1.27	648	9,267	1.24	0.94	[0.74; 1.19]
Uchida, 2023	11	296	1.61	4	203	0.94	0.38	[0.09; 1.55]
Mok, 2023	9	551	0.81	45	1,920	0.85	0.87	[0.39; 1.95]
Bilgin, 2022	3	145	0.85	1	114	0.24	3.50	[0.40; 92.20]
Fang, 2022	4	822	0.26	22	2,357	0.44	1.10	[0.44; 2.78]
Song, 2022	7	1,064	0.54	58	3,865	0.85	0.69	[0.30; 1.56]
Khosrow-Khavar (Optum), 2022	48	3,304	1.68	284	22,106	1.36	1.14	[0.82; 1.59]
Khosrow-Khavar (MarketScan), 2022	24	4,508	0.6	203	25,003	0.86	0.65	[0.42; 1.02]
Khosrow-Khavar (Medicare), 2022	62	2,692	2.7	546	25,682	2.49	1.09	[0.83; 1.45]
Kremer, 2021	43	1,999	1.01	119	8,538	0.76	1.34	[0.94; 1.93]
**Random effects model (HK)**							**1.05**	**[0.91; 1.20]**
Heterogeneity: $I^2 = 21.1\%$								
**SpA**								
Hernandez-Cruz (SpA), 2024	4	256	1.06	138	3,313	1.34	0.90	[0.30; 2.50]
**PsA**								
Huss (PsA), 2023	5	379	0.73	73	4,186	0.58	1.88	[0.68; 5.16]
**IBD**								
Cho (UC), 2025	1	548	0.18	5	548	0.19	0.98	[0.11; 8.42]
**Random effects model (HK)**							**1.05**	**[0.93; 1.20]**
Heterogeneity: $I^2 = 12.2\%$								
Test for subgroup differences: $\chi^2_3 = 1.35$, df = 3 ($p = 0.7175$)								

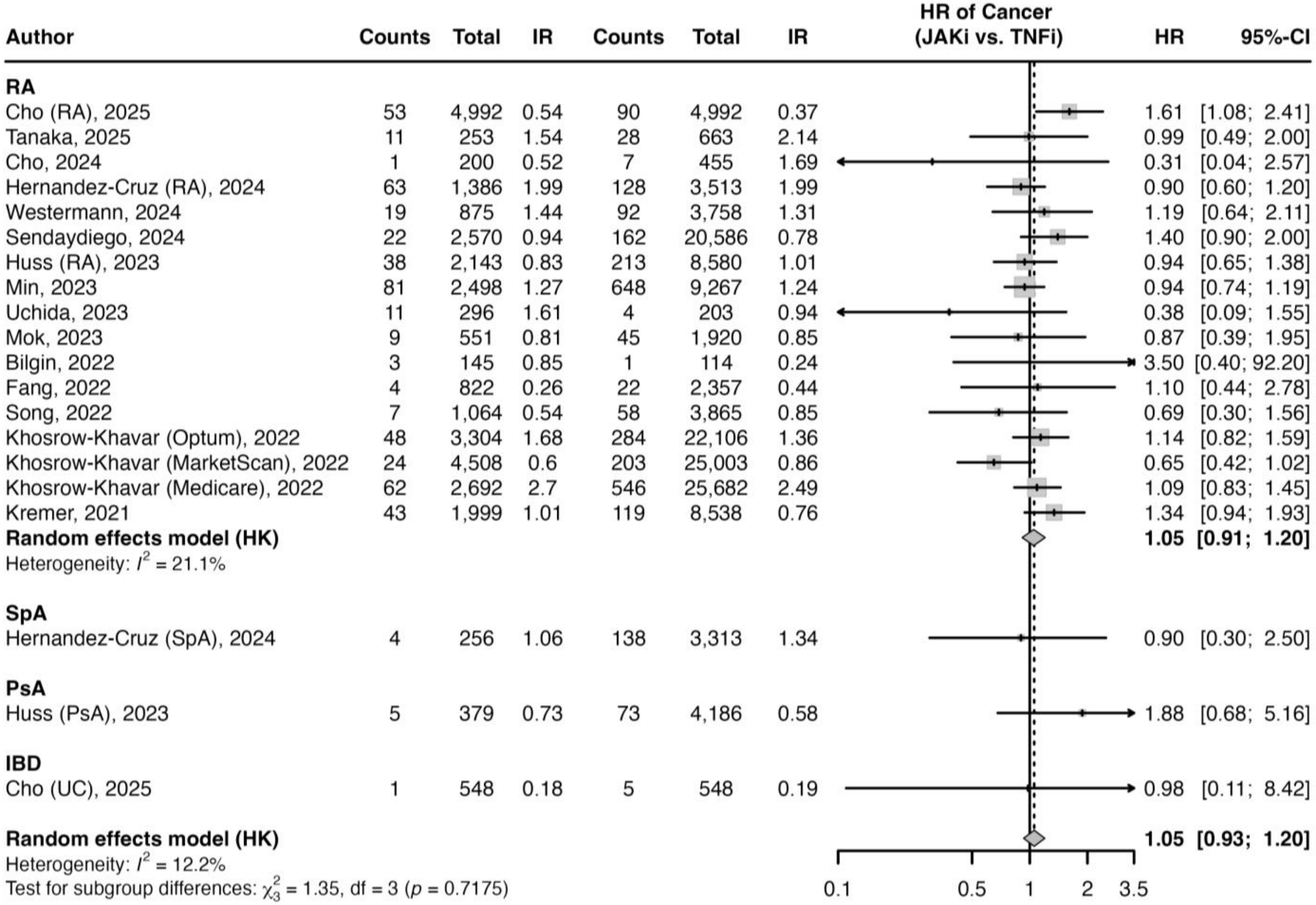

© 2025 Solitano V et al. *JAMA Network Open*.

eFigure 10. Risk of Malignant Neoplasms With JAK Inhibitors vs TNF Antagonists in All Patients With Immune-Mediated Inflammatory Diseases (Subgroup Analysis Based on Age Group)

Author	Counts	Total	IR	Counts	Total	IR	HR	95%-CI
**≥ 65 years**								
Westermann (≥ 65 years), 2024	11	268	2.74	65	1,364	2.39	1.34	[0.55; 2.81]
Song (≥ 65 years), 2022	-	-	-	-	-	-	0.60	[0.27; 1.31]
Khosrow-Khavar (Optum), 2022	16	722	2.3	104	4,482	2.74	0.97	[0.55; 1.71]
Khosrow-Khavar (MarketScan), 2022	12	489	2.72	34	2,126	1.83	1.12	[0.53; 2.34]
Khosrow-Khavar (Medicare), 2022	59	2,534	2.71	531	24,876	2.5	1.10	[0.83; 1.47]
**Random effects model (HK)**							**1.05**	**[0.82; 1.34]**
Heterogeneity: $I^2 = 0\%$								
**< 65 years**								
Song (< 65 years), 2022	-	-	-	-	-	-	0.93	[0.56; 1.53]
Khosrow-Khavar (Optum), 2022	32	2,582	1.49	180	17,624	1.05	1.22	[0.80; 1.85]
Khosrow-Khavar (MarketScan), 2022	12	4,019	0.34	169	22,877	0.77	0.45	[0.24; 0.82]
Khosrow-Khavar (Medicare), 2022	3	158	2.4	15	806	2.27	0.97	[0.23; 4.08]
**Random effects model (HK)**							**0.85**	**[0.41; 1.74]**
Heterogeneity: $I^2 = 56.8\%$								
**Random effects model (HK)**							**0.96**	**[0.75; 1.23]**
Heterogeneity: $I^2 = 20.4\%$								
Test for subgroup differences: $\chi^2_1 = 0.76$, df = 1 (p = 0.3848)								

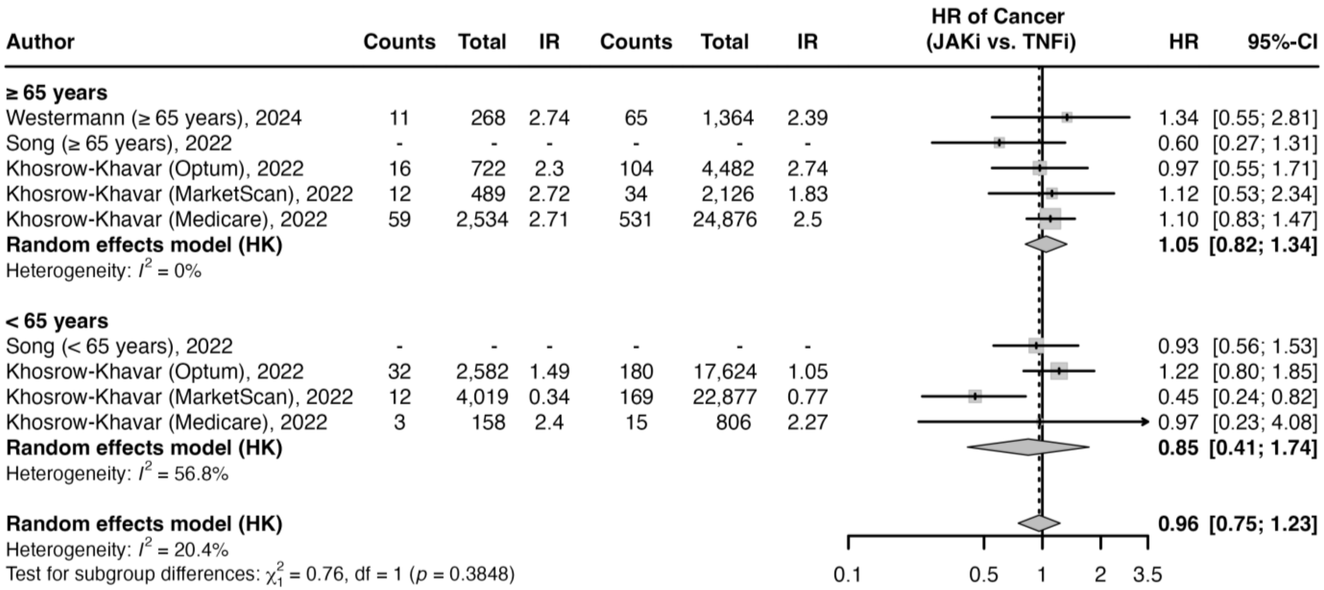

© 2025 Solitano V et al. *JAMA Network Open*.

eFigure 11. Funnel Plots for Publication Bias for Malignant Neoplasm Outcome

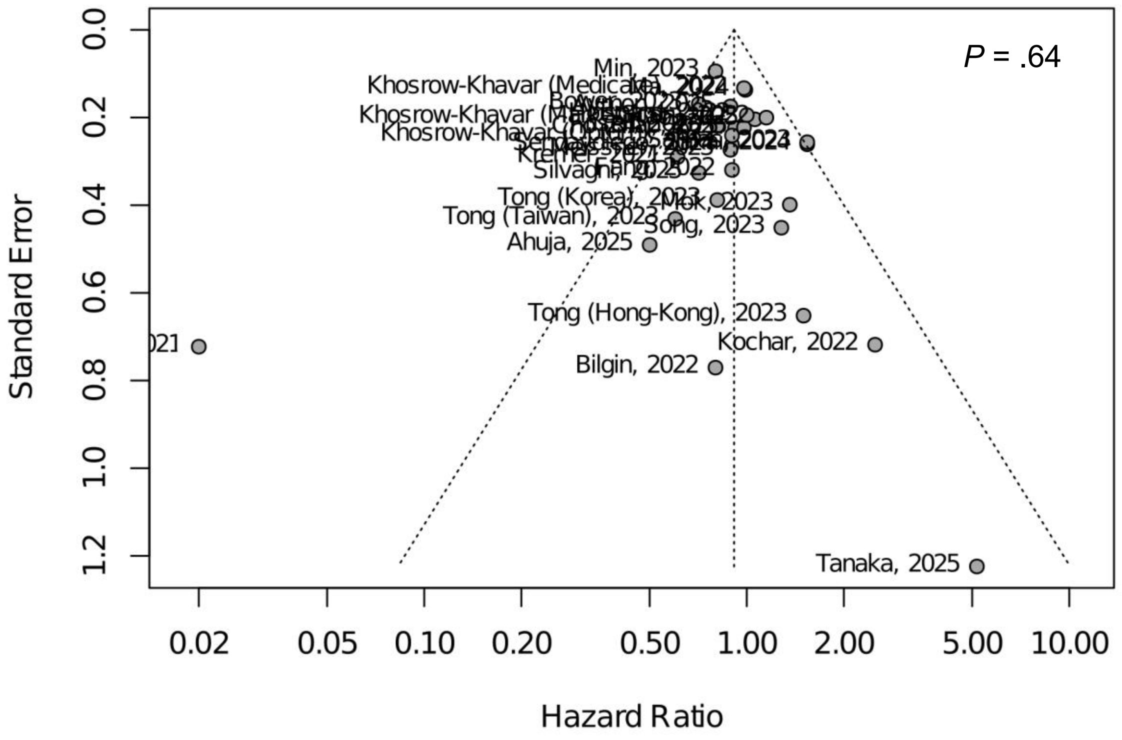

© 2025 Solitano V et al. *JAMA Network Open*.

eFigure 12. Risk of Major Cardiovascular Events With JAK Inhibitors vs TNF Antagonists in All Patients With Immune-Mediated Inflammatory Diseases (Subgroup Analysis Based on Location of Study)

Source	Counts	Total	IR	Counts	Total	IR	HR	95% CI
**`Site of Study` = North America**								
Ahuja, 2025	< 11	856	0.44	109	9,422	0.69	0.50	[0.19; 1.30]
Ma, 2024	104	1,995	--	108	1,995	--	0.99	[0.76; 1.30]
Sendaydiego, 2024	19	3,790	0.7	175	27,085	0.76	0.90	[0.50; 1.40]
Salinas (USA), 2023	7	1,913	0.97	9	1,913	0.68	1.25	[0.50; 3.12]
Khosrow-Khavar (Optum), 2022	24	3,763	0.73	143	24,805	0.61	0.90	[0.56; 1.44]
Khosrow-Khavar (MarketScan), 2022	35	5,307	0.75	141	28,776	0.52	1.15	[0.78; 1.71]
Khosrow-Khavar (Medicare), 2022	69	3,782	2.14	562	35,830	1.86	0.98	[0.76; 1.28]
Kochar, 2022	6	305	1.92	191	19,096	1	2.50	[0.37; 6.18]
Kremer, 2021	19	1,866	0.64	108	7,724	0.91	0.61	[0.34; 1.06]
Ozen, 2021	1	301	0.06	395	7,724	0.17	0.02	[0.01; 0.17]
Pooled summary							0.73	[0.33; 1.62]
Heterogeneity: $\chi^2_9 = 35.74$ ($P < .001$), $I^2 = 74.8\%$								
**`Site of Study` = Europe**								
Silvagni, 2025	18	1,443	0.57	24	2,343	0.62	0.71	[0.37; 1.33]
Frisell, 2023	37	2,263	0.98	240	8,748	1.2	0.81	[0.53; 1.25]
Bower, 2023	59	3,037	0.98	232	11,307	0.91	0.71	[0.51; 0.99]
Meissner, 2023	34	3,058	0.68	45	3,694	0.62	0.89	[0.52; 1.52]
Salinas (Europe), 2023	46	5,693	0.8	31	5,693	0.6	1.58	[0.99; 2.52]
Hoisnard, 2022	54	8,481	0.43	35	7,354	0.36	1.00	[0.70; 1.50]
Bilgin, 2022	3	145	0.84	4	114	1.01	0.80	[0.20; 4.10]
Pooled summary							0.91	[0.69; 1.19]
Heterogeneity: $\chi^2_6 = 8.62$ ($P = .20$), $I^2 = 30.4\%$								
**`Site of Study` = Asia**								
Cho (RA), 2025	40	4,992	0.41	84	4,992	0.34	0.92	[0.59; 1.42]
Tanaka, 2025	2	253	0.48	1	663	0.09	5.17	[0.47; 57.02]
Kao, 2025	29	1,462	8902	NA	213	--	1.06	[0.71; 1.58]
Sakai, 2024	20	4,416	0.11	114	12,327	0.06	1.54	[0.92; 2.56]
Min, 2023	131	2,498	2.09	1,248	9,267	2.49	0.80	[0.67; 0.97]
Mok, 2023	15	551	1.34	40	1,920	0.75	1.36	[0.62; 2.96]
Song, 2023	6	846	0.83	38	3,384	0.74	1.28	[0.53; 3.11]
Tong (Hong-Kong), 2023	4	294	1.09	7	598	0.42	1.50	[0.42; 5.41]
Tong (Korea), 2023	8	732	0.89	55	2,476	0.92	0.81	[0.38; 1.74]
Tong (Taiwan), 2023	6	545	0.76	61	2,775	0.88	0.60	[0.26; 1.41]
Fang, 2022	7	822	0.41	23	2,357	0.46	0.90	[0.48; 1.68]
Pooled summary							0.97	[0.80; 1.19]
Heterogeneity: $\chi^2_{10} = 11.7$ ($P = .31$), $I^2 = 14.5\%$								
Pooled summary							0.92	[0.80; 1.07]

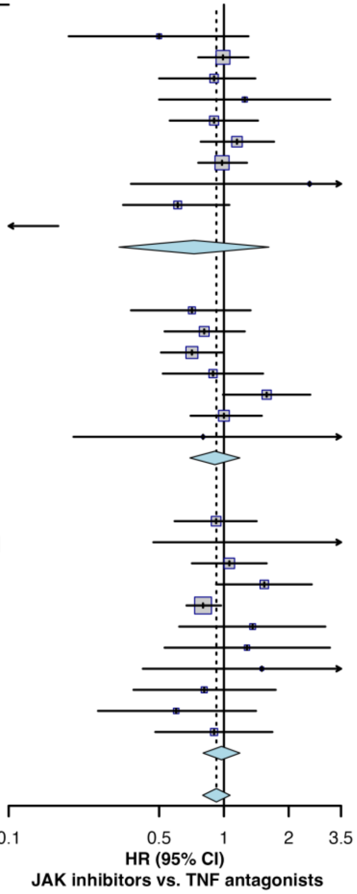

Heterogeneity: $\chi^2_{27} = 56.12$ ($P < .001$), $I^2 = 51.9\%$
Test for subgroup differences: $\chi^2_2 = 0.80$ ($P = .67$)

© 2025 Solitano V et al. *JAMA Network Open*.

eFigure 13. Risk of Major Cardiovascular Events (MACEs) With JAK Inhibitors vs TNF Antagonists in All Patients With Immune-Mediated Inflammatory Diseases (Subgroup Analysis Based on Subtype of MACE)

Author	Counts	Total	IR	Counts	Total	IR	HR	95%-CI
**Stroke**								
Sakai, 2024	7	4,416	1.12	28	12,327	1.21	1.56	[0.68; 3.57]
Frisell, 2023	13	1,837	0.45	98	8,748	0.4	0.91	[0.42; 1.96]
Min, 2023	35	2,498	0.54	391	9,267	0.73	0.74	[0.52; 1.06]
Kremer, 2023	10	1,999	0.32	60	8,358	0.47	0.67	[0.34; 1.31]
Bower, 2023	19	3,037	0.31	84	11,307	0.33	0.67	[0.37; 1.21]
Mok, 2023	6	551	0.54	19	1,920	0.36	0.93	[0.36; 2.41]
Khosrow-Khavar (Optum), 2022	9	3,763	0.27	58	24,805	0.25	0.72	[0.34; 1.55]
Khosrow-Khavar (MarketScan), 2022	13	5,307	0.28	37	28,776	0.14	1.33	[0.67; 2.65]
Khosrow-Khavar (Medicare), 2022	22	3,782	0.68	212	35,830	0.7	0.86	[0.54; 1.37]
Hoisnard, 2022	28	8,481	0.27	11	7,354	0.16	1.10	[0.60; 2.20]
Fang, 2022	5	822	0.33	23	2,357	0.46	0.75	[0.29; 1.94]
**Random effects model (HK)**							**0.85**	**[0.72; 1.00]**
Heterogeneity: $I^2 = 0\%$								
**Coronaries**								
Sakai, 2024	6	4,416	0.96	28	12,327	0.72	1.34	[0.55; 3.23]
Frisell, 2023	12	1,837	0.42	127	8,748	0.52	0.42	[0.21; 0.83]
Min, 2023	62	2,498	0.97	729	9,267	1.4	0.67	[0.51; 0.87]
Kremer, 2023	10	1,999	0.32	38	8,358	0.3	1.06	[0.53; 2.13]
Bower, 2023	24	3,037	0.4	113	11,307	0.44	0.58	[0.36; 0.94]
Mok, 2023	7	551	0.63	21	1,920	0.4	1.67	[0.65; 4.32]
Khosrow-Khavar (Optum), 2022	16	3,763	0.49	87	24,805	0.37	1.09	[0.62; 1.92]
Khosrow-Khavar (MarketScan), 2022	22	5,307	0.47	106	28,776	0.39	1.00	[0.61; 1.62]
Khosrow-Khavar (Medicare), 2022	50	3,782	1.54	368	35,830	1.21	1.05	[0.77; 1.44]
Hoisnard, 2022	29	8,481	0.28	24	7,354	0.35	0.90	[0.50; 1.50]
Fang, 2022	8	822	0.48	22	2,357	0.45	1.03	[0.45; 2.36]
**Random effects model (HK)**							**0.86**	**[0.69; 1.09]**
Heterogeneity: $I^2 = 37.6\%$								
**Random effects model (HK)**							**0.85**	**[0.75; 0.97]**
Heterogeneity: $I^2 = 5.9\%$								

Test for subgroup differences: $\chi^2_1 = 0.02$, df = 1 ($p = 0.8940$)

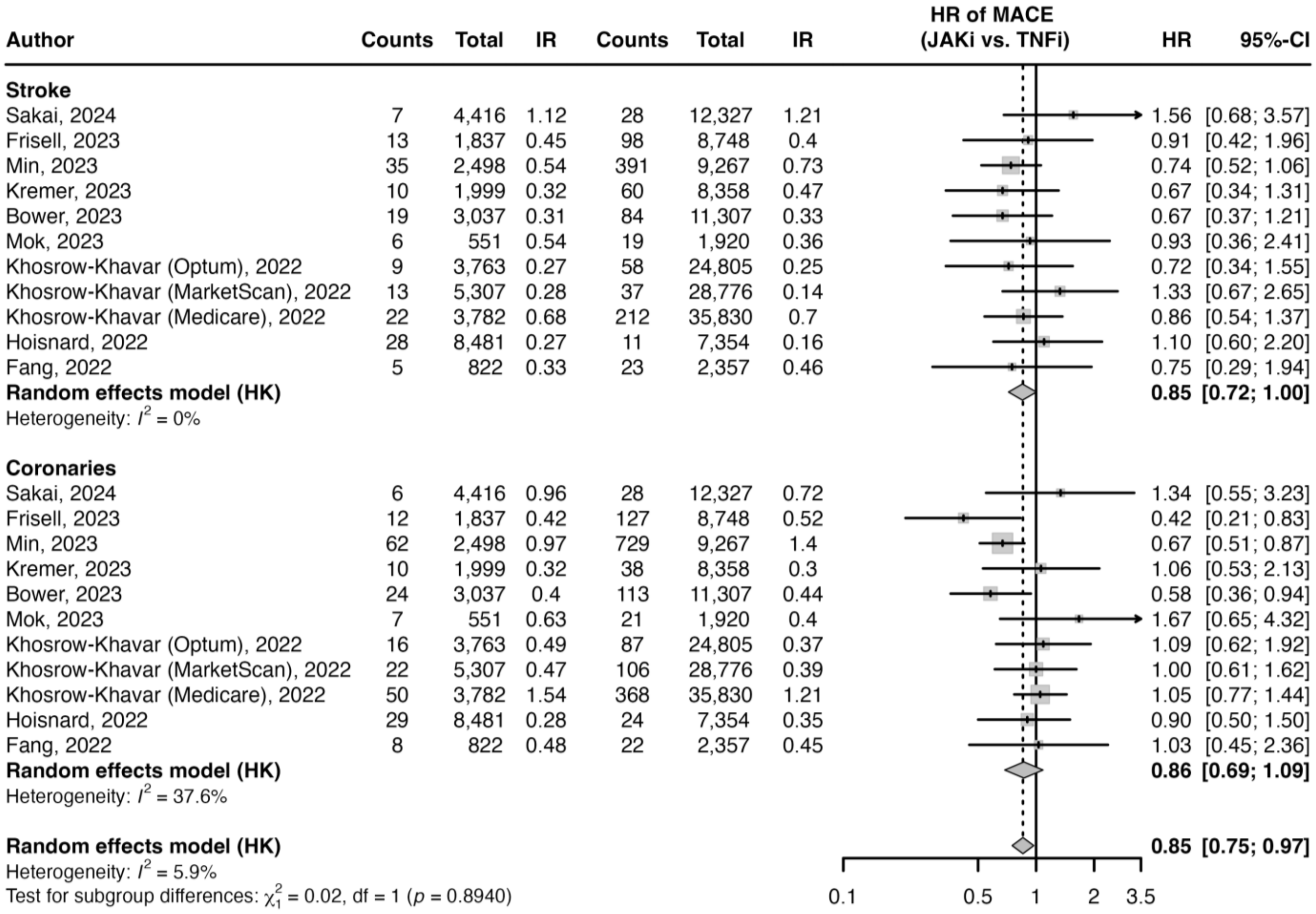

© 2025 Solitano V et al. *JAMA Network Open*.

eFigure 14. Risk of Major Cardiovascular Events With JAK Inhibitors vs TNF Antagonists in All Patients With Immune-Mediated Inflammatory Diseases (Subgroup Analysis Based on Type of JAK Inhibitor)

Source	Counts	Total	IR	Counts	Total	IR	HR	95% CI
**`Type of JAKi` -- Tofacitinib**								
Ahuja, 2025	< 11	659	0.44	109	9,422	0.69	0.44	[0.15; 1.28]
Cho (RA), 2025	19	2,019	0.38	84	4,992	0.34	0.82	[0.47; 1.41]
Tanaka, 2025	2	253	0.48	1	663	0.09	5.17	[0.47; 57.02]
Ma, 2024	104	1,995	--	108	1,995	--	0.99	[0.76; 1.30]
Frisell, 2023	7	426	0.92	240	8,748	1.2	0.78	[0.31; 1.33]
Bower, 2023	9	470	1.06	232	11,307	0.91	0.78	[0.39; 1.55]
Meissner, 2023	18	1,126	0.98	45	3,694	0.62	1.36	[0.70; 2.63]
Hoisnard, 2022	14	3,416	0.28	35	7,354	0.36	0.80	[0.40; 1.40]
Khosrow-Khavar (Optum), 2022	24	3,763	0.73	143	24,805	0.61	0.90	[0.56; 1.44]
Khosrow-Khavar (MarketScan), 2022	35	5,307	0.75	141	28,776	0.52	1.15	[0.78; 1.71]
Khosrow-Khavar (Medicare), 2022	69	3,782	2.14	562	35,830	1.86	0.98	[0.76; 1.28]
Bilgin, 2022	3	145	0.84	4	114	1.01	0.80	[0.20; 4.10]
Kochar, 2022	6	305	1.92	191	19,096	1	2.50	[0.37; 6.18]
Fang, 2022	7	822	0.41	23	2,357	0.46	0.90	[0.48; 1.68]
Kremer, 2021	19	1,866	0.64	108	7,724	0.91	0.61	[0.34; 1.06]
Ozen, 2021	1	301	0.06	395	7,724	0.17	0.02	[0.01; 0.17]
Pooled summary							0.84	[0.58; 1.22]
Heterogeneity: $\chi^2_{15} = 39.72$ ($P < .001$), $I^2 = 62.2\%$								
**`Type of JAKi` -- Baricitinib**								
Cho (RA), 2025	19	1,858	0.51	84	4,992	0.34	0.99	[0.53; 1.87]
Frisell, 2023	30	1,837	0.99	240	8,748	1.2	0.83	[0.49; 1.42]
Bower, 2023	45	1,973	1.01	232	11,307	0.91	0.70	[0.49; 1.01]
Meissner, 2023	12	1,416	0.49	45	3,694	0.62	0.58	[0.26; 1.26]
Salinas, 2023	54	7,606	0.92	39	7,606	0.6	1.54	[0.93; 2.54]
Hoisnard, 2022	40	5,065	0.52	35	7,354	0.36	1.10	[0.70; 1.80]
Pooled summary							0.93	[0.65; 1.32]
Heterogeneity: $\chi^2_{5} = 8.29$ ($P = .14$), $I^2 = 39.7\%$								
**`Type of JAKi` -- Upadacitinib**								
Ahuja, 2025	< 11	197	0.44	109	9,422	0.69	0.47	[0.07; 3.42]
Cho (RA), 2025	2	1,115	0.18	84	4,992	0.34	0.49	[0.10; 2.40]
Pooled summary							0.48	[0.37; 0.62]
Heterogeneity: $\chi^2_{1} = 0$ ($P = .97$), $I^2 = 0\%$								
Pooled summary							0.86	[0.68; 1.09]

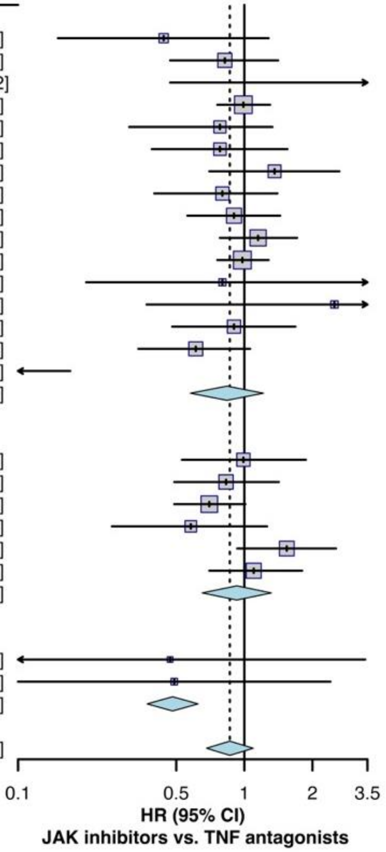

Heterogeneity: $\chi^2_{23} = 49.04$ ($P = .001$), $I^2 = 53.1\%$
Test for subgroup differences: $\chi^2_{2} = 31.69$ ($P < .001$)

© 2025 Solitano V et al. *JAMA Network Open*.

eFigure 15. Risk of Major Cardiovascular Events With JAK Inhibitors vs TNF Antagonists in All Patients With Immune-Mediated Inflammatory Diseases (Subgroup Analysis Based on Type of IMID)

Author	Counts	Total	IR	Counts	Total	IR	HR of MACE (JAKi vs. TNFi)	HR	95%-CI
**RA**									
Cho (RA), 2025	40	4,992	0.41	84	4,992	0.34		0.92	[0.59; 1.42]
Silvagni, 2025	18	1,443	0.57	24	2,343	0.62		0.71	[0.37; 1.33]
Tanaka, 2025	2	253	0.48	1	663	0.09		5.17	[0.47; 57.02]
Kao, 2025	29	1,462	0.97	213	8,902	0.84		1.06	[0.71; 1.58]
Ma, 2024	104	1,995	--	108	1,995	--		0.99	[0.76; 1.30]
Sakai, 2024	20	4,416	0.11	114	12,327	0.06		1.54	[0.92; 2.56]
Sendaydiego, 2024	19	3,790	0.7	175	27,085	0.76		0.90	[0.50; 1.40]
Frisell, 2023	37	2,263	0.98	240	8,748	1.2		0.81	[0.53; 1.25]
Min, 2023	131	2,498	2.09	1,248	9,267	2.49		0.80	[0.67; 0.97]
Bower, 2023	59	3,037	0.98	232	11,307	0.91		0.71	[0.51; 0.99]
Mok, 2023	15	551	1.34	40	1,920	0.75		1.36	[0.62; 2.96]
Meissner, 2023	34	3,058	0.68	45	3,694	0.62		0.89	[0.52; 1.52]
Song, 2023	6	846	0.83	38	3,384	0.74		1.28	[0.53; 3.11]
Tong (Hong-Kong), 2023	4	294	1.09	7	598	0.42		1.50	[0.42; 5.41]
Tong (Korea), 2023	8	732	0.89	55	2,476	0.92		0.81	[0.38; 1.74]
Tong (Taiwan), 2023	6	545	0.76	61	2,775	0.88		0.60	[0.26; 1.41]
Salinas, 2023	54	7,606	0.92	39	7,606	0.6		1.54	[0.93; 2.54]
Khosrow-Khavar (Optum), 2022	24	3,763	0.73	143	24,805	0.61		0.90	[0.56; 1.44]
Khosrow-Khavar (MarketScan), 2022	35	5,307	0.75	141	28,776	0.52		1.15	[0.78; 1.71]
Khosrow-Khavar (Medicare), 2022	69	3,782	2.14	562	35,830	1.86		0.98	[0.76; 1.28]
Hoisnard, 2022	54	8,481	0.43	35	7,354	0.36		1.00	[0.70; 1.50]
Bilgin, 2022	3	145	0.84	4	114	1.01		0.80	[0.20; 4.10]
Fang, 2022	7	822	0.41	23	2,357	0.46		0.90	[0.48; 1.68]
Kremer, 2021	19	1,866	0.64	108	7,724	0.91		0.61	[0.34; 1.06]
Ozen, 2021	1	301	0.06	395	7,724	0.17		0.02	[0.01; 0.17]
**Random effects model (HK)**								**0.92**	**[0.79; 1.06]**
Heterogeneity: I^2 = 53%									
**IBD**									
Ahuja, 2025	< 11	856	0.44	109	9,422	0.69		0.50	[0.19; 1.30]
Kochar, 2022	6	305	1.92	191	19,096	1		2.50	[0.37; 6.18]
**Random effects model (HK)**								**1.03**	**[0.00; 26718.38]**
Heterogeneity: I^2 = 70.8%									
**Random effects model (HK)**								**0.92**	**[0.79; 1.06]**
Heterogeneity: I^2 = 52.3%									
Test for subgroup differences: χ^2_1 = 0.02, df = 1 (p = 0.8875)									

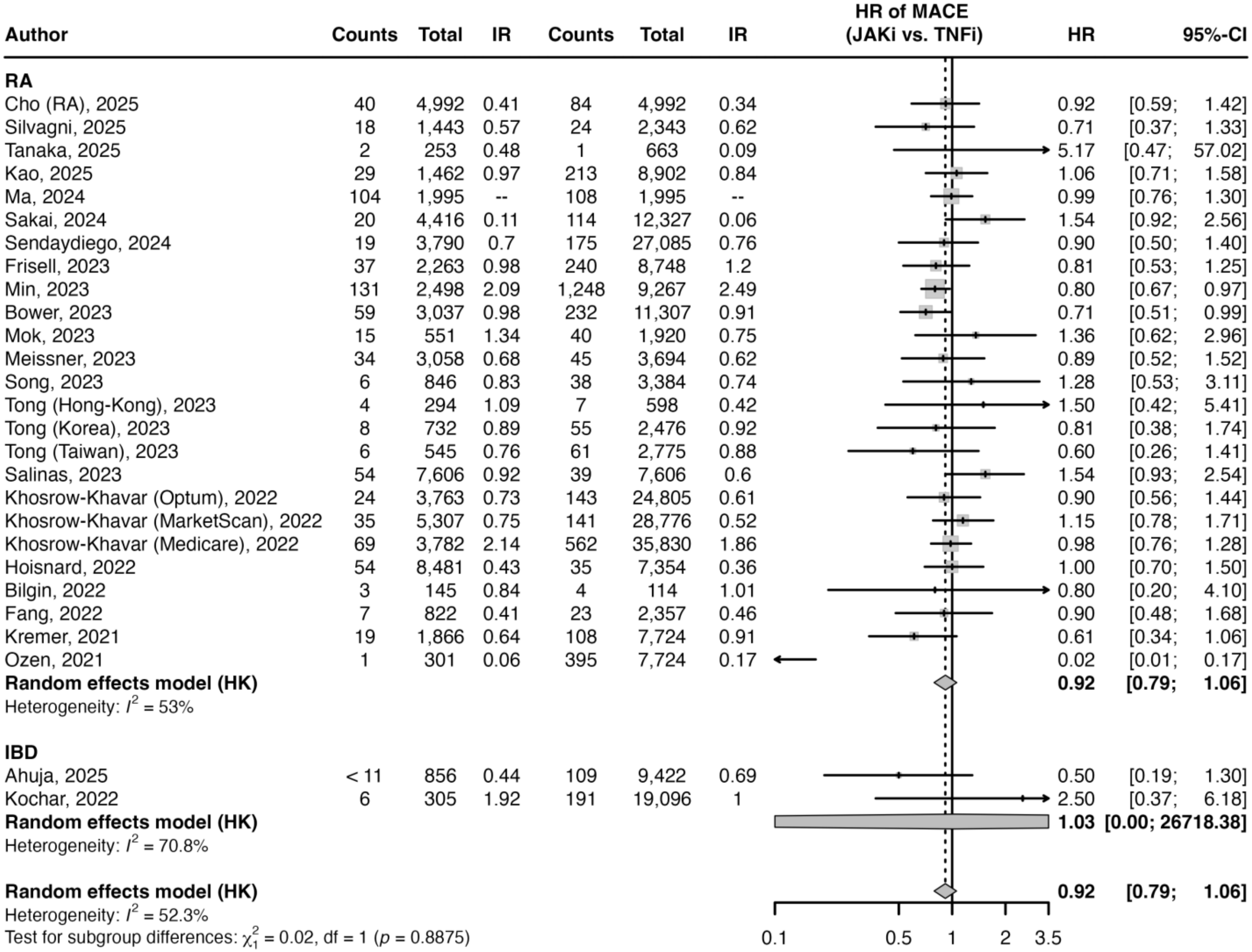

© 2025 Solitano V et al. *JAMA Network Open*.

eFigure 16. Risk of Major Cardiovascular Events With JAK inhibitors vs TNF Antagonists in All Patients With Immune-Mediated Inflammatory Diseases (Subgroup Analysis Based on Age Group)

Author	Counts	Total	IR	Counts	Total	IR	HR	95%-CI
**< 65 years**								
Sendaydiego, 2024	19	3,790	0.7	175	27,085	0.76	0.90	[0.50; 1.40]
Ma, 2024	--	--	--	--	--	--	0.87	[0.63; 1.21]
Meissner, 2023	15	2,035	0.47	21	2,572	0.43	0.71	[0.32; 1.55]
Song, 2023	4	765	0.56	29	3,093	0.57	1.13	[0.39; 3.30]
Khosrow-Khavar (Optum), 2022	11	2,836	0.46	72	19,203	0.39	0.98	[0.49; 1.97]
Khosrow-Khavar (MarketScan), 2022	19	4,613	0.47	100	25,587	0.41	1.10	[0.65; 1.85]
Khosrow-Khavar (Medicare), 2022	1	179	0.72	12	935	1.53	0.24	[0.03; 2.05]
**Random effects model (HK)**							**0.90**	**[0.76; 1.08]**
Heterogeneity: $I^2 = 0\%$								
**≥ 65 years**								
Ma, 2024	--	--	--	--	--	--	1.13	[0.76; 1.67]
Min, 2023	--	--	--	--	--	--	0.85	[0.64; 1.12]
Meissner, 2023	19	1,023	1.06	24	1,122	1.03	1.07	[0.51; 2.21]
Song, 2023	2	81	0.28	9	291	0.18	1.83	[0.36; 9.31]
Khosrow-Khavar (Optum), 2022	13	927	1.49	71	5,602	1.48	0.87	[0.46; 1.65]
Khosrow-Khavar (MarketScan), 2022	16	694	2.61	41	3,189	1.48	1.66	[0.88; 3.15]
Khosrow-Khavar (Medicare), 2022	68	3,603	2.2	550	34,895	1.86	1.01	[0.77; 1.32]
Hoisnard, 2022	26	1,917	1.13	14	1,183	1.26	0.70	[0.40; 1.30]
**Random effects model (HK)**							**0.98**	**[0.82; 1.16]**
Heterogeneity: $I^2 = 0\%$								
**Random effects model (HK)**							**0.95**	**[0.85; 1.06]**
Heterogeneity: $I^2 = 0.0\%$								
Test for subgroup differences: $\chi^2_1 = 0.58$, df = 1 ($p = 0.4472$)								

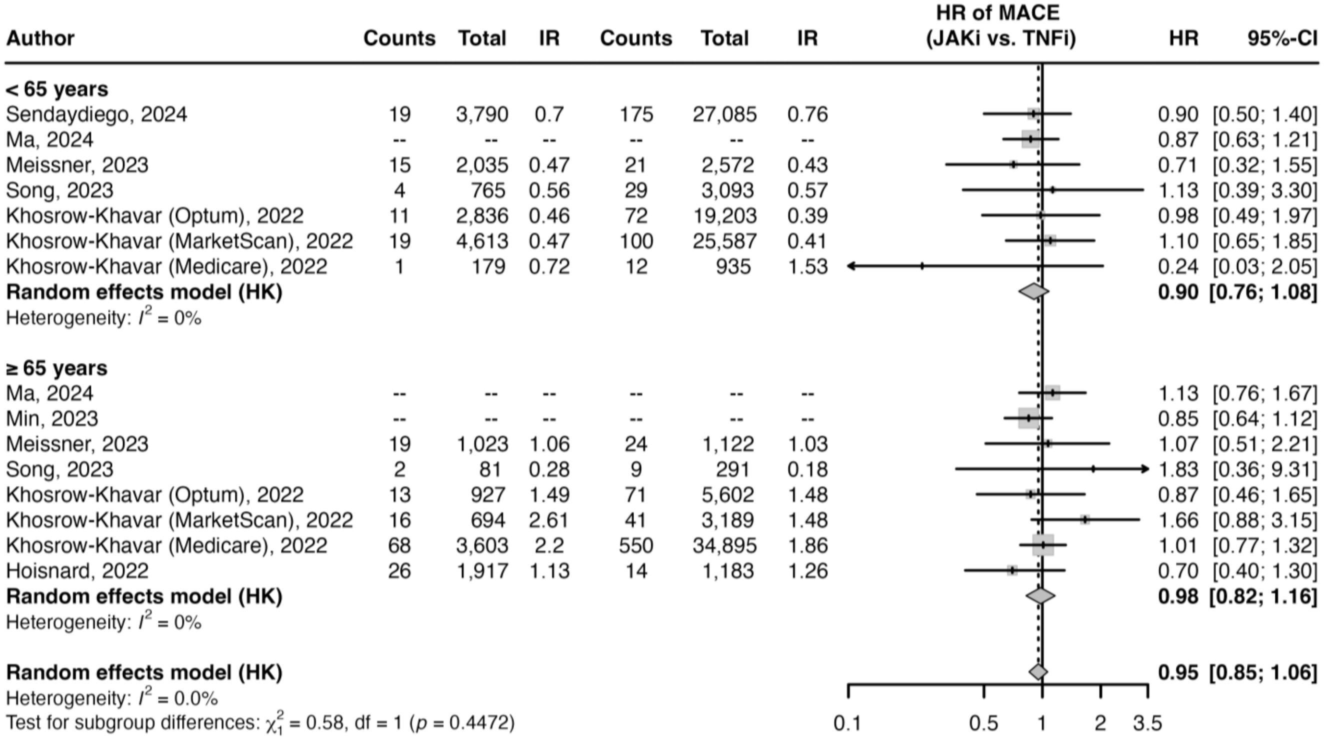

© 2025 Solitano V et al. *JAMA Network Open*.

eFigure 17. Funnel Plots for Publication Bias for Major Cardiovascular Event Outcome

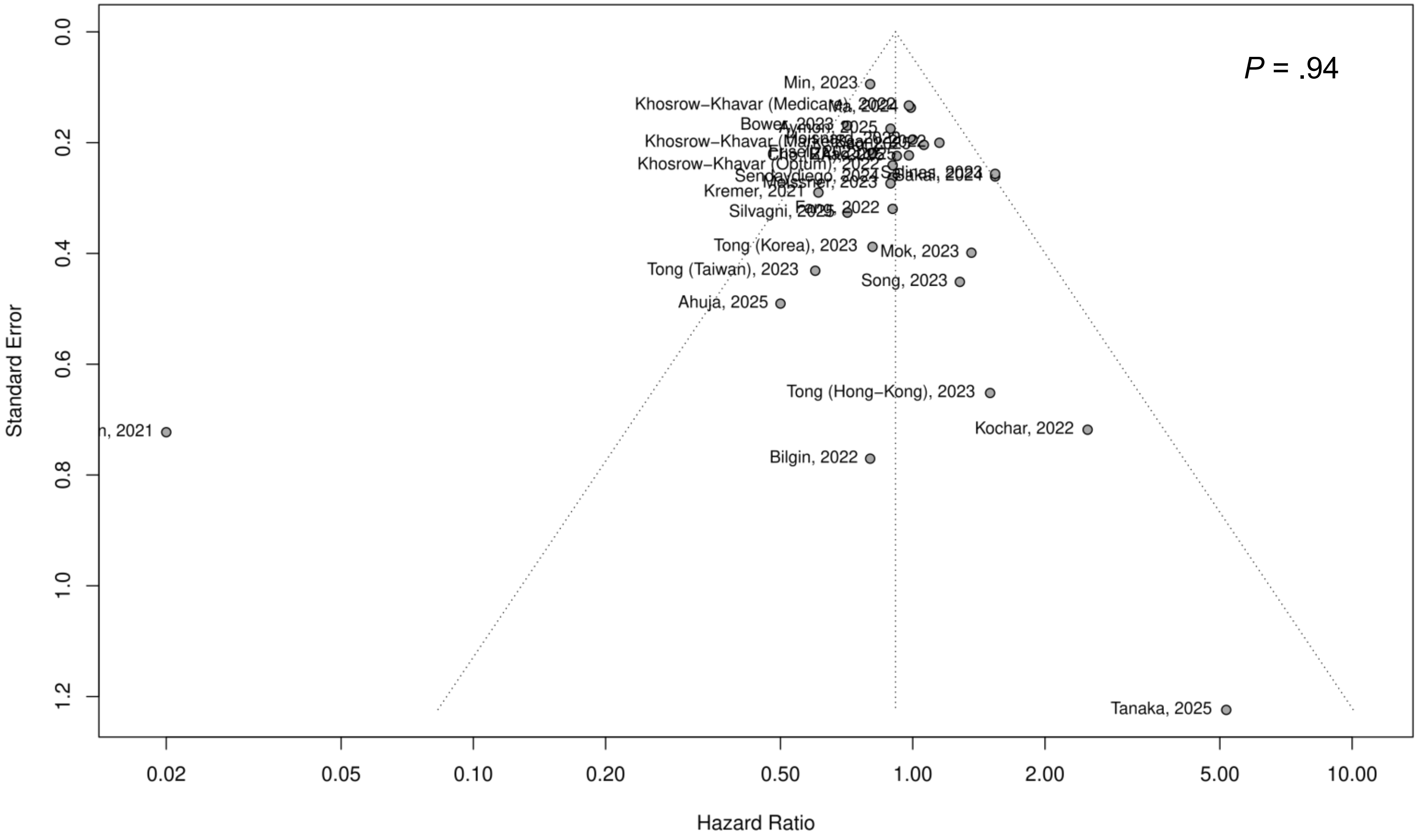

© 2025 Solitano V et al. *JAMA Network Open*.

eFigure 18. Risk of Venous Thromboembolism With JAK Inhibitors vs TNF Antagonists in All Patients With Immune-Mediated Inflammatory Diseases (Subgroup Analysis Based on Location of Study)

Source	Counts	Total	IR	Counts	Total	IR	HR	95% CI
**`Site of Study` -- North America**								
Ahuja, 2025	15	856	1.32	191	9,422	1.22	0.66	[0.28; 1.57]
Salinas (US), 2023	6	748	2.5	4	748	1.06	2.40	[0.68; 8.52]
Kochar, 2022	3	305	1.58	191	19,096	0.99	1.49	[0.01; 4.48]
Desai (Marketscan), 2021	10	2,992	0.42	134	39,209	0.35	1.05	[0.55; 2.00]
Desai (Medicare), 2021	16	1,795	1.18	174	23,283	0.83	1.41	[0.84; 2.36]
Desai (Optum), 2021	3	1,987	0.19	57	18,387	0.34	0.47	[0.15; 1.50]
Kremer, 2021	9	1,999	0.29	41	8,354	0.33	0.89	[0.44; 1.84]
Pooled summary							1.06	[0.72; 1.55]
Heterogeneity: $\chi_6^2 = 6.13$ ($P = .41$), $I^2 = 2.2\%$								
**`Site of Study` -- Europe**								
Silvagni, 2025	13	1,443	0.41	9	2,343	0.23	1.53	[0.65; 3.65]
Salinas (Europe), 2023	46	5,309	0.94	37	5,309	0.62	1.51	[0.96; 2.40]
Molander, 2023	48	2,354	1.1	287	19,950	0.52	1.73	[1.24; 2.42]
Hoisnard, 2022	75	8,481	0.6	32	7,354	0.33	1.10	[0.70; 1.60]
Pooled summary							1.46	[1.03; 2.05]
Heterogeneity: $\chi_3^2 = 2.82$ ($P = .42$), $I^2 = 0\%$								
**`Site of Study` -- Asia**								
Tanaka, 2025	1	253	0.55	1	663	--	2.59	[0.16; 41.42]
Kao, 2025	7	1,462	0.23	77	8,902	0.3	0.71	[0.32; 1.56]
Sakai, 2024	43	4,416	0.69	129	12,327	3.32	1.85	[1.31; 2.62]
Min, 2023	31	2,498	0.48	193	9,267	0.36	1.34	[0.90; 1.99]
Song, 2023	3	871	0.29	21	3,307	0.35	0.18	[0.01; 3.47]
Fang, 2022	5	822	0.32	24	2,357	0.48	0.65	[0.25; 1.70]
Pooled summary							1.16	[0.64; 2.09]
Heterogeneity: $\chi_5^2 = 9.89$ ($P = .08$), $I^2 = 49.5\%$								
Pooled summary							1.27	[1.04; 1.55]

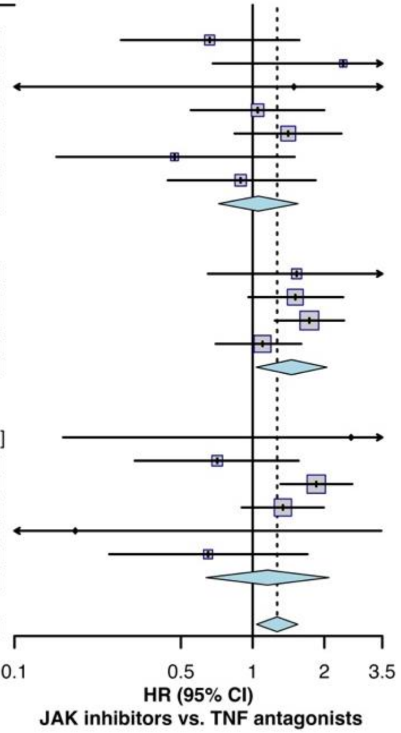

Heterogeneity: $\chi_{16}^2 = 21.92$ ($P = .15$), $I^2 = 27.0\%$

Test for subgroup differences: $\chi_2^2 = 3.12$ ($P = .21$)

© 2025 Solitano V et al. *JAMA Network Open*.

eFigure 19. Risk of Venous Thromboembolism With JAK Inhibitors vs TNF Antagonists in All Patients With Immune-Mediated Inflammatory Diseases (Subgroup Analysis Based on Subtype of VTE)

Source	Counts	Total	IR	Counts	Total	IR	HR	95% CI
**`Subtype of VTE` = Deep Vein Thrombosis (DVT)**								
Sakai, 2024	36	4,416	5.78	107	12,327	2.76	2.10	[1.44; 3.06]
Song, 2023	3	871	0.29	18	3,307	0.3	0.22	[0.01; 4.32]
Molander, 2023	--	--	--	--	--	--	0.83	[0.47; 1.45]
Desai (Marketscan), 2021	21	2,992	1.37	203	39,209	1.22	0.87	[0.55; 1.35]
Desai (Medicare), 2021	24	1,795	1	334	23,283	0.88	1.00	[0.66; 1.52]
Desai (Optum), 2021	32	1,987	2.39	437	18,387	2.11	1.10	[0.77; 1.58]
Kremer, 2021	4	1,866	1.3	20	7,767	0.16	0.82	[0.28; 2.40]
Pooled summary							1.09	[0.75; 1.59]
Heterogeneity: $\chi^2_6 = 14.58$ ($P = .02$), $I^2 = 58.9\%$								
**`Subtype of VTE` = Pulmonary Embolism (PE)**								
Sakai, 2024	7	4,416	1.12	27	12,327	0.7	1.62	[0.70; 3.71]
Sakai, 2024	7	4,416	1.12	27	12,327	0.7	1.62	[0.70; 3.71]
Molander, 2023	--	--	--	--	--	--	3.21	[2.11; 4.88]
Molander, 2023	--	--	--	--	--	--	3.21	[2.11; 4.88]
Desai (Marketscan), 2021	5	2,992	0.21	75	39,209	0.2	0.97	[0.39; 2.41]
Desai (Medicare), 2021	11	1,795	0.59	93	23,283	0.44	1.26	[0.61; 2.60]
Desai (Optum), 2021	2	1,987	1.13	35	18,387	0.21	0.51	[0.12; 2.10]
Kremer, 2021	6	1,866	0.19	24	7,767	0.19	1.02	[0.42; 2.50]
Desai (Marketscan), 2021	5	2,992	0.21	75	39,209	0.2	0.97	[0.39; 2.41]
Desai (Medicare), 2021	11	1,795	0.59	93	23,283	0.44	1.26	[0.61; 2.60]
Desai (Optum), 2021	2	1,987	1.13	35	18,387	0.21	0.51	[0.12; 2.10]
Kremer, 2021	6	1,866	0.19	24	7,767	0.19	1.02	[0.42; 2.50]
Pooled summary							1.44	[1.00; 2.07]
Heterogeneity: $\chi^2_{11} = 29.14$ ($P = .002$), $I^2 = 62.3\%$								
Pooled summary							1.28	[0.99; 1.65]

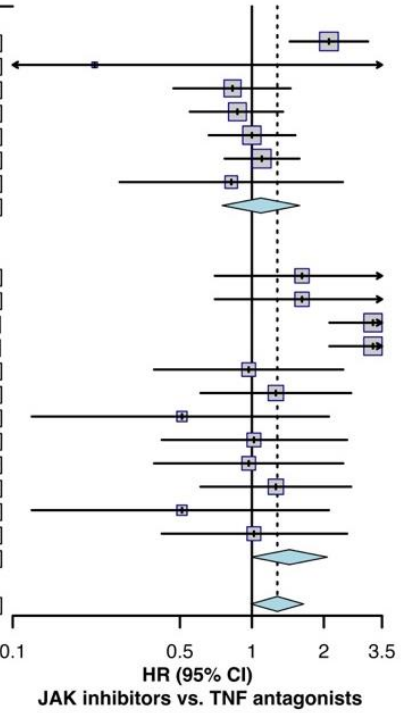

Heterogeneity: $\chi^2_{18} = 55.12$ ($P < .001$), $I^2 = 67.3\%$
Test for subgroup differences: $\chi^2_1 = 1.48$ ($P = .22$)

© 2025 Solitano V et al. *JAMA Network Open*.

eFigure 20. Risk of Venous Thromboembolism With JAK inhibitors vs TNF Antagonists in All Patients With Immune-Mediated Inflammatory Diseases (Subgroup Analysis Based on Type of JAK Inhibitors)

Source	Counts	Total	IR	Counts	Total	IR	HR	95% CI
**`Type of JAKi` -- Tofacitinib**								
Ahuja, 2025	12	659	1.32	191	9,422	1.22	0.73	[0.25; 2.07]
Tanaka, 2025	1	253	0.55	1	663	--	2.59	[0.16; 41.42]
Molander, 2023	7	424	1.1	287	19,950	0.52	1.66	[0.77; 3.59]
Kochar, 2022	3	305	1.58	191	19,096	0.99	1.49	[0.01; 4.48]
Fang, 2022	5	822	0.32	24	2,357	0.48	0.65	[0.25; 1.70]
Hoisnard, 2022	29	3,416	0.59	32	7,354	3.3	1.10	[0.60; 1.80]
Desai (Marketscan), 2021	10	2,992	0.42	134	39,209	0.35	1.05	[0.55; 2.00]
Desai (Medicare), 2021	16	1,795	1.18	174	23,283	0.83	1.41	[0.84; 2.36]
Desai (Optum), 2021	3	1,987	0.19	57	18,387	0.34	0.47	[0.15; 1.50]
Kremer, 2021	9	1,999	0.29	41	8,354	0.33	0.89	[0.44; 1.84]
Pooled summary							1.08	[0.84; 1.38]
Heterogeneity: $\chi^2_9 = 6.55$ (P = .68), $I^2 = 0\%$								
**`Type of JAKi` -- Baricitinib**								
Salinas, 2023	56	7,606	0.95	41	7,606	0.63	1.51	[1.10; 2.08]
Molander, 2023	41	1,825	1.1	287	19,950	0.52	1.79	[1.25; 2.55]
Hoisnard, 2022	46	5,065	0.6	32	7,354	3.3	1.20	[0.70; 1.80]
Pooled summary							1.53	[0.99; 2.37]
Heterogeneity: $\chi^2_2 = 1.77$ (P = .41), $I^2 = 0\%$								
**`Type of JAKi` -- Upadacitinib**								
Ahuja, 2025	NA	197	1.32	191	9,422	1.22	0.64	[0.20; 2.08]
Pooled summary							1.25	[1.03; 1.52]

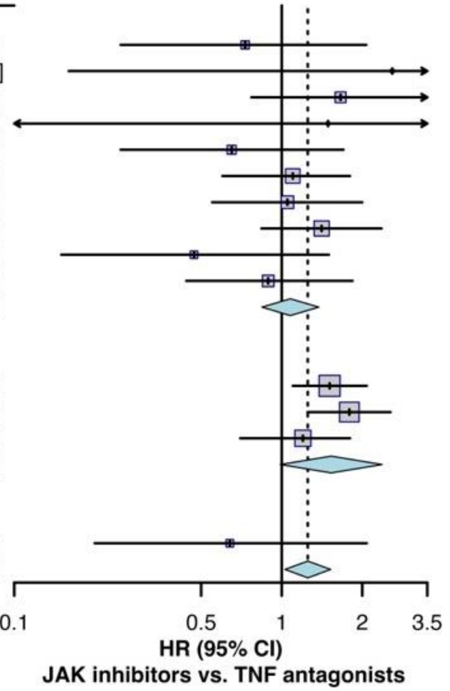

Heterogeneity: $\chi^2_{13} = 14.19$ (P = .36), $I^2 = 8.4\%$
Test for subgroup differences: $\chi^2_2 = 6.97$ (P = .03)

© 2025 Solitano V et al. *JAMA Network Open*.

eFigure 21. Risk of Venous Thromboembolism With JAK inhibitors vs TNF Antagonists in All Patients With Immune-Mediated Inflammatory Diseases (IMIDs) (Subgroup Analysis Based on Type of IMID)

Source	Counts	Total	IR	Counts	Total	IR	HR	95% CI
`Type of disease` = RA								
Silvagni, 2025	13	1,443	0.41	9	2,343	0.23	1.53	[0.65; 3.65]
Tanaka, 2025	1	253	0.55	1	663	--	2.59	[0.16; 41.42]
Kao, 2025	7	1,462	0.23	77	8,902	0.3	0.71	[0.32; 1.56]
Sakai, 2024	43	4,416	0.69	129	12,327	3.32	1.85	[1.31; 2.62]
Min, 2023	31	2,498	0.48	193	9,267	0.36	1.34	[0.90; 1.99]
Song, 2023	3	871	0.29	21	3,307	0.35	0.18	[0.01; 3.47]
Molander, 2023	48	2,354	1.1	287	19,950	0.52	1.73	[1.24; 2.42]
Salinas, 2023	56	7,606	0.95	41	7,606	0.63	1.51	[1.10; 2.08]
Hoisnard, 2022	75	8,481	0.6	32	7,354	0.33	1.10	[0.70; 1.60]
Fang, 2022	5	822	0.32	24	2,357	0.48	0.65	[0.25; 1.70]
Desai (Marketscan), 2021	10	2,992	0.42	134	39,209	0.35	1.05	[0.55; 2.00]
Desai (Medicare), 2021	16	1,795	1.18	174	23,283	0.83	1.41	[0.84; 2.36]
Desai (Optum), 2021	3	1,987	0.19	57	18,387	0.34	0.47	[0.15; 1.50]
Kremer, 2021	9	1,999	0.29	41	8,354	0.33	0.89	[0.44; 1.84]
Pooled summary							1.31	[1.07; 1.60]
Heterogeneity: χ^2_{13} = 18.67 (*P* = .13), I^2 = 30.4%								
`Type of disease` = IBD								
Ahuja, 2025	15	856	1.32	191	9,422	1.22	0.66	[0.28; 1.57]
Kochar, 2022	3	305	1.58	191	19,096	0.99	1.49	[0.01; 4.48]
Pooled summary							0.70	[0.05; 10.49]
Heterogeneity: χ^2_{1} = 0.25 (*P* = .61), I^2 = 0%								
Pooled summary							1.26	[1.03; 1.54]

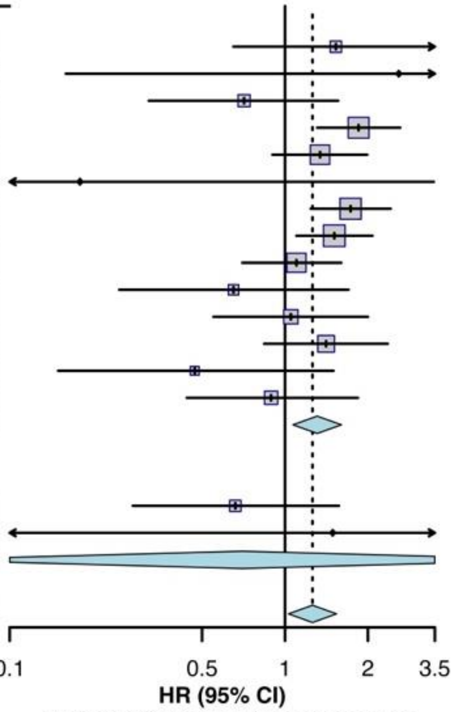

Heterogeneity: χ^2_{15} = 21.38 (*P* = .13), I^2 = 29.8%
Test for subgroup differences: χ^2_{1} = 7.20 (*P* = .007)

© 2025 Solitano V et al. *JAMA Network Open*.

eFigure 22. Risk of Venous Thromboembolism With JAK Inhibitors vs TNF Antagonists in All Patients With Immune-Mediated Inflammatory Diseases (Subgroup Analysis Based on Age Group)

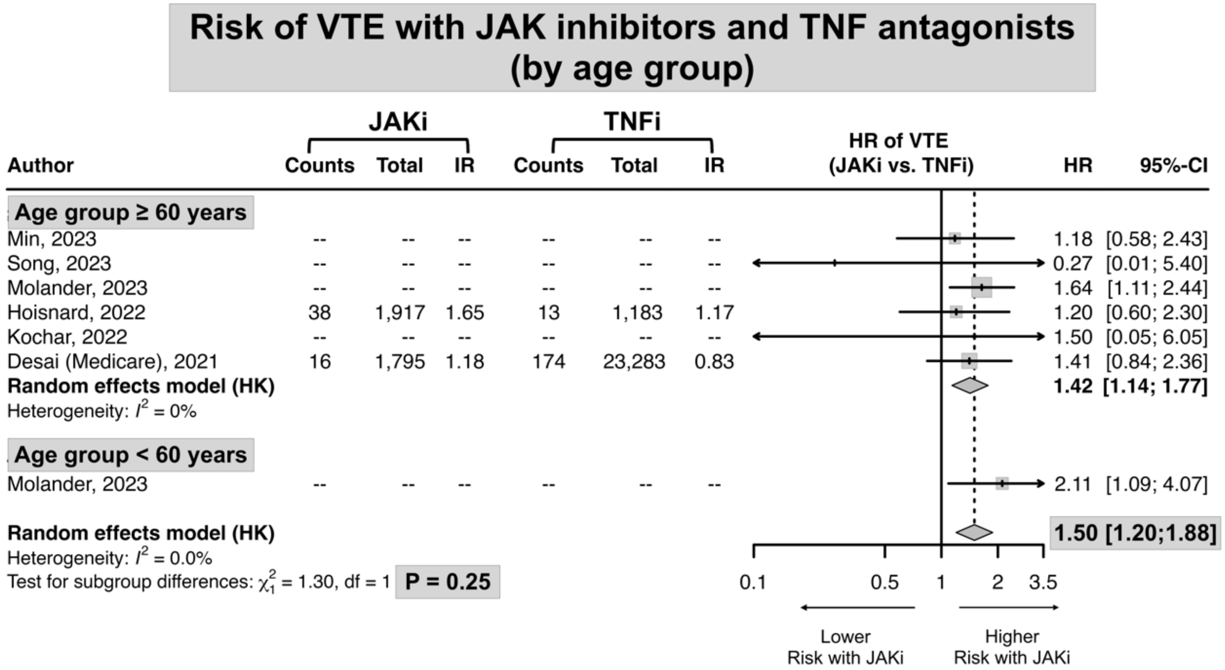

© 2025 Solitano V et al. *JAMA Network Open*.

eFigure 23. Funnel Plots for Publication Bias for Venous Thromboembolism Outcome

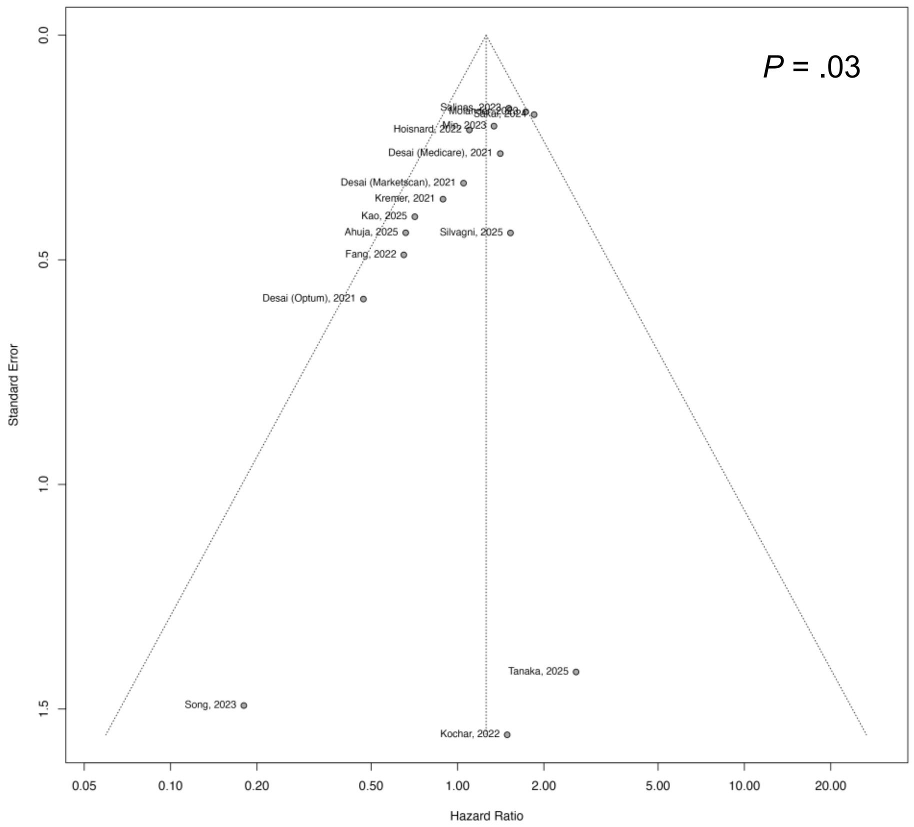

© 2025 Solitano V et al. *JAMA Network Open*.

eReferences

1. Wells GA, Wells G, Shea B, et al. The Newcastle-Ottawa Scale (NOS) for Assessing the Quality of Nonrandomised Studies in Meta-Analyses, 2014.
2. Hartung J, Knapp G. A refined method for the meta-analysis of controlled clinical trials with binary outcome. Stat Med 2001;20:3875-89.
3. Tanriver-Ayder E, Faes C, van de Casteele T, et al. Comparison of commonly used methods in random effects meta-analysis: application to preclinical data in drug discovery research. BMJ Open Sci 2021;5:e100074.
4. Partlett C, Riley RD. Random effects meta-analysis: Coverage performance of 95% confidence and prediction intervals following REML estimation. Stat Med 2017;36:301-317.
5. Higgins JP, Thompson SG. Quantifying heterogeneity in a meta-analysis. Stat Med 2002;21:1539-58.
6. Higgins JP, Thompson SG, Deeks JJ, et al. Measuring inconsistency in meta-analyses. BMJ 2003;327:557-60.
7. IntHout J, Ioannidis JP, Borm GF. The Hartung-Knapp-Sidik-Jonkman method for random effects meta-analysis is straightforward and considerably outperforms the standard DerSimonian-Laird method. BMC Med Res Methodol 2014;14:25.
8. Sterne JA, Egger M, Smith GD. Systematic reviews in health care: Investigating and dealing with publication and other biases in meta-analysis. BMJ 2001;323:101-5.
9. Ahuja D, Yeh KH, Patel SB, et al. Comparative Safety of Janus Kinase inhibitors vs. Tumor Necrosis Factor Antagonists in Patients with Inflammatory Bowel Diseases. Clin Gastroenterol Hepatol 2025.
10. Bastard L, Claudepierre P, Penso L, et al. Risk of serious infection associated with different classes of targeted therapies used in psoriatic arthritis: a nationwide cohort study from the French Health Insurance Database (SNDS). RMD Open 2024;10.
11. Bower H, Frisell T, di Giuseppe D, et al. Comparative cardiovascular safety with janus kinase inhibitors and biological disease-modifying antirheumatic drugs as used in clinical practice: an observational cohort study from Sweden in patients with rheumatoid arthritis. RMD Open 2023;9.
12. Huss V, Bower H, Wadstrom H, et al. Short- and longer-term cancer risks with biologic and targeted synthetic disease-modifying antirheumatic drugs as used against rheumatoid arthritis in clinical practice. Rheumatology (Oxford) 2022;61:1810-1818.
13. Huss V, Bower H, Hellgren K, et al. Cancer risks with JAKi and biological disease-modifying antirheumatic drugs in patients with rheumatoid arthritis or psoriatic arthritis: a national real-world cohort study. Ann Rheum Dis 2023;82:911-919.

© 2025 Solitano V et al. *JAMA Network Open*.

14. Khosrow-Khavar F, Desai RJ, Lee H, et al. Tofacitinib and Risk of Malignancy: Results From the Safety of Tofacitinib in Routine Care Patients With Rheumatoid Arthritis (STAR-RA) Study. Arthritis Rheumatol 2022;74:1648-1659.
15. Min HK, Kim H, Jeong HJ, et al. Risk of cancer, cardiovascular disease, thromboembolism, and mortality in patients with rheumatoid arthritis receiving Janus kinase inhibitors: a real-world retrospective observational study using Korean health insurance data. Epidemiol Health 2023;45:e2023045.
16. Uchida T, Iwamoto N, Fukui S, et al. Comparison of risks of cancer, infection, and MACEs associated with JAK inhibitor and TNF inhibitor treatment: a multicentre cohort study. Rheumatology (Oxford) 2023;62:3358-3365.
17. Kremer JM, Bingham CO, 3rd, Cappelli LC, et al. Postapproval Comparative Safety Study of Tofacitinib and Biological Disease-Modifying Antirheumatic Drugs: 5-Year Results from a United States-Based Rheumatoid Arthritis Registry. ACR Open Rheumatol 2021;3:173-184.
18. Bilgin E, Duran E, Unaldi E, et al. Comparison of cardiovascular, cancer and herpes zoster risk of tofacitinib versus etanercept: single-centre observational study. Rheumatology (Oxford) 2022;61:e267-e269.
19. Cheng D, Kochar BD, Cai T, et al. Risk of Infections With Ustekinumab and Tofacitinib Compared to Tumor Necrosis Factor alpha Antagonists in Inflammatory Bowel Diseases. Clin Gastroenterol Hepatol 2022;20:2366-2372 e6.
20. Cho SK, Song YJ, Kim HW, et al. Comparative effectiveness of tofacitinib and tumour necrosis factor inhibitors in patients with rheumatoid arthritis in real-world practice: a prospective observational study. Rheumatology (Oxford) 2025;64:541-547.
21. Choi SR, Shin A, Ha YJ, et al. Comparative risk of infections between JAK inhibitors versus TNF inhibitors among patients with rheumatoid arthritis: a cohort study. Arthritis Res Ther 2023;25:129.
22. Machado MAA, Moura CS, Guerra SF, et al. Effectiveness and safety of tofacitinib in rheumatoid arthritis: a cohort study. Arthritis Res Ther 2018;20:60.
23. Fang YF, Liu JR, Chang SH, et al. Comparative safety of Janus kinase inhibitors and tumor necrosis factor inhibitors in patients undergoing treatment for rheumatoid arthritis. Int J Rheum Dis 2022;25:1254-1262.
24. Frisell T, Bower H, Morin M, et al. Safety of biological and targeted synthetic disease-modifying antirheumatic drugs for rheumatoid arthritis as used in clinical practice: results from the ARTIS programme. Ann Rheum Dis 2023;82:601-610.
25. Hernandez-Cruz B, Otero-Varela L, Freire-Gonzalez M, et al. Janus kinase inhibitors and tumour necrosis factor inhibitors show a favourable safety profile and similar persistence in rheumatoid arthritis, psoriatic arthritis and spondyloarthritis: real-world data from the BIOBADASER registry. Ann Rheum Dis 2024;83:1189-1199.
26. Hoisnard L, Pina Vegas L, Dray-Spira R, et al. Risk of major adverse cardiovascular and venous thromboembolism events in patients with rheumatoid arthritis exposed to JAK inhibitors versus adalimumab: a nationwide cohort study. Ann Rheum Dis 2023;82:182-188.

© 2025 Solitano V et al. *JAMA Network Open*.

27. Kochar BD, Cheng D, Cai T, et al. Comparative Risk of Thrombotic and Cardiovascular Events with Tofacitinib and Anti-TNF Agents in Patients with Inflammatory Bowel Diseases. Dig Dis Sci 2022;67:5206-5212.
28. Ma XN, Shi MF, Wang SI, et al. Risk of dyslipidemia and major adverse cardiac events with tofacitinib versus adalimumab in rheumatoid arthritis: a real-world cohort study from 7580 patients. Front Pharmacol 2024;15:1370661.
29. Khosrow-Khavar F, Kim SC, Lee H, et al. Tofacitinib and risk of cardiovascular outcomes: results from the Safety of TofAcitinib in Routine care patients with Rheumatoid Arthritis (STAR-RA) study. Ann Rheum Dis 2022;81:798-804.
30. Meissner Y, Schafer M, Albrecht K, et al. Risk of major adverse cardiovascular events in patients with rheumatoid arthritis treated with conventional synthetic, biologic and targeted synthetic disease-modifying antirheumatic drugs: observational data from the German RABBIT register. RMD Open 2023;9.
31. Mok CC, So H, Yim CW, et al. Safety of the JAK and TNF inhibitors in rheumatoid arthritis: real world data from the Hong Kong Biologics Registry. Rheumatology (Oxford) 2024;63:358-365.
32. Molander V, Bower H, Frisell T, et al. Venous thromboembolism with JAK inhibitors and other immune-modulatory drugs: a Swedish comparative safety study among patients with rheumatoid arthritis. Ann Rheum Dis 2023;82:189-197.
33. Ozen G, Pedro S, Michaud K. The Risk of Cardiovascular Events Associated With Disease-modifying Antirheumatic Drugs in Rheumatoid Arthritis. J Rheumatol 2021;48:648-655.
34. Pawar A, Desai RJ, Gautam N, et al. Risk of admission to hospital for serious infection after initiating tofacitinib versus biologic DMARDs in patients with rheumatoid arthritis: a multidatabase cohort study. Lancet Rheumatol 2020;2:e84-e98.
35. Sendaydiego X, Gold LS, Dubreuil M, et al. Use of Biologic or Targeted Synthetic Disease-Modifying Antirheumatic Drugs and Cancer Risk. JAMA Netw Open 2024;7:e2446336.
36. Sendaydiego X, Gold LS, Dubreuil M, et al. Comparative safety of biologic and targeted synthetic disease-modifying anti-rheumatic drugs for cardiovascular outcomes in rheumatoid arthritis. Rheumatology (Oxford) 2025;64:3434-3443.
37. Sakai R, Tanaka E, Inoue E, et al. Increased risk of cardiovascular events under the treatments with Janus kinase inhibitors versus biological disease-modifying antirheumatic drugs in patients with rheumatoid arthritis: a retrospective longitudinal population-based study using the Japanese health insurance database. RMD Open 2024;10.
38. Salinas CA, Louder A, Polinski J, et al. Evaluation of VTE, MACE, and Serious Infections Among Patients with RA Treated with Baricitinib Compared to TNFi: A Multi-Database Study of Patients in Routine Care Using Disease Registries and Claims Databases. Rheumatol Ther 2023;10:201-223.

© 2025 Solitano V et al. *JAMA Network Open*.

39. Song YJ, Cho SK, You SH, et al. Association between malignancy risk and Janus kinase inhibitors versus tumour necrosis factor inhibitors in Korean patients with rheumatoid arthritis: a nationwide population-based study. RMD Open 2022;8.
40. Song YJ, Cho SK, Kim JY, et al. Risk of venous thromboembolism in Korean patients with rheumatoid arthritis treated with Janus kinase inhibitors: A nationwide population-based study. Semin Arthritis Rheum 2023;61:152214.
41. Song YK, Lee G, Hwang J, et al. Cardiovascular risk of Janus kinase inhibitors compared with biologic disease-modifying antirheumatic drugs in patients with rheumatoid arthritis without underlying cardiovascular diseases: a nationwide cohort study. Front Pharmacol 2023;14:1165711.
42. Tong X, Shen CY, Jeon HL, et al. Cardiovascular risk in rheumatoid arthritis patients treated with targeted synthetic and biological disease-modifying antirheumatic drugs: A multi-centre cohort study. J Intern Med 2023;294:314-325.
43. Desai RJ, Pawar A, Khosrow-Khavar F, et al. Risk of venous thromboembolism associated with tofacitinib in patients with rheumatoid arthritis: a population-based cohort study. Rheumatology (Oxford) 2021;61:121-130.
44. Westermann R, Cordtz RL, Duch K, et al. Cancer risk in patients with rheumatoid arthritis treated with janus kinase inhibitors: a nationwide Danish register-based cohort study. Rheumatology (Oxford) 2024;63:93-102.
45. Cho Y, Yoon D, Khosrow-Khavar F, et al. Cardiovascular, cancer, and infection risks of Janus kinase inhibitors in rheumatoid arthritis and ulcerative colitis: A nationwide cohort study. J Intern Med 2025;297:366-381.
46. Zhao SS, Riley D, Hernandez G, et al. Comparative safety of JAK inhibitors versus TNF or IL-17 inhibitors for cardiovascular disease and cancer in psoriatic arthritis and axial spondyloarthritis. Clin Ther 2025;47:293-297.
47. Aymon R, Mongin D, Guemara R, et al. Incidence of Major Adverse Cardiovascular Events in Patients With Rheumatoid Arthritis Treated With JAK Inhibitors Compared With Biologic Disease-Modifying Antirheumatic Drugs: Data From an International Collaboration of Registries. Arthritis Rheumatol 2025.
48. Silvagni E, Bortoluzzi A, Occhino G, et al. Frailty and cardiovascular safety of JAK inhibitors versus TNF inhibitors in rheumatoid arthritis: a real-world comparative study of drug effects and patient profiles. Front Pharmacol 2025;16:1565909.
49. Kao CM, Chen YJ, Chen YM, et al. Major adverse cardiovascular events or venous thromboembolism in patients with rheumatoid arthritis initiating biological or targeted synthetic disease-modifying antirheumatic drugs: a nationwide, population-based cohort study. Ther Adv Musculoskelet Dis 2025;17:1759720X251321917.
50. Tanaka Y, Kishimoto M, Sonomoto K, et al. Methotrexate, Tofacitinib, and Biologic Disease-Modifying Antirheumatic Drug Safety and Effectiveness Among Patients with Rheumatoid Arthritis in Japan: CorEvitas Registry Observational Study. Rheumatol Ther 2024;11:1237-1253.

© 2025 Solitano V et al. *JAMA Network Open*.
