# Supplementary material for: Comparative Safety of JAK Inhibitors vs TNF Antagonists in Immune-Mediated Inflammatory Diseases: A Systematic Review and Meta-Analysis
Source: JAMA Netw Open. 2025 Sep 10;8(9):e2531204. doi: 10.1001/jamanetworkopen.2025.31204 (PMC12423869; doi:10.1001/jamanetworkopen.2025.31204)
Supplement: Supplement 2. — Data Sharing Statement [file jamanetwopen-e2531204-s002.pdf]

Data Sharing Statement

Solitano. Comparative Safety of JAK Inhibitors vs TNF Antagonists in Immune-Mediated Inflammatory Diseases. *JAMA Netw Open*. Published September 10, 2025. doi:10.1001/jamanetworkopen.2025.31204

Data
Data available: No

Additional Information
Explanation for why data not available: All data needed to re-create the analysis is in the paper
